# Induction of IRAK-M in melanoma induces caspase-3 dependent apoptosis by reducing TRAF6 and calpastatin levels

**DOI:** 10.1038/s42003-020-1033-y

**Published:** 2020-06-12

**Authors:** Degui Geng, Nicholas Ciavattone, Jackline Joy Lasola, Rojesh Shrestha, Amelia Sanchez, Jitao Guo, Alexandra Vlk, Rania Younis, Lucy Wang, Alex J. Brown, Yuji Zhang, Cruz Velasco-Gonzalez, Aik-Choon Tan, Eduardo Davila

**Affiliations:** 1 Department of Medicine, Division of Medical Oncology, University of Colorado School of Medicine, Aurora, CO 80045 USA; 2 Marlene and Stewart Greenebaum Comprehensive Cancer Center, University of Maryland School of Medicine, Baltimore, MD 21201 USA; 3 Renal Electrolyte and Hypertension Division, Department of Medicine and Genetics, University of Pennsylvania, Philadelphia, PA 19104 USA; 4 Department of Oncology and Diagnostic Sciences, University of Maryland School of Dentistry, Baltimore, MD 21201 USA; 5 Department of Immunology and Microbiology, University of Colorado School of Medicine, Aurora, CO 80045 USA; 6 Department of Epidemiology and Public Health, University of Maryland School of Medicine, Baltimore, MD 21201 USA; 7 Center for Outcomes and Health Services Research, Ochsner Health System, New Orleans, LA 70115 USA; 8 University of Colorado Denver Comprehensive Cancer Center, Aurora, CO 80045 USA

**Keywords:** Melanoma, Melanoma, Apoptosis

## Abstract

Melanoma represents the most serious type of skin cancer. Although recent years have seen advances using targeted and immunotherapies, most patients remain at high risk for tumor recurrence. Here we show that IRAK-M, a negative regulator of MyD88 signaling, is deficient or low in melanoma and expression levels correlate with patient survival. Inducing IRAK-M expression using genetic approaches or epigenetic modifiers initiates apoptosis by prompting its interaction with TRAF6 via IRAK-M’s C-terminal domain. This complex recruits and degrades calpastatin which stimulates calpain activity and triggers caspase-3-dependent but caspase-8,−9-independent apoptosis. Using a drug screen, we identified compounds that induced IRAK-M expression. Administration of IRAK-M-inducing drugs reduced tumor growth in mice but was ineffective against IRAK-M knock-down tumors. These results uncover a previously uncharacterized apoptosis pathway, emphasize IRAK-M as a potential therapeutic target and suggest that the anticancer activity of certain drugs could do so through their ability to induce IRAK-M expression.

## Abstract (web summary)

Geng et al find that low levels of the anti-inflammatory molecule IRAK-M in melanoma correlates with reduced patient survival and that induced expression of IRAK-M induces caspase-3 dependent apoptosis. The identification of cytotoxic compounds associated with IRAK-M induction suggests a route to melanoma drug development.

## Introduction

Melanoma is one of the deadliest forms of cancer and is the fifth and seventh most common cancer for men and women, respectively^1^. Patients with advanced disease have a median survival of 6–12 months^2^. The recent development of several treatment options for melanoma including targeted and immunotherapies have resulted in remarkable antitumor responses. However, significant responses to these forms of therapies are limited to a minority of patients demanding the need for further generation of more effective therapies.

While interleukin-1 receptor-associated kinase-1 and −4 (IRAK) is best known for its induction of the inflammatory signaling pathway in innate immune cells, recent studies highlight its important role in modulating the expression of antiapoptotic and tumor growth factors in various cancer types including melanoma^3–9^. Of the four IRAK family members, IRAK-M is unique in that it lacks kinase activity and serves as an anti-inflammatory molecule by impeding IRAK-4,−1 signaling^5^. However, IRAK-M’s role in tumorigenesis, survival, or melanoma progression is unknown.

Through these studies, we characterized IRAK-M’s role in melanoma cell death and tease apart an unusual molecular signaling pathway that is preferentially induced in melanoma but not melanocytes. We identified several FDA-approved compounds that selectively induced IRAK-M expression in melanoma and whose cytotoxicity was associated with their ability to induce IRAK-M expression. These studies are significant as they offer the opportunity to develop more effective classes of targeted cancer therapies and suggest that the therapeutic activity of certain approved drugs is strictly dependent on their ability to induce IRAK-M expression.

## Results

### Decreased or deficient IRAK-M levels in human melanoma

**Fig. 1:**
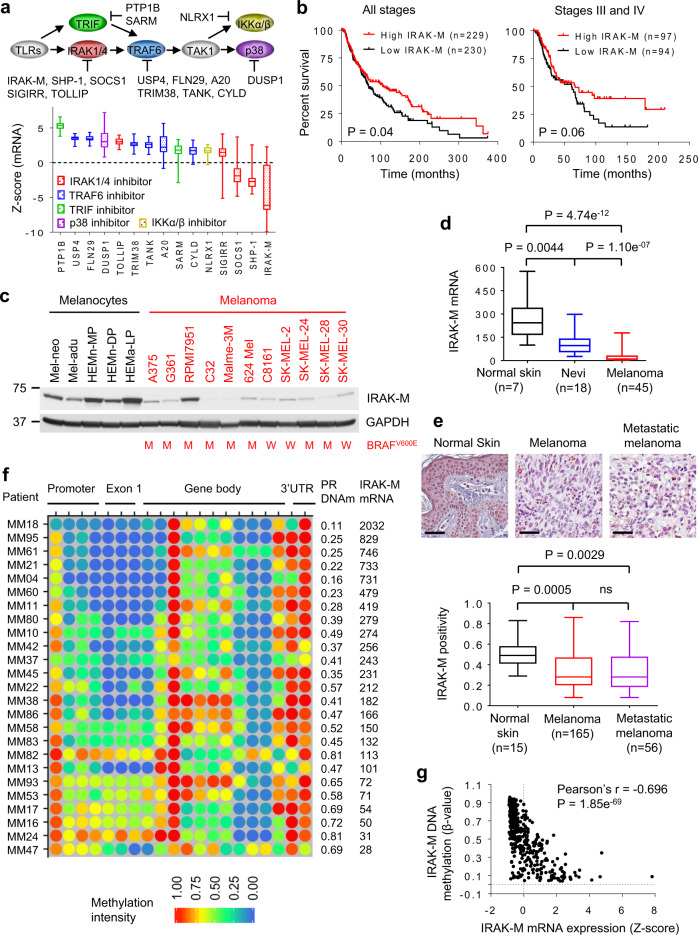
IRAK-M expression is absent or low in human melanoma. **a** mRNA expression profiles of negative regulators of TLR signaling in 56 human melanoma cell lines using the CCLE dataset. **b** Kaplan–Meier survival analyses of all and stage III/IV melanoma patients from the TCGA database. Melanoma patients were stratified into ‘high’ (above the median) and ‘low’ (below the median) IRAK-M expression. The number (*n*) in parentheses is the number of patients. *P*-values were determined with the Log-rank (Mantel-Cox) test. **c** IRAK-M protein levels were determined by western blot in human melanocytes and melanoma cell lines. The BRAF V600E mutation status is indicated as M mutation and W wild-type. Blots are representative of at least three independent experiments. **d** IRAK-M mRNA levels in normal human skin and biopsy specimens from melanoma patients with the number of samples (*n*) shown in parentheses. The analysis of IRAK-M expression was performed based on the online published microarray database (GEO GSE3189). *P*-values by two-tailed Student’s *t*-test. **e** Protein expression of IRAM-M in human normal skin and tumor tissues was detected using Immunohistochemistry (IHC) on melanoma tissue microarrays. Representative IHC images are shown (scale bars, 100 µm). Positivity was defined as the total number of positive pixels over the total number of pixels. The number of analyzed samples (*n*) is indicated in each column. *P*-values by two-tailed Student’s *t*-test. **f** DNA methylation (β-value) and mRNA levels (signal intensity) of IRAK-M in 25 melanoma patient samples obtained from the GEO database (GSE51547 and GSE22153). Promoter DNA methylation (PR DNAm) values and mRNA levels are along patient samples. **g** DNA methylation β-values and gene expression Z-scores (RNA-seq V2RSEM) of IRAK-M in TCGA skin cutaneous melanoma database consisting of 471 patient samples obtained from the cBioPortal (www.cbioportal.org). Each symbol represents one patient sample. The dotted lines indicate β-values and Z-scores of 0. Correlation was evaluated using Pearson’s correlation test. Pearson’s r and *p*-values for the correlation are indicated.

MyD88 signaling is a tightly regulated process controlled by many negative regulators at different points within the signaling cascade. Considering the link between IL-1R/MyD88-mediated inflammation and tumor progression, we investigated the expression profiles of numerous molecules known to inhibit MyD88 signaling in melanoma using data from Cancer Cell Line Encyclopedia (CCLE) (Fig. 1a, top panel). IRAK-M was found to be the least expressed of the various inhibitors investigated (Fig. 1a) and was downregulated in 46 of 56 human melanoma cell lines (Supplementary Fig. 1a). Importantly, Kaplan–Meier survival curves demonstrated that melanoma patients with decreased IRAK-M transcript levels showed reduced overall survival as compared with patients with elevated IRAK-M levels (Fig. 1b).

We investigated the protein expression profiles of MyD88 signaling inhibitors in melanoma cell lines and melanocytes; IRAK-M was consistently low or deficient in most melanoma lines (Fig. 1c). In contrast, all melanocyte lines expressed IRAK-M. The expression levels or activation status of eleven other inhibitors were also investigated but found to be variably expressed in all lines with no discernible pattern within melanoma cell lines or between melanomas and melanocytes (Supplementary Fig. 1b). IRAK-M mRNA expression profiles correlated with protein levels (Supplementary Fig. 1c). Because melanoma patients commonly harbor genetic alterations in *BRAF*, *NRAS*, *CDKN2A*, or *NF1* genes that contribute to tumor progression^10–13^, we examined potential associations between these genetic alterations and IRAK-M levels in melanoma cell lines and patient samples. However, no correlations between these genetic factors and IRAK-M expression levels could be made (Fig. 1c and Supplementary Fig. 3a). Analyses of microarray data and immunohistochemistry from melanoma patients revealed decreased IRAK-M transcript (Fig. 1d) and protein levels (Fig. 1e). Further analyses indicated that reduced transcript levels were not due to decreased mRNA stability (Supplementary Fig. 2a), changes in genomic copy number (Supplementary Fig. 2b), or variations in the *IRAK-M* promoter region (Supplementary Table 1). Diminished IRAK-M transcript levels were also observed in other cancer types including prostate, lung, ovarian and pancreatic cancer as well as glioblastoma (Supplementary Fig. 2c). DNA methylation plays a key role in regulating gene expression^14^. We investigated the *IRAK-M* DNA methylation profiles of patient samples and melanoma cell lines and found that reduced methylation within the promoter region of *IRAK-M* correlated with increased transcript levels (Fig. 1f, Supplementary Fig. 3b, c), neither did they correlate with *BRAF* or *NRAS* mutation status, nor *CDKN2A* genotype (Supplementary Fig. 3b). We also conducted a genome-wide analysis of DNA methylated sites in RPMI7951, C32, Malme-3M, and SK-MEL-28 melanoma lines and found that the *IRAK-M* promoter region were hypomethylated in RPMI7951 but hypermethylated in C32, Malme-3M, and SK-MEL-28 cells (Supplementary Fig. 4 and Supplementary Table 2). These data agree with the observations that while RPMI7951 exhibits elevated IRAK-M transcript and protein levels, C32, Malme-3M, and SK-MEL-28 show reduced levels. The data in Fig. 1g demonstrates mutual exclusivity of IRAK-M transcript levels and DNA methylation and further substantiate that IRAK-M transcription is regulated by its methylation status.

### Restoring IRAK-M expression in melanoma induces cell death

**Fig. 2:**
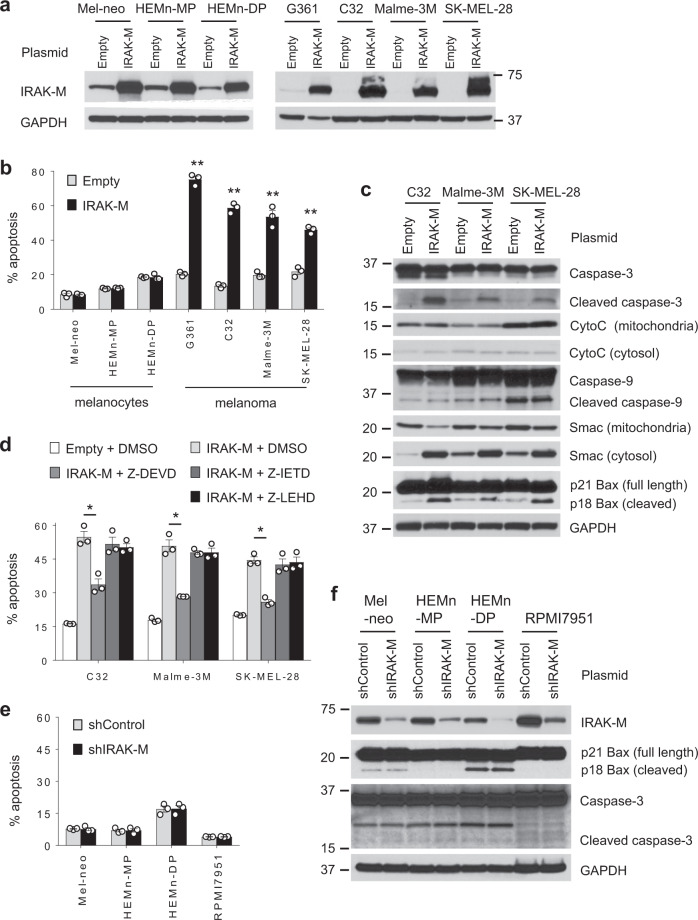
Restoring IRAK-M expression in human melanoma cell lines induces cell death. **a** RAK-M protein level was determined by western blot in human melanocytes and melanoma cell lines transfected with empty vector or *IRAK-M* construct for 24 h. Blots are representative of at least two independent experiments. **b** Human melanocytes and melanoma cell lines were transfected with a plasmid control or p*IRAK-M*, and apoptosis was measured by flow cytometry (Annexin V staining) 24 hours later (*n* = 3 per group). Results are shown as mean ± SEM. **p < 0.01 by two-tailed Student’s *t*-test. Data are representative of at least three experiments, each yielding identical results. **c** Expression levels of apoptosis-related proteins in melanoma cells transfected for 24 h were evaluated by western blot. Blots shown are representative of three independent experiments. **d** The treatment effects of 20 µM Z-DEVD-FMK (a caspase-3 inhibitor), Z-IETD-FMK (a caspase-8 inhibitor), or Z-LEHD-FMK (a caspase-9 inhibitor) in IRAK-M transfected melanoma cells for 24 h were determined using flow cytometric analysis of apoptosis (*n* = 3 per group). Results are presented as mean ± SEM. *p < 0.05 by two-tailed Student’s *t*-test. Data presented are representative of two independent experiments. **e**, **f** Melanocytes and melanoma cell line RPMI7951 were transfected with psiRNA-Control or psiRNA-hIRAK-M vector for 24 h, followed by **e** flow cytometric analysis of apoptosis (*n* = 3 per group) and **f** western blot. Results are presented as mean ± SEM. Data shown are representative of two independent experiments.

Given IRAK-4’s role in promoting cancer cell survival, we investigated IRAK-M’s part in melanoma survival following expression of IRAK-M by nucleofection, which achieved high protein expression levels in both melanomas and melanocytes (Fig. 2a). IRAK-M expression induced apoptosis in all four melanoma cell lines, as compared with control vector-transfected cells (Fig. 2b). In sharp contrast, IRAK-M expression in melanocytes did not impact cell viability despite high IRAK-M expression levels (Fig. 2b).

To better understand the signaling pathway by which IRAK-M induced cell death, we examined changes in the levels of caspases and apoptosis- and cell-survival-related proteins. Active caspase-3, which generally exists as an inactive 32-kDa zymogen and is cleaved to yield a catalytically active subunit, was detected in response to IRAK-M expression (Fig. 2c). Because cytochrome C released from stressed mitochondria can activate caspase-9, which in turn can lead to caspase-3 cleavage, we evaluated the mitochondrial and cytosolic levels of cytochrome C following IRAK-M expression. However, no changes in cytochrome C levels were observed following IRAK-M expression (Fig. 2c). Consistent with these data, IRAK-M expression did not prompt caspase-9 cleavage. FADD and caspase-8 activation represent another pathway for activating caspase-3. However, no changes in FADD or cleaved caspase-8 levels were observed in response to IRAK-M expression (Supplementary Fig. 5a). Treatment of melanoma cells with specific caspase inhibitors demonstrated that IRAK-M-induced cell death occurred in a caspase-3-dependent but caspase-8- and −9-independent fashion (Fig. 2d). In contrast, IRAK-M knockdown in melanocytes or three melanoma lines RPMI7951, A101D, and HS294T, which naturally expressed high IRAK-M levels, did not induce cell death (Fig. 2e, Supplementary Fig. 5b, c). IRAK-M knockdown instead promoted melanocyte proliferation (Supplementary Fig. 5d).

The proapoptotic molecule Smac/DIABLO, released from stressed mitochondria, induces apoptosis by inhibiting the antiapoptotic function of the inhibitor of apoptosis proteins (IAPs) and this represents an alternate pathway for activating caspase-3. IRAK-M expression resulted in reduced mitochondrial Smac levels but substantially increased cytosolic Smac levels (Fig. 2c). These data indicate that IRAK-M modulates the level of mitochondrial membrane protein(s) that regulates the release of Smac into the cytosol. A key protein involved in the release of mitochondrial Smac is Bax, which exists in cytosolic latent full-length form but in response to cellular stress becomes cleaved to the active form, which translocates to the mitochondria membrane and mediates Smac release to the cytosol. IRAK-M expression resulted in increased levels of cleaved Bax in melanomas (Fig. 2c). We next evaluated the upstream signal responsible for activating Bax. Bid is a proapoptotic Bcl2 family member that becomes activated by caspase-8 to the active form tBid, and is known to activate Bax^15^. However, no differences in Bid or tBid levels were observed in IRAK-M−expressing cells (Supplementary Fig. 5a). These findings agree with our observations that IRAK-M expression did not change caspase-8 protein levels. We also examined whether IRAK-M provoked changes in the levels of other proapoptotic proteins including Bad, Bak, Bim and pro-survival proteins, Bcl2, Bcl-xL, MCL1, and Survivin (Supplementary Fig. 5a). However, the levels of each of these proteins remained similar in IRAK-M−expressing and control melanoma cells. IRAK-M silencing did not reduce the cleavage of Bax and caspase-3 in melanocytes and RPMI7951 melanoma (Fig. 2f).

### IRAK-M provokes calpain-mediated melanoma cell death

**Fig. 3:**
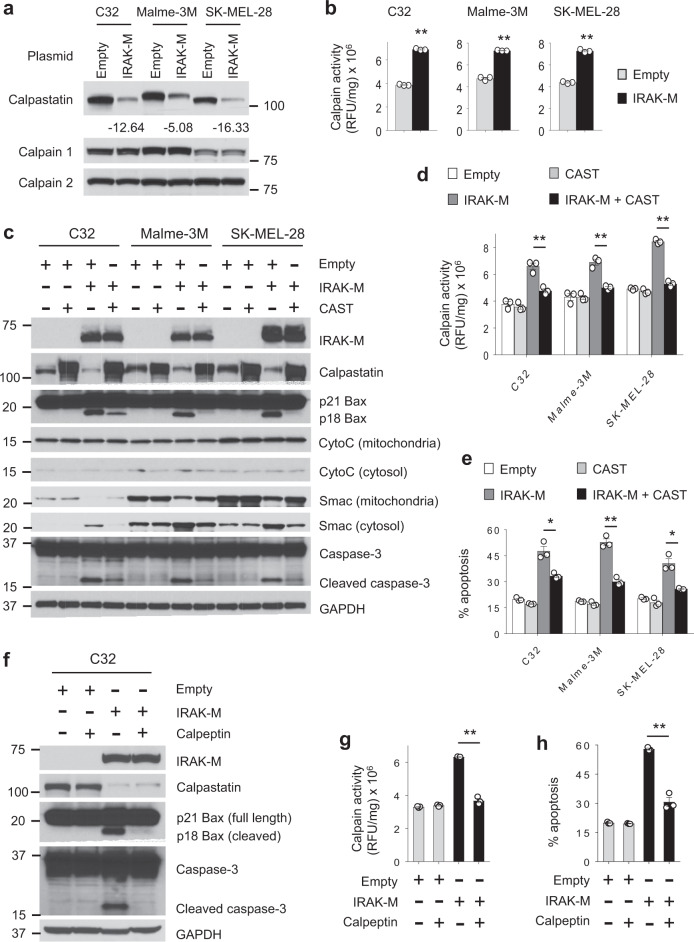
IRAK-M–induced apoptosis in melanoma is mediated through calpastatin and calpain. **a** Western blot analysis of protein levels of calpastatin, calpain 1 and calpain 2 in melanoma cells transfected with a control or *IRAK-M* plasmid for 24 h. Changes in calpastatin protein levels in transfected cells are shown. Blots shown are representative of three independent experiments. **b** Calpain activity in melanoma cells is shown as relative fluorescent units/mg total protein using a fluorescence-based calpain activity assay 24 h after transfection (*n* = 3 per group). Data are presented as mean ± SEM. **p < 0.01 by two-tailed Student’s *t*-test. Results are representative of two independent experiments. **c** Expression levels of apoptosis-related proteins in melanoma cells 24 h after transfection with *IRAK-M* and/or *CAST* plasmids by Western blot. Blots are representative of at least two independent experiments. **d** The calpain activity assay was used to measure calpain activity in melanoma cells transfected for 24 h (*n* = 3 per group). Results are presented as mean ± SEM. **p < 0.01 by two-tailed Student’s *t*-test. Data are representative of two independent experiments. **e** Apoptosis of melanoma cells was determined by flow cytometry (PI and Annexin V staining) 24 h after transfection (*n* = 3 per group). Results are shown as mean ± SEM. *p < 0.05 and **p < 0.01 by two-tailed Student’s *t*-test. Data are representative of at least two independent experiments. **f–h** The effects of 20 µM calpeptin (a calpain inhibitor) treatment in IRAK-M transfected C32 cells for 24 h were evaluated using **f** western blot, **g** calpain activity assay (*n* = 3 per group), and **h** apoptosis analysis (*n* = 3 per group). Results are presented as mean ± SEM. **p < 0.01 by two-tailed Student’s *t*-test. Data presented are representative of at least two independent experiments.

Previous studies highlighted an alternate signaling cascade leading to Bax activation. In this pathway, calpain–a calclium-dependent, cytosolic cysteine protease activates Bax^16,17^. Calpain’s activity is kept in check through its interaction with calpastatin^16^. As shown in Fig. 3a, calpastatin protein levels were considerably diminished following IRAK-M expression. However, calpain 1 and 2 levels remained unchanged between control– and IRAK-M–transfected cells (Fig. 3a). Because calpain activity is regulated through its interaction with calpastatin and not necessarily due to changes in protein levels, we investigated calpain activity from transfected melanoma cells and found that IRAK-M expression increased calpain activity (Fig. 3b). Calpain activity was determined using a fluorogenic assay in which 7-amino-4-methylcoumarin (AMC) is released from the synthetic substrate, Suc-LLVY-AMC, upon cleavage with calpain and measured fluorometrically. Consistent with the finding that human melanocytes were not vulnerable to high IRAK-M levels, the amount of apoptosis-related proteins remained unchanged in IRAK-M transfected melanocytes as compared to control cells (Supplementary Fig. 5e).

To confirm calpastatin’s role in IRAK-M–mediated apoptosis, we examined whether maintaining calpastatin levels would abrogate apoptosis. As expected, IRAK-M expression reduced calpastatin protein levels, increased Bax cleavage, promoted the release of Smac and induced the activation of caspase-3 (Fig. 3c). However, restoring calpastatin levels prevented IRAK-M–mediated activation of Bax in Malme-3M and SK-MEL-28 melanomas and substantially reduced cleaved Bax levels in C32 cells. Restoring calpastatin considerably diminished IRAK-M–induced Smac release and caspase-3 activation (Fig. 3c) and this was accompanied by reduced calpain activity (Fig. 3d) and cell death (Fig. 3e).

Considering that IRAK-M mediates apoptosis via calpain, we sought to gain definitive proof of calpastatin’s and calpain’s roles in this process. IRAK-M-expressing cells were cultured in the presence of the calpain inhibitor calpeptin, and calpain activity and apoptosis were measured. Calpeptin prevented IRAK-M from activating Bax and caspase-3 (Fig. 3f). Further, calpeptin prevented IRAK-M-mediated calpain activity and cell death (Fig. 3g, h). In contrast, control-transfected melanoma cells were not impacted by the presence of calpeptin. Collectively, these data indicate that IRAK-M-induced cell death is mediated by its ability to regulate calpain activity, which activates Bax and allows Smac to translocate from the mitochondria to the cytosol where it activates Caspase-3.

### Interaction of IRAK-M and TRAF6 initiates melanoma apoptosis

**Fig. 4:**
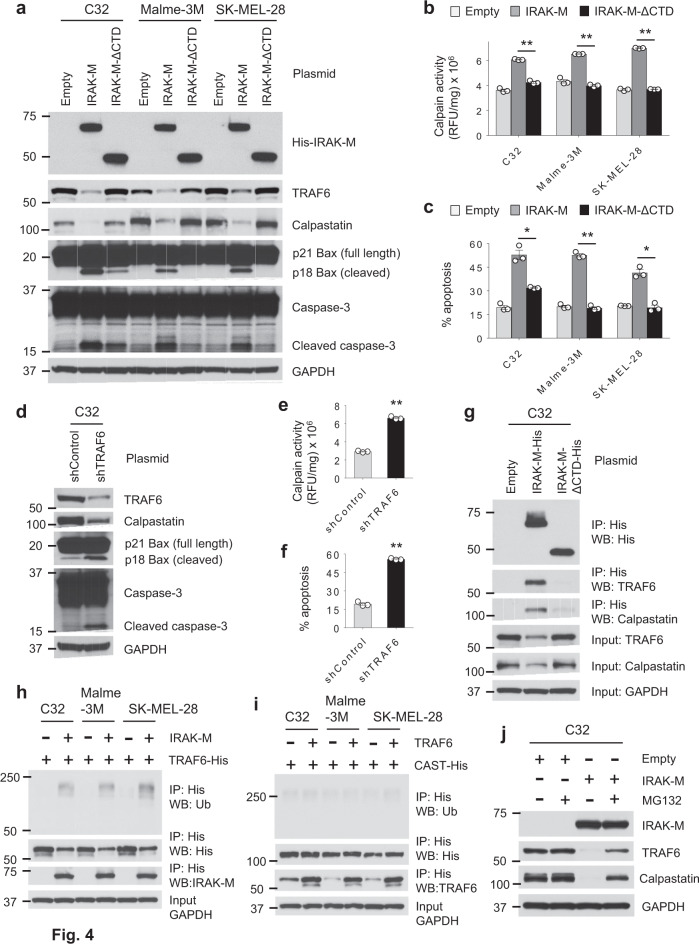
IRAK-M-induced apoptosis in human melanoma depends on TRAF6-mediated recruitment of calpastatin. **a** Expression levels of indicated proteins were detected by Western blot in melanoma cells 24 h after transfection with empty vector, WT His-*IRAK-M* or His-*IRAK-M*-ΔCTD (a truncated His-*IRAK-M* variant lacking the entire CTD domain). Blots are representative of three independent experiments, each yielding identical trends. **b** 24 h after transfection, calpain activity (relative fluorescent units/mg total protein) in melanoma cells was evaluated using the fluorescence-based calpain activity assay (*n* = 3 per group). Data are shown as mean ± SEM. **p < 0.01 by two-tailed Student’s *t*-test. Results are representative of two independent experiments. **c** Apoptosis of melanoma cells transfected for 24 h was measured by staining cells with PI and Annexin V, followed by flow cytometry (*n* = 3 per group). Results are shown as mean ± SEM. *p < 0.05 and **p < 0.01 by two-tailed Student’s *t*-test. Data shown are representative of at least two independent experiments. **d**–**f** The effects of TRAF6 knockdown (shTRAF6) in C32 cells 24 h after transfection were determined by **d** western blot, **e** calpain activity assay (*n* = 3 per group), and **f** apoptosis analysis (*n* = 3 per group). Results are presented as mean ± SEM. **p < 0.01 by two-tailed Student’s *t*-test. Data presented are representative of at least two independent experiments. **g**–**i** Melanoma cells were transfected with the indicated vectors for 18 h and collected for IP assay and western blot. Cell lysates were subjected to IP assay using sepharose bead conjugated His-tag mouse antibody and western blot using the indicated antibodies. Images shown here are representative of at least two independent experiments. **j** Western blot was performed to detect the expression levels of TRAF6 and calpastatin proteins in IRAK-M transfected C32 cells treated with 20 µM of the proteasome inhibitor (R)-MG132 for 24 h. Blots are representative of at least two independent experiments.

TRAF6 participates in a wide array of protein–protein interactions and possesses nonconventional E3 ubiquitin ligase activity. The C-terminal domain on IRAK-1 and −2 can interact with TRAF6^4,18^. However, it is not currently known if IRAK-M can directly interact with TRAF6. Because TRAF6 ubiquitination can result in its degradation^19^, we assessed the impact that IRAK-M expression had on TRAF6 protein levels in melanoma cells and what role IRAK-M’s C-terminal domain played in promoting cell death. Melanoma cells were transfected with control plasmid, *IRAK-M* or a plasmid coding for *IRAK-M* with a C-terminal deletion (IRAK-M-ΔCTD). IRAK-M expression drastically decreased TRAF6 protein levels (Fig. 4a). However, eliminating the C-terminal domain of IRAK-M prevented TRAF6 degradation. Furthermore, IRAK-M but not IRAK-M-ΔCTD expression reduced calpastatin levels resulting in the activation of Bax and caspase-3. Consistent with these data, melanoma cells expressing IRAK-M-ΔCTD lacked an ability to stimulate calpain activity (Fig. 4b) and accordingly, demonstrated impaired aptitude for inducing cell death (Fig. 4c).

To our knowledge, the connection between IRAK-M–mediated TRAF6 depletion and melanoma cell death is previously undescribed. To determine whether TRAF6 reduction alone was enough to mimic the effects of IRAK-M expression, we evaluated cell survival following TRAF6 knockdown. Knocking down TRAF6 reduced calpastatin levels and increased the activation of Bax and caspase-3, as compared with control cells transfected with scramble shRNA (Fig. 4d). Consistent with IRAK-M’s ability to induce cell death, TRAF6 knockdown increased calpain activity and induced apoptosis (Fig. 4e, f). To confirm that TRAF6 interacts with IRAK-M and calpastatin, we immunoprecipitated poly-Histidine-IRAK-M and evaluated TRAF6 and calpastatin association by western blot. Both TRAF6 and calpastatin were detected in immunoprecipitates suggesting the association of IRAK-M with TRAF6 and calpastatin (Fig. 4g). In contrast, deleting IRAK-M’s C-terminal domain prevented TRAF6 and calpastatin from forming a complex with IRAK-M. Immunoprecipitating TRAF6 revealed that expression of IRAK-M promoted K63-linked TRAF6 polyubiquitination and was associated with TRAF6 diminution (Fig. 4h). However, TRAF6 expression did not promote polyubiquitination of calpastatin (Fig. 4i). The proteasome inhibitor (R)-MG132 reduces ubiquitin-mediated protein degradation in cells. That K63-linked TRAF6 polyubiquitination results in its degradation is further validated in experiments demonstrating that treatment of cells with MG132 suppressed IRAK-M-induced TRAF6 and calpastatin degradation (Fig. 4j).

Collectively, these results suggest that IRAK-M expression leads to the formation of a complex that includes IRAK-M, TRAF6 and calpastatin, which is mediated via IRAK-M’s C-terminal domain. This complexation results in the ubiquitination and degradation of TRAF6 and calpastatin depletion.

### Drugs that regulate IRAK-M expression and cell death

**Fig. 5:**
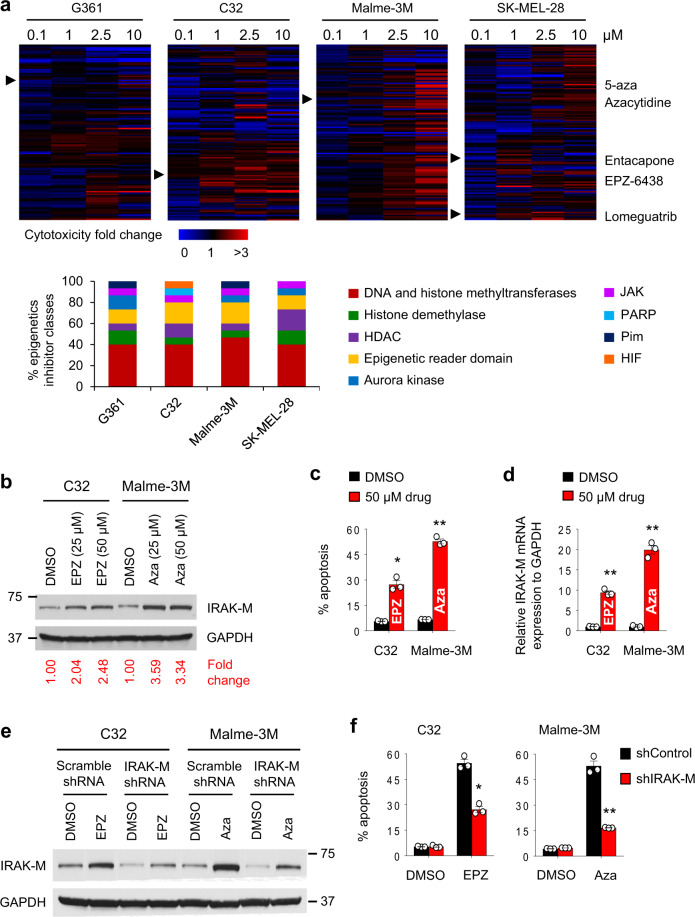
EPZ-6438 and azacytidine induce C32 and Malme-3M apoptosis via an IRAK-M-dependent mechanism. **a** Heatmaps demonstrating melanoma cell death following treatment with 128 epigenetic compounds at the indicated concentrations for 48 h as measured using a fluorescence-based CellTox green cytotoxicity assay. All values were normalized to DMSO values, which were set to 1, and heatmaps are presented based on fold changes. Lower panel shows percentages of epigenetics inhibitor classes capable of inducing apoptosis in melanoma cells in an epigenetics compound library. **b** Expression levels of IRAK-M protein were evaluated by western blot 72 h after C32 and Malme-3M cells were treated with EPZ-6438 (EPZ) and azacytidine (Aza), respectively (25 or 50 µM). Changes in IRAK-M protein expression levels in DMSO and drug-treated melanoma cells are shown. All values were normalized to DMSO values, which were set to 1. Blots presented are representative of three independent experiments. **c** 72 h after melanoma cells were cultured with 50 µM EPZ-6438 or Azacytidine, cells were stained with PI and Annexin V and analyzed by flow cytometry (*n* = 3 per group). Data are shown as mean ± SEM. *p < 0.05 and **p < 0.01 by two-tailed Student’s *t*-test. Results are representative of three independent experiments. **d** The relative mRNA levels of IRAK-M to GAPDH were determined by RT-PCR in melanoma cells 72 h after 50 µM EPZ-6438 or Azacytidine treatment (*n* = 3 per group). The relative IRAK-M mRNA values in DMSO treated C32 and Malme-3M cells were set to 1. Data are shown as mean ± SEM. **p < 0.01 by two-tailed Student’s *t*-test. Results are representative of two independent experiments. **e**, **f** Stable IRAK-M knockdown C32 and Malme-3M cells were treated with 75 µM EPZ-6438 and 50 µM azacytidine, respectively, for 48 h, followed by **e** western blot and **f** flow cytometric analysis of apoptosis (*n* = 3 per group). Results are presented as mean ± SEM. *p < 0.05 and **p < 0.01 by two-tailed Student’s *t*-test. Data shown are representative of at least two independent experiments.

Given the strong associations between DNA methylation, IRAK-M transcription, and melanoma cell death, we focused on the translational aspects of these findings. We investigated the effects of 128 epigenetic modifiers on cell death in four melanoma cell lines and identified a diverse set of compounds that provoked cell death (Fig. 5a). Most compounds that induced cell death exhibited DNA and/or histone methyltransferase activity (Fig. 5a, bottom panel). We next investigated the association between cell death and IRAK-M protein induction using the top 15 drugs that induced cell death (Supplementary Fig. 6). We identified several drugs, including EPZ-6438 and azacytidine, that induced IRAK-M expression and apoptosis in G361, C32, Malme-3M, and SK-MEL-28 (Fig. 5b, c, Supplementary Fig. 6). EPZ−6438 and azacytidine increased IRAK-M transcript levels (Fig. 5d). In agreement with these data, we observed altered DNA methylation patterns within the IRAK-M exon 1 region of treated cells (Supplementary Table 2).

To confirm that IRAK-M contributed to EPZ-6438’s and azacytidine’s ability to induce cell death, we knocked down IRAK-M and evaluated cell death in drug-treated and untreated cells. IRAK-M^shRNA^ reduced drug-induced IRAK-M protein levels (Fig. 5e) and this correlated with a reduced ability for drugs to induce cell death (Fig. 5f). In contrast, in control-transfected C32 and Malme-3M, EPZ-6438 and azacytidine strongly induced IRAK-M expression levels and resulted in melanoma cell death (Fig. 5e, f). The association between a drug’s propensity to incite cell death and stimulate IRAK-M expression was examined in other melanoma cell lines and with various other drugs (Supplementary Fig. 7a–c). Importantly, none of the drugs that induced cell death and IRAK-M expression in melanoma, did so in melanocytes (Supplementary Fig. 8a–d). Also, restoring calpastatin expression or addition of calpeptin reduced calpain activity and apoptosis in azacytidine-treated Malme-3M and EPZ-6438-treated C32 cells (Supplementary Fig. 9a–f) highlighting calpastatin’s and calpain’s roles in the drug’s ability to activate this cell death signaling pathway. Collectively, these data suggest that the cytotoxic effects of EPZ-6438 and azacytidine on C32 and Malme-3M melanomas are intertwined with their ability to induce IRAK-M expression.

### The antitumor activity of azacytidine is mediated via IRAK-M

**Fig. 6:**
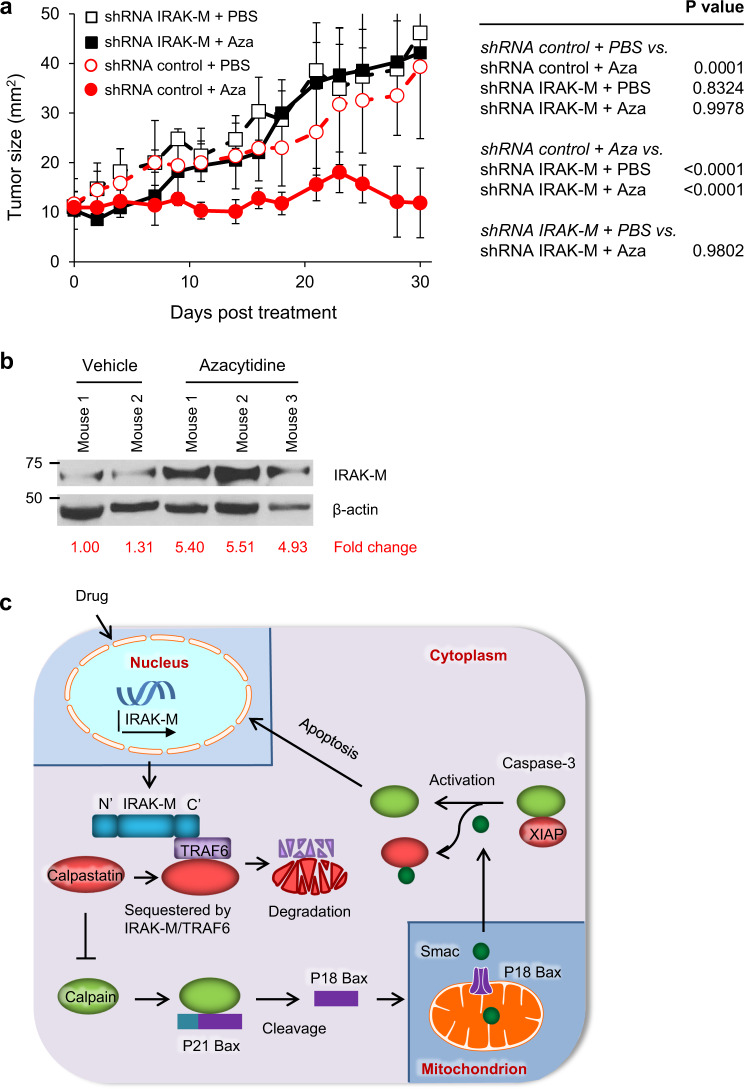
IRAK-M mediates azacytidine’s antitumor activity against an established human melanoma tumor in mice. **a** NSG mice (*n* = 5 mice per group) were injected subcutaneously with 6 × 10^6^ Malme-3M cells, followed by treatment with 2 mg/kg azacytidine (Aza) or PBS intraperitoneally when tumors reached a size of approximately 10 mm^2^. Tumor sizes (mm^2^) were calculated by measuring perpendicular by longitudinal diameter. Tumor growth data were analyzed using 2-way ANOVA (mean ± SEM). **b** Expression levels of IRAK-M protein in melanoma tumor samples from treated NSG mice were detected by western blot. Changes in IRAK-M protein expression levels in PBS and azacytidine-treated tumor samples are shown. All values were normalized to the value of vehicle-treated mouse 1, which was set to 1. Representative results from two experiments are showed. **c** Diagram depicting how IRAK-M signaling induces apoptosis in human melanoma. IRAK-M induces cell death through a previously uncharacterized apoptosis signaling pathway in melanoma. Induction of IRAK-M protein prompts its interaction with TRAF6 protein through the binding of the C-terminal domain (CTD) of IRAK-M. This IRAK-M/TRAF6 complex subsequently interacts with and sequesters calpastatin, which in turn increases the degradation of TRAF6 and calpastatin proteins rapidly. Decreased calpastatin protein level prompts calpain to interact with and activate Bax, resulting in increased cleavage of full-length p21 Bax to p18 Bax. p18 Bax is translocated from the cytosol to the outer membranes of mitochondria, inducing increased release of Smac/DIABLO from mitochondria into the cytosol. Released Smac/DIABLO binds and dissociates the inhibitor of apoptosis protein 3 XIAP from caspase-3, which in turn promotes caspase-3 autocleavage and subsequent apoptosis.

We investigated the impact that azacytidine-induced IRAK-M expression in melanoma had on tumor progression in vivo. Malme-3M cells were engineered to stably knockdown IRAK-M (Malme-3M^shRNA IRAK-M^) or express scramble RNA (Malme-3M^shRNA control^). Azacytidine or vehicle control were administered when the tumor reached ~10 mm^2^. Azacytidine, which induces IRAK-M in Malme-3M^shRNA control^ cells, reduced the tumor growth kinetics as compared with vehicle control-treated mice (Fig. 6a). In sharp contrast, preventing the expression of IRAK-M in melanoma (using Malme-3M^shRNA IRAK-M^) abrogated the antitumor activity of azacytidine. Azacytidine treatment correlated with the induction of IRAK-M expression in freshly explanted melanoma tumors (Fig. 6b). These results indicate that the cytotoxic effect of azacytidine on Malme-3M tumors in vivo is associated with its ability to induce IRAK-M expression in cancer cells.

## Discussion

An understanding of the signaling pathways that initiate cancer cell death while sparing non-cancerous tissues is a key goal in the effort toward developing effective targeted therapies. Here, we demonstrate that while melanomas are generally deficient in or express low IRAK-M levels, melanocytes contain elevated levels of IRAK-M. IRAK-M was initially thought to reside solely in monocyte and macrophage populations, where it is induced via TLR stimulation and functions as a negative regulator of TLR signaling^20–22^. However, through these studies, we offer insights demonstrating that IRAK-M plays a critical role in regulating melanoma cell death and yet has no effect on melanocyte apoptosis. We establish that IRAK-M is regulated at the transcriptional level via mechanisms that involve DNA methylation, and that its expression can be upregulated in numerous melanoma cell lines using epigenetic modifiers such as azacytidine, EPZ-6438 and others. In vivo studies using melanoma cells engineered to stably knockdown IRAK-M demonstrate that azacytidine’s ability to reduce tumor growth strictly depends on IRAK-M expression. It is also worth noting that the cytotoxic effects of drug-induced IRAK-M occurred in melanoma cells but not melanocytes. These observations suggest that cell-specific effects of IRAK-M are determined by the activation of signaling pathways that are intact in melanoma but deficient in melanocytes.

We described a previously uncharacterized signaling pathway by which IRAK-M expression induces melanoma cell death. The sequence of events leading to apoptosis are shown in Fig. 6c and are as follows. Induction of IRAK-M protein prompts its interaction with the TRAF6 E3 ligase via IRAK-M’s C-terminal domain. This complex subsequently interacts with and sequesters calpastatin, resulting in the rapid diminution of TRAF6 and calpastatin proteins. Knocking down TRAF6 provided evidence that TRAF6 is the critical factor for reducing calpastatin protein levels. Inhibiting proteasome activity, which prevented TRAF6 degradation, highlights an important role for its depletion in mediating cell death. It is worth noting that although TRAF6 has been shown to regulate apoptosis in other cell types, the mechanisms by which TRAF6 resulted in melanoma cell death is unique. For example, He et al demonstrate that the C-terminal domain of TRAF6 interacts directly with the N-terminal RING domain of caspase-8, and that this interaction triggers apoptosis in HeLa cells^23^. By contrast, in melanoma IRAK-M–mediated apoptosis occurred independent of caspase-9 and caspase-8. Instead, the depletion of calpastatin prompts calpain to interact with and activate Bax, which regulates the release of Smac/DIABLO from the mitochondria to the cytosol.

We explored underlying mechanisms by which IRAK-M expression induces melanoma cell death while sparing melanocytes. High IRAK-M expression drastically decreased TRAF6 and calpastatin proteins in melanoma cells but did not change the levels in melanocytes. The reduced levels of these proteins resulted in the activation of Bax and caspase-3 in melanoma but not melanocytes. The results highlight that IRAK-M signaling is distinct between melanoma cells and melanocytes. However, underlying mechanisms responsible for these differences are unknown but currently under investigation by our group.

While we observed that IRAK-M expression induced apoptosis in melanoma, elegant studies by Kesselring et al. demonstrated that IRAK-M in colorectal cancer cells (CRCs) contributes to tumor progression^24^. They establish that IRAK-M in CRCs supports barrier breach at the tumor site and that IRAK-M expression is in part regulated by exposure to the microbiome. In immune cells, IRAK-M is induced upon chronic exposure to TLR stimulation and its expression is largely dependent on the presence of inflammatory molecules such as TNFα^5,25^. It is plausible that since TLR-induced signaling inhibitors that target the MyD88 signaling pathway, such as IRAK-M, are produced in response to chronic TLR signaling, CRC cells develop mechanisms to cope with increased IRAK-M expression without undergoing cell death. One such survival mechanism that could occur through IRAK-M is the induction and stabilization of the STAT3 pathway, which is critical for CRC cell survival and tumor progression. Indeed, Kesselring et al. demonstrated that IRAK-M^-/-^ tumors were less proliferative in vivo than IRAK-M proficient tumors^24^. In contrast, knocking down IRAK-M in melanoma did not impact tumor growth but was indispensable for the ability of IRAK-M-inducing drugs to kill melanoma cells. It is conceivable that differences in the use of IRAK signaling pathways by different cancers is influenced by the exposure to and duration of specific microbes. However, identifying such distinctions would be of great interest for discovering unique signaling pathways and developing more effective targeted therapies for each of these cancers.

In summary, our studies reveal that IRAK-M is deficient or expressed at low levels in melanoma and that its induction using epigenetic modifying drugs induces cell death. To our knowledge, these findings reveal a previously uncharacterized apoptosis signaling pathway in melanoma and present a promising approach for targeted cancer therapy by identifying strategies that selectively modulate IRAK-M expression in cancer cells.

## Methods

### Mice and cell lines

NOD.Cg-*Prkdc*^*scid*^ *Il2rg*^*tm1Wjl*^/SzJ (NSG) mice were purchased from the Jackson Laboratory. All mouse experiments have been reviewed and approved by the Institutional Animal Care and Use Committee (IACUC) of the University of Maryland Baltimore. A375, G361, RPMI7951, C32, Malme-3M, SK-MEL-2, SK-MEL-24, SK-MEL-28, A101D, and HS294T melanoma cell lines were purchased from American Type Culture Collection (ATCC). 624 Mel and C8161 cell lines were provided by Dr. Suzanne Ostrand-Rosenberg (University of Maryland, Baltimore County) and SK-MEL-30 by Dr. Thomas Hornyak (University of Maryland, Baltimore). Mel-neo (primary human epidermal melanocytes, neonatal) and Mel-adu (primary human epidermal melanocytes, adult) cells were obtained from ATCC, and HEMn-MP and HEMn-DP (human epidermal melanocytes, neonatal) and HEMa-LP (human epidermal melanocytes, adult) cells from Cascade Biologics. C32, RPMI7951, and SK-MEL-2 cells were cultured in Eagle’s minimum essential medium supplemented with 10% fetal bovine serum and 1% penicillin/streptomycin; A375, SK-MEL-28, A101D, and HS294T in Dulbecco’s modified Eagle’s medium, and G361 in McCoy’s 5a modified medium, supplemented with 10% fetal bovine serum and 1% penicillin/streptomycin; Malme-3M in Iscove’s modified Dulbecco’s medium with 20% fetal bovine serum and 1% penicillin/streptomycin; SK-MEL-24 in Eagle minimum essential medium with nonessential amino acids, sodium pyruvate, 15% fetal bovine serum, and penicillin/streptomycin; 624mel, C8161, and SK-MEL-30 in RPMI-1640 with 10 mM HEPES, 10% fetal bovine serum, and penicillin/streptomycin. Mel-neo cells were grown in dermal cell basal medium supplemented with melanocyte growth kit components and Mel-adu in dermal cell basal medium with adult melanocyte growth kit components. HEMn-MP and HEMn-DP cells were cultured in medium 254 supplemented with human melanocyte growth supplement (HMGS), and HEMa-LP in medium 254 supplemented with human melanocyte growth supplement-2 (HMGS-2). All cell lines were maintained at 37 °C in a humid environment with 5% CO_2_. Cells were cultured for no more than 10 passages and not tested for mycoplasma contamination. Cell lines were authenticated using quantitative PCR (qPCR) for three markers (gp100, MART-1, and TYRP1).

### Western blot

IRAK-M (#4369, 1:1000), Bax (#2772, 1:1000), Smac/Diablo (#15108, 1:1000), cytochrome c (#4272, 1:1000), caspase-3 (#9668, 1:1000), caspase-8 (#4790, 1:1000), caspase-9 (#9508, 1:1000), TRAF6 (#8028, 1:1000), calpastatin (#4146, 1:1000), calpain 1 (#2556, 1:1000), calpain 2 (#2539, 1:1000), phospho-FADD (Ser194) (#2781, 1:1000), FADD (#2782, 1:1000), Bid (#2002, 1:1000), phospho-Bad (Ser112) (#9296, 1:1000), Bad (#9292, 1:1000), Bak (#12105, 1:1000), Bim (#2933, 1:1000), Bcl2 (#4223, 1:1000), Bcl-XL (#2764, 1:1000), Mcl1 (#4572, 1:1000), survivin (#2808, 1:1000), SOCS1 (#3950, 1:1000), Tollip (#4748, 1:1000), phospho-SHP-1 (Tyr564) (#8849, 1:1000), SHP-1 (#3759, 1:1000), A20 (#4625, 1:1000), CYLD (#8462, 1:1000), TANK (#2141, 1:1000), USP4 (#2651, 1:1000), His-Tag (#2365, 1:2000), His-Tag (#4079, 1:20), K63-linkage polyubiquitin (Ub, #5621, 1:1000), and GAPDH (#2118, 1:2000) antibodies from Cell Signaling Technology were used. SIGIRR (ab233146, 1:1000), ST2 (ab25877, 1:1000), phospho-PTP1B (Ser50) (ab88472, 1:1000), PTP1B (ab88481, 1:1000), and TRIM38 (ab69977, 1:1000) antibodies were from Abcam.

Whole-cell protein extracts were prepared from human epidermal melanocytes or melanoma cells using ice-cold protein extraction buffer (Full Moon BioSystems) containing protease and phosphatase inhibitor cocktail (Thermo Scientific). Twenty-five or fifty microgram of protein sample was loaded in each lane, electrophoresed by SDS-PAGE and transferred to a PVDF membrane. Blots were incubated sequentially with mouse or rabbit primary antibodies and horseradish peroxidase-conjugated secondary antibody and detected using enhanced chemiluminescence substrate (Plus-ECL; PerkinElmer). To evaluate the release of cytochrome c and Smac/DIABLO, mitochondrial and cytosolic fractions were isolated using Mitochondrial Isolation Kit for Cultured Cells (Thermo Scientific) according to the manufacturer’s protocol. The supernatant (cytosolic fraction) was collected and the mitochondrial pellet was boiled with SDS-PAGE sample buffer, and then subjected to western blot analysis. The full, uncropped Western blot images are shown in Supplementary Fig. 10.

### Quantitative real-time PCR (qPCR)

To determine IRAK-M mRNA stability, 1 × 10^6^ human epidermal melanocytes and melanoma cells were treated with 100 ng/ml actinomycin D in 6-well plates. After 0, 2, 4, 6, or 8 h, cells were harvested, and total RNA was extracted and subjected to quantitative PCR. qPCR was further used to examine DNA copy number variations of *IRAK-M*. Genomic DNA was isolated from melanocytes and melanoma cells, also subjected to qPCR analysis. Primers used for qPCR are listed in Supplementary Table 3.

### Immunohistochemical staining

Malignant Melanoma tissue microarrays (US Biomax) were deparaffinized and hydrated. Tissues underwent antigen retrieval using citrate buffer heated to 95 °C for 20 min, followed by blocking in 1.5% normal serum for 2 h. IRAK-M primary antibody (Thermo Fisher Scientific) incubation was done overnight in blocking buffer using 1:100 dilution at 4 °C. Secondary biotinylated antibody was incubated on tissues for 45 min, followed by 30-min incubation with VECTASTAIN Elite ABC reagent (Vector Laboratories) and 8-min incubation with ImmPACT NovaRED peroxidase substrate (Vector Laboratories). Slides were then rinsed in tap water, counterstained, and mounted. Whole slides were scanned at ×40 using the Aperio CS2 (Leica Biosystems) and analyzed by Aperio ImageScope software (Leica Biosystems).

### Microarray, CCLE dataset, and TCGA database analyses

To assess the transcript level of IRAK-M in human normal and various tumor tissues, the analysis of gene expression was performed based on publicly available microarray datasets downloaded from Gene Expression Omnibus (melanoma, GEO GSE3189; prostate cancer, GSE35988; lung cancer, GSE10072; ovarian cancer, GSE26712; glioblastoma, GSE7696; and pancreatic cancer, GSE16515). mRNA levels (RNA-seq) of negative regulators of TLR signaling and DNA methylation of *IRAK-M* gene were downloaded from the Cancer Cell Line Encyclopedia (CCLE) dataset (56 human melanoma cell lines). Kaplan–Meier analysis of overall survival of melanoma patients was performed based on The Cancer Genome Atlas (TCGA) skin cutaneous melanoma RNA-seq database. From the original melanoma patients, 459 patients with available survival data were used for the Kaplan–Meier curves. Patients were stratified into ‘above’ (high) and ‘below’ (low) IRAK-M median. *P*-value was assessed by Log-rank (Mantel-Cox) test.

### Methylation array

Genome-wide DNA methylation analysis of over 850,000 methylation sites was performed using the Infinium MethylationEPIC BeadChip (Illumina) in RPMI7951, C32, Malme-3M, SK-MEL-28, EPZ-6438-treated C32, and azacytidine-treated Malme-3M cells. 5 × 10^5^ C32 and Malme-3M cells were treated with 50 µM EPZ-6438 and azacytidine respectively in 6-well flat-bottom plates containing 3 ml of culture medium for 72 h. Cells were collected, genomic DNA was extracted using the Wizard Genomic DNA Purification Kit (Promega) and was sodium bisulfite treated using the EZ DNA Methylation Kit (Zymo Research) according to instructions from manufacturers. DNA methylation levels were analyzed by GenomeStudio Software 2011.1 (Illumina). Methylation data are available in the Gene Expression Omnibus (GEO) database (accession number GSE143614).

### Plasmids

Plasmids pUNO1-h*IRAK-M* for IRAK-M overexpression, psiRNA-hIRAK-M for IRAK-M knockdown, and psiRNA-hTRAF6 for TRAF6 knockdown were obtained from InvivoGen. The expression plasmid pCI-h*CAST* was constructed starting from the amplification of human *CAST*. The two partial *CAST* fragments were amplified by polymerase chain reaction (PCR) using cDNA as a template. The two pairs of primers used here were sense 1 (5’-TCGTCGACGCCATGTCCCAGCCCGGCCAGAAG-3’) and antisense 1 (5’-GCCTCGAGTGCTTGATCACTCAT-3’), and sense 2 (5’-CACTCGAGGCTCTGTCGGCTTCA-3’) and antisense 2 (5’-CAGCGGCCGCTCTTTAGTCATCTTTTGGCTT-3’). The two *CAST* fragments were digested with SalI and XhoI, and XhoI and NotI, respectively, cloned into the SalI and NotI sites of pCI vector, and then confirmed by DNA sequencing. Plasmids pCI-h*IRAK-M*-His, pCI-h*IRAK-M*-ΔCTD-His, pCI-h*TRAF6*-His, and pCI-h*CAST*-His were also generated starting from PCR amplification. The primers are listed in Supplementary Table 3. The PCR products were digested with SalI and NotI and ligated into the digested pCI vector. The His-tag sequence was linked to the C-terminus of full-length *IRAK-M*, *IRAK-M*-ΔCTD (a truncated *IRAK-M* variant lacking the entire CTD domain), *TRAF6*, or *CAST*. To stably knockdown IRAK-M in human melanoma cells, the lentiviral pSIH1-sihIRAK-M-copGFP vector was constructed according to instructions from System Biosciences. The sense and antisense siRNA sequences targeting human IRAK-M (5′-GATCCGGACATCGTCGAGCTATTCATTCAAGAGATGAATAGCTCGACGATGTCCTTTTTG-3′ and 5′-AATTCAAAAAGGACATCGTCGAGCTATTCATCTCTTGAATGAATAGCTCGACGATGTCCG-3′) were synthesized, phosphorylated, and annealed. Then the annealed double stranded DNA was inserted into the BamHI and EcoRI sites of the 3′ LTR of pSIH-H1-copGFP lentivector.

### Transfection, calpain activity, and apoptosis assays

Transfection was conducted by electroporation using the Amaxa Nucleofector (Lonza) according to the manufacturer’s protocol. For transfection of melanocytes, 1.5 × 10^6^ cells were washed with 1 × PBS and resuspended in the NHEM-Neo nucleofector solution. Five microgram of plasmid DNA was added to the cell suspension and thoroughly mixed. The resuspended cells were subsequently transferred into a cuvette and electroporated using program U-024, and then transferred into 6-well flat-bottom plates. A specific nucleofector solution and a specific program were applied for each melanoma cell line. 2 × 10^6^ G361 cells were suspended in the nucleofector solution L, mixed with 2 µg of plasmid DNA, and subjected to electroporation using program X-005. 2 × 10^6^ C32 cells were electroporated in solution L with 3 µg plasmid DNA using program T-020. For Malme-3M, 2 × 10^6^ cells were mixed with 5 µg plasmid DNA in solution V and program T-020 was applied. For SK-MEL-28, 1.25 × 10^6^ cells were mixed with 5 µg of plasmid DNA in solution V and electroporated using program T-020. And for RPMI7951, A101D, and HS294T, 2 × 10^6^ cells were mixed with 5 µg plasmid DNA in solution V and program X-001 was used.

Cell lysates were prepared from treated human melanoma cells and subjected to calpain activity assay by a commercial assay kit (EMD Millipore) according to the manufacturer’s instructions. All tests were performed in triplicate wells. Apoptosis was determined by flow cytometry after staining cells with PI and Annexin V. All flow cytometry was performed at the University of Maryland Greenebaum Comprehensive Cancer Center Flow Cytometry Shared Services on the BD LSR II (BD Biosciences) and FACS data were analyzed using FlowJo software (Tree Star Inc).

### Lentivirus production and transduction of melanoma cells

Recombinant lentiviruses were produced by transiently transfecting 293 T/17 cells with plasmid DNA by the Lipofectamine 2000 transfection reagent (Invitrogen) using pPACKH1 HIV Lentivector Packaging Kit (System Biosciences). 48–60 h later, supernatants were collected and filtered through Millex-HP 0.45 μm filter (EMD Millipore). C32 and Malme-3M cells were transduced with lentiviruses containing pSIH-H1-copGFP or pSIH1-sihIRAK-M-copGFP for 24 h at 10–20 viral particles per cell in the presence of 8 µg/ml polybrene, replaced with complete medium. GFP-positive cells were sorted using FACS.

### Immunoprecipitation (IP)

Eighteen hours after melanoma cells were transfected with plasmids, cells were collected and lysed with ice-cold 1 × cell lysis buffer (Cell Signaling Technology) containing protease and phosphatase inhibitor cocktail (Thermo Scientific). Supernatants were collected after centrifugation at 14,000 × *g* for 20 min at 4 °C. Two hundred microliter of cell lysates were mixed with 10 µl of sepharose bead conjugated His-tag mouse antibody (Cell Signaling Technology), incubated overnight at 4 °C, and then washed five times with 1× cell lysis buffer. Laemmli sample buffer was added to washed beads and boiled for 5 min at 95 °C. Samples were furtherly subjected to western blot analysis.

### Drug screening, drug-induced gene expression, and apoptosis

The epigenetics compound library (L1900) containing 128 inhibitors of various epigenetic enzymes was purchased from Selleck Chemicals. These epigenetic compounds inhibit the activity of epigenetic modifying enzymes such as histone deacetylase (HDAC), DNA methyltransferase, histone methyltransferase, or histone demethylase. 2.5 × 10^4^ human melanoma cells (G361, C32, Malme-3M, or SK-MEL-28) were cultured with chemical inhibitors at four different concentrations (0.1, 1, 2.5, and 10 µM) in 50 µl of culture media without phenol red in 96-well black flat-bottom plates. Forty-eight hours after treatment, cell cytotoxicity induced by epigenetic compounds was measured using the CellTox green cytotoxicity assay (Promega). Hundred microliter of 1:500 diluted CellTox green dye in assay buffer was added to each well and incubated for 15 min at room temperature. Fluorescence intensity was measured at 485nmEx*/*520nmEm by a multi-detection microplate reader. The raw cytotoxicity values were normalized to DMSO controls, which were set to 1.

The epigenetic compounds with at least a 1.5-fold increase were selected and further subjected to IRAK-M induction and apoptosis assays in G361, C32, Malme-3M, and SK-MEL-28 cell lines. 5 × 10^5^ melanoma cells were treated with selected inhibitors in 6-well flat-bottom plates containing 3 ml of culture medium for 72 h, and then collected for the analysis of IRAK-M expression by western blot and apoptosis assay using FACS. Furthermore, 5 × 10^5^ human normal melanocytes (Mel-neo and HEMa-LP) were treated with EPZ-6438, 5-aza, or I-BET762 at various concentrations (5, 10, 25, and 50 µM) for 72 h in 3 ml of culture medium in 6-well plates and analyzed by Western blot and FACS.

### Caspase and calpain inhibitor studies

For caspase inhibitor treatments, melanoma cells (2 × 10^6^/well) were pretreated with 20 µM Z-DEVD-FMK (a caspase-3 inhibitor), Z-IETD-FMK (a caspase-8 inhibitor), or Z-LEHD-FMK (a caspase-9 inhibitor) in 6-well plates for 24 h, followed by electroporation with IRAK-M vector, and then exposed to 20 µM caspase inhibitor for an additional 24 h. Cells were collected and subjected to apoptosis assays. For calpain inhibitor treatment, C32 cells (2 × 10^6^/well) were pretreated with 20 µM calpeptin (a calpain inhibitor) or DMSO in 6-well plates for 24 h, followed by electroporation with empty vector or IRAK-M construct, and then exposed to 20 µM calpeptin or DMSO for an additional 24 h. For Malme-3M, 5 × 10^5^ cells were pretreated with DMSO or calpeptin at different concentrations (20, 40, and 60 µM) for 24 h, and challenged with DMSO or 50 µM azacytidine along with calpeptin for 48 h. After treatment, cells were harvested and subjected to western blot, calpain activity, and apoptosis assays.

### In vivo xenograft studies of tumor growth

Four- to six-week-old female NSG mice (*n* = 5 for each group) were subcutaneously xenografted with 6 × 10^6^ Malme-3M tumor cells on the back. Mice were assessed every 2–3 days for palpable tumors. Once the tumors were palpable (~10 mm^2^), mice were treated with either PBS vehicle control or 2 mg/kg Azacytidine intraperitoneally for 5 consecutive days followed by 5 consecutive days of rest. These cycles continued until mice reached an endpoint of 10 mm tumor length in any dimension. Tumor area was measured with digital caliper and tumor sizes (mm^2^) were calculated by multiplying length by width of the tumor mass. Experiments were performed independently twice, with each experiment yielding similar trends. Tumor sizes (mm^2^) were analyzed using a mixed model approach for repeated measurements.

### Statistics and reproducibility

Data were processed using Microsoft Excel and GraphPad Prism 7.0 software, and ImageJ software was used to analyze Western blot. All statistical analyses are depicted in the figure legends. Results are shown as mean ± SEM. Statistical significance was assessed using either unpaired two-tailed Student’s *t*-test or two-way ANOVA unless otherwise indicated in the figure legends. *P*-values of 0.05 or less were considered statistically significance and presented in figures (*p < 0.05, **p < 0.01).

### Reporting summary

Further information on research design is available in the Nature Research Reporting Summary linked to this article.

## Supplementary information

Supplementary Information

Description of Additional Supplementary Files

Supplementary Data

Reporting Summary

## Data Availability

mRNA expression and DNA methylation profiles data for melanoma cell lines presented in Fig. 1a and Supplementary Figs. 1a, 3c are available at the CCLE (datasets were previously generated). Melanoma patient survival data in Fig. 1b are from TCGA, RNA-seq gene expression, DNA methylation, and genetic alterations data for melanoma patient samples in Fig. 1g and Supplementary Fig. 3a from http://www.cbioportal.org (datasets were previously generated). IRAK-M microarray gene expression profiles data for cancer patient samples in Figs. 1d, f and Supplementary Figs. 2c, 3b are available at NCBI’s GEO (datasets were previously generated). DNA methylation levels of IRAK-M gene in Supplementary Fig. 4 and Supplementary Table 2 is generated here and the dataset is available in the GEO database. All relevant data are available from the corresponding author on reasonable request.
