# Supplementary material for: Induction of IRAK-M in melanoma induces caspase-3 dependent apoptosis by reducing TRAF6 and calpastatin levels
Source: Commun Biol. 2020 Jun 12;3:306. doi: 10.1038/s42003-020-1033-y (PMC7293221; doi:10.1038/s42003-020-1033-y)
Supplement: Supplementary file 1 — Supplementary Information [file 42003_2020_1033_MOESM1_ESM.pdf]

Supplementary Information

Induction of IRAK-M in melanoma induces caspase-3 dependent apoptosis by reducing TRAF6 and calpastatin levels

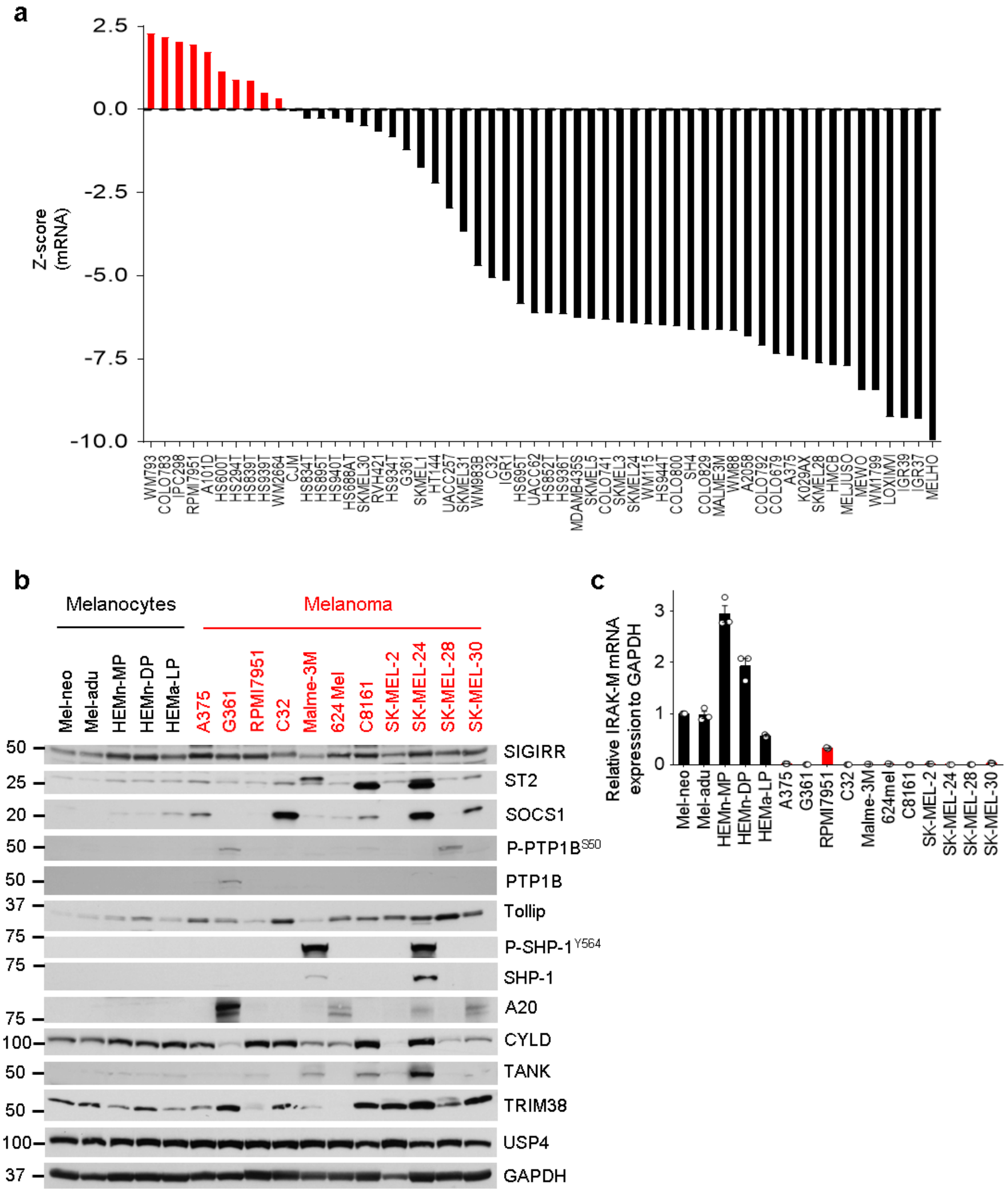
a
Z-score (mRNA)
2.5
0.0
-2.5
-5.0
-7.5
-10.0
WM793 COLO783 IPC298 RPMI7951 A101D HS600T HS294T HS839T HS939T WM2664 CJM HS834T HS895T HS940T HS688AT SKMEL30 RVH421 HS934T G361 SKMEL1 HT144 UACC257 SKMEL31 WM983B C32 IGR1 HS695T UACC62 HS852T HS936T MDAMB435S SKMEL5 COLO741 SKMEL3 SKMEL24 WM115 HS944T COLO800 SH4 COLO829 MALME3M WM88 A2058 COLO792 COLO679 A375 K029AX SKMEL28 HMCB MELJUSO MEWO WM1799 LOXIMVI IGR39 IGR37 MELHO
b
Melanocytes
Melanoma
Mel-neo Mel-adu HEMn-MP HEMn-DP HEMa-LP A375 G361 RPMI7951 C32 Malme-3M 624 Mel C8161 SK-MEL-2 SK-MEL-24 SK-MEL-28 SK-MEL-30
50 SIGIRR
25 ST2
20 SOCS1
50 P-PTP1B S50
50 PTP1B
37 Tollip
75 P-SHP-1 Y564
75 SHP-1
75 A20
100 CYLD
50 TANK
50 TRIM38
100 USP4
37 GAPDH
c
Relative IRAK-M mRNA expression to GAPDH
0
1
2
3
Mel-neo Mel-adu HEMn-MP HEMn-DP HEMa-LP A375 G361 RPMI7951 C32 Malme-3M 624mel C8161 SK-MEL-2 SK-MEL-24 SK-MEL-28 SK-MEL-30

Supplementary Figure 1. IRAK-M expression is downregulated in human melanoma. (a) Expression profile of IRAK-M mRNA in 56 human melanoma cell lines based on the CCLE database. (b) Protein expression profiles of negative regulators of TLR signaling in human melanocytes and melanoma cell lines by Western blot. (c) The mRNA level of IRAK-M was detected by real-time PCR analysis in melanocytes and melanoma cell lines (n = 3 per group). The relative level of IRAK-M mRNA in Mel-neo was set to 1. Representative data from three independent experiments are shown as mean ± SEM.

Supplementary Figure 2

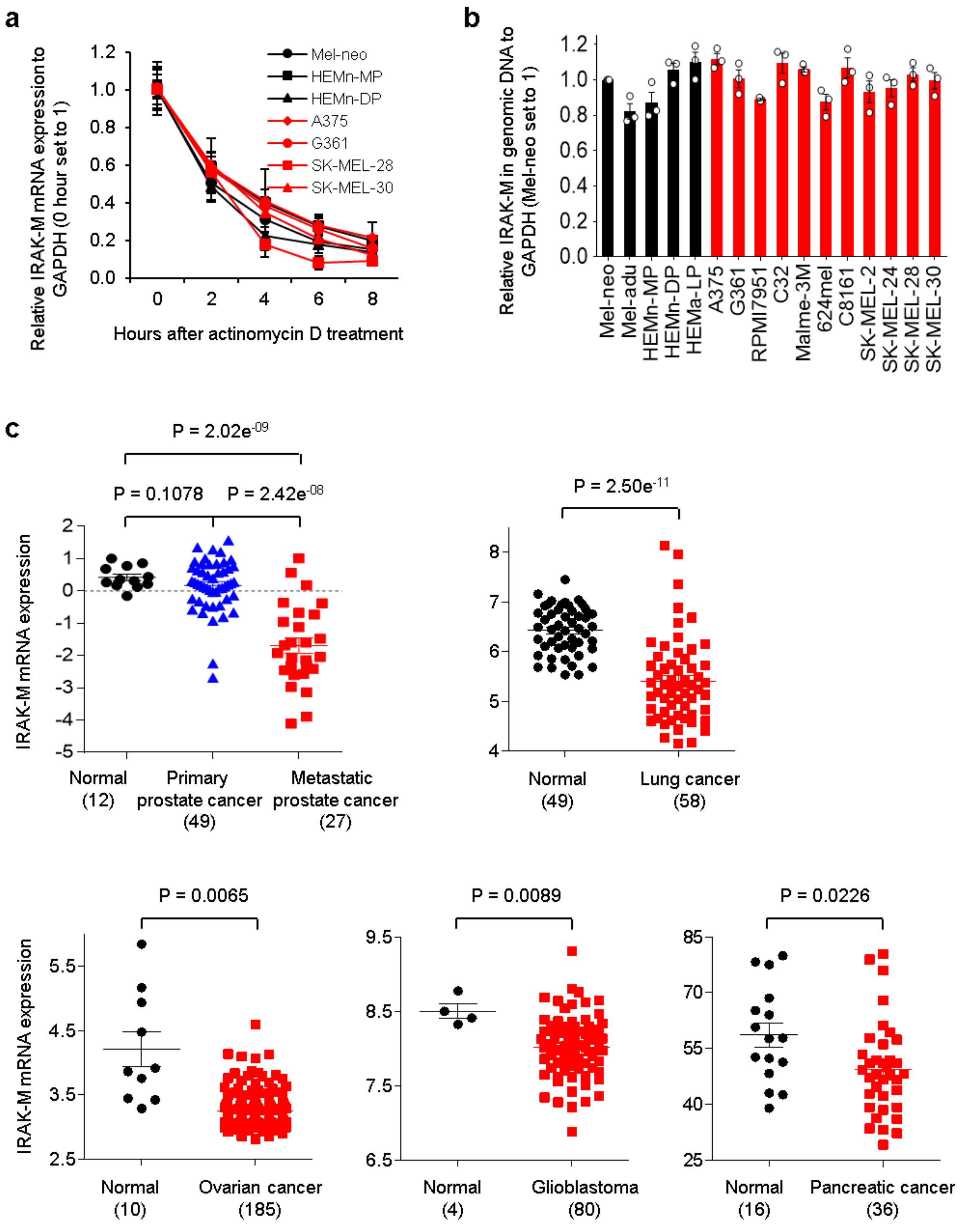

Supplementary Figure 2. IRAK-M expression is also downregulated in human other types of tumors. (a) Three melanocytes and four melanoma cell lines were treated with 100 ng/ml actinomycin D for the indicated time periods, and the relative expression levels of IRAK-M mRNA to GAPDH were analyzed by RT-PCR (n = 3 per group). The relative levels of IRAK-M mRNA in cells treated for 0 hour were set to 1. Data are shown as mean ± SEM. (b) Genomic DNA from human melanocytes and melanoma cell lines was subjected to RT-PCR (n = 3 per group). The relative copy number variation of IRAK-M was normalized to GAPDH. The relative IRAK-M in genomic DNA in Mel-neo was set to 1. Results are shown as mean ± SEM. (c) IRAK-M transcript level changes in biopsy specimens from patients with other tumor types. IRAK-M expression is obtained from the online published microarray datasets (prostate cancer, GEO GSE35988; lung cancer, GEO GSE10072; ovarian cancer, GEO GSE26712; glioblastoma, GEO GSE7696; pancreatic cancer, GEO GSE16515). The number in parentheses is the number of patients. Each symbol represents one patient. P values by two-tailed Student's t-test.

Supplementary Figure 3

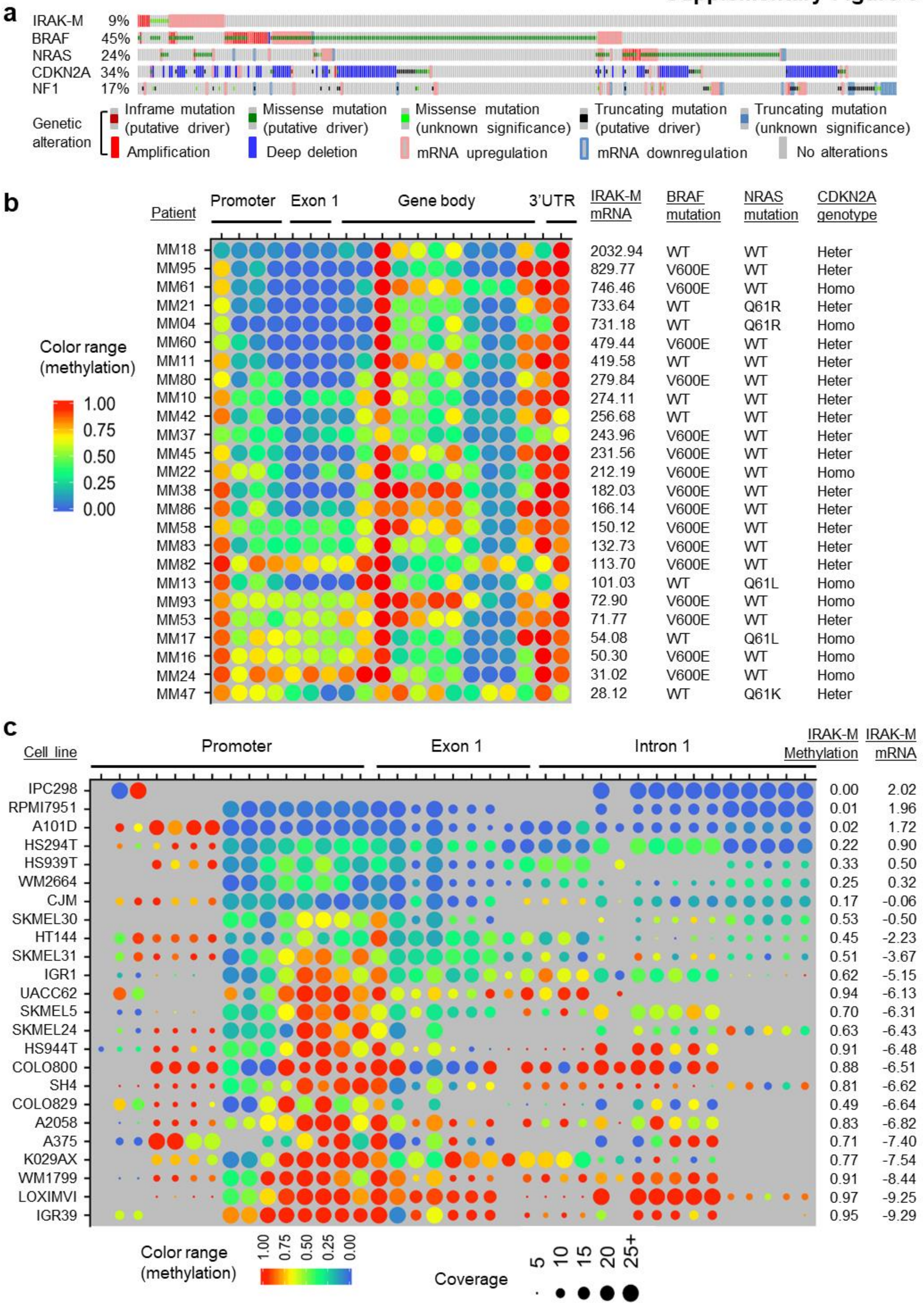

Supplementary Figure 3. IRAK-M mRNA levels correlate with DNA methylation profiles in the promoter region. (a) Genetic alterations of IRAK-M, BRAF, NRAS, CDKN2A and NF1 in 479 melanoma patient samples from the TCGA data set. (b) DNA methylation patterns and mRNA levels (signal intensity) of IRAK-M in 25 melanoma patient samples downloaded from the GEO database (GSE51547 and GSE22153). BRAF and NRAS mutation status and CDKN2A genotype in each patient sample are shown here. (c) IRAK-M DNA methylation and mRNA levels in 24 human melanoma cell lines based on data generated by the CCLE. IRAK-M methylation values (β-values) and transcript levels (Z-scores) are listed to the right of each cell line.

Supplementary Figure 4

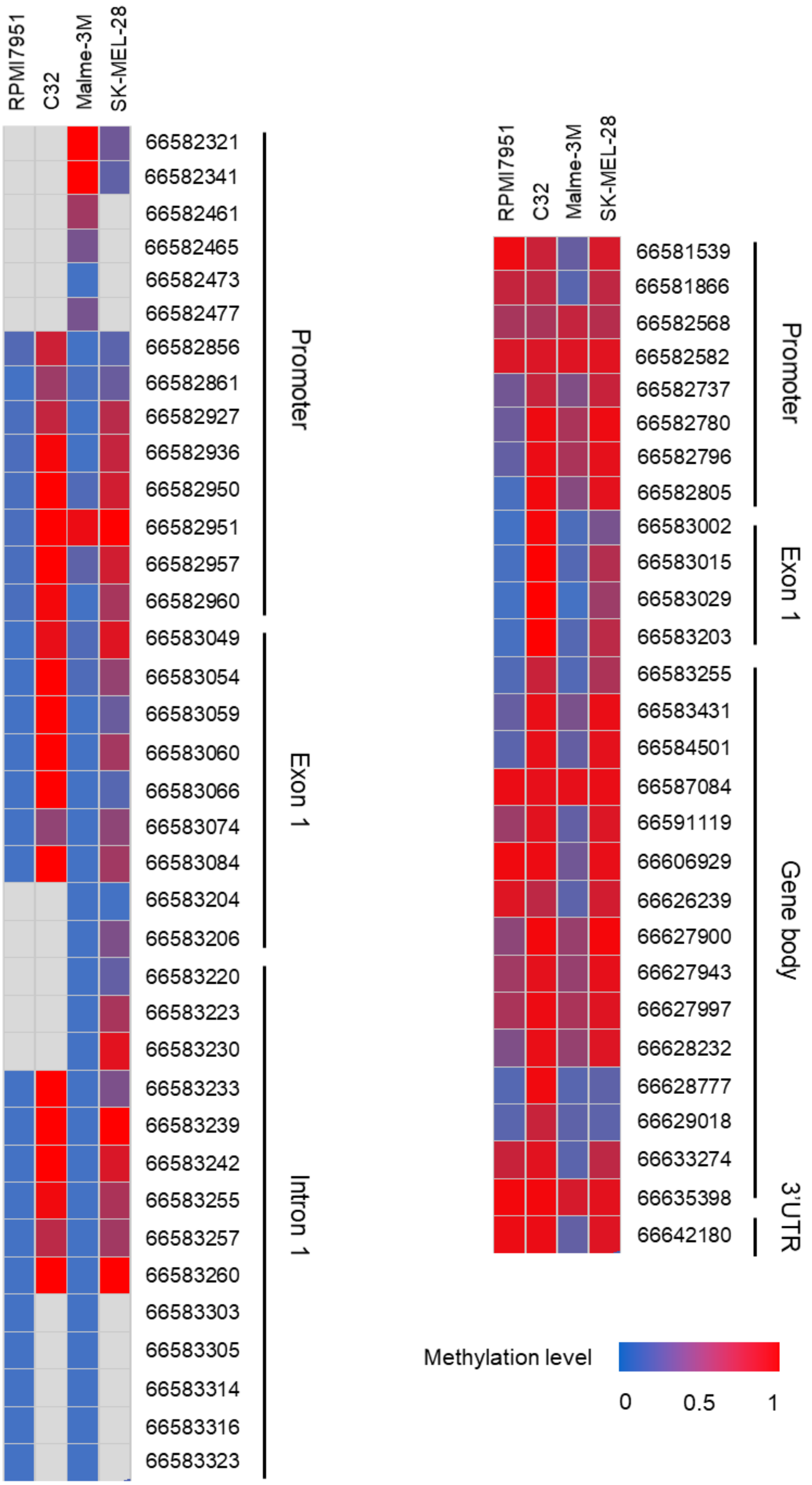

Supplementary Figure 4. DNA methylation levels of IRAK-M gene in 4 human melanoma cell lines. Left panel shows IRAK-M DNA methylation status in RPMI7951, C32, Malme-3M, and SK-MEL-28 cells based on data obtained from the CCLE. Right panel shows DNA methylation profiles of IRAK-M gene in 4 melanoma cell lines analyzed by an Infinium MethylationEPIC BeadChip array. Heatmaps are presented based on IRAK-M methylation levels (β values) and genomic positions are listed to the right of each heatmap. Light gray means data not available.

Supplementary Figure 5

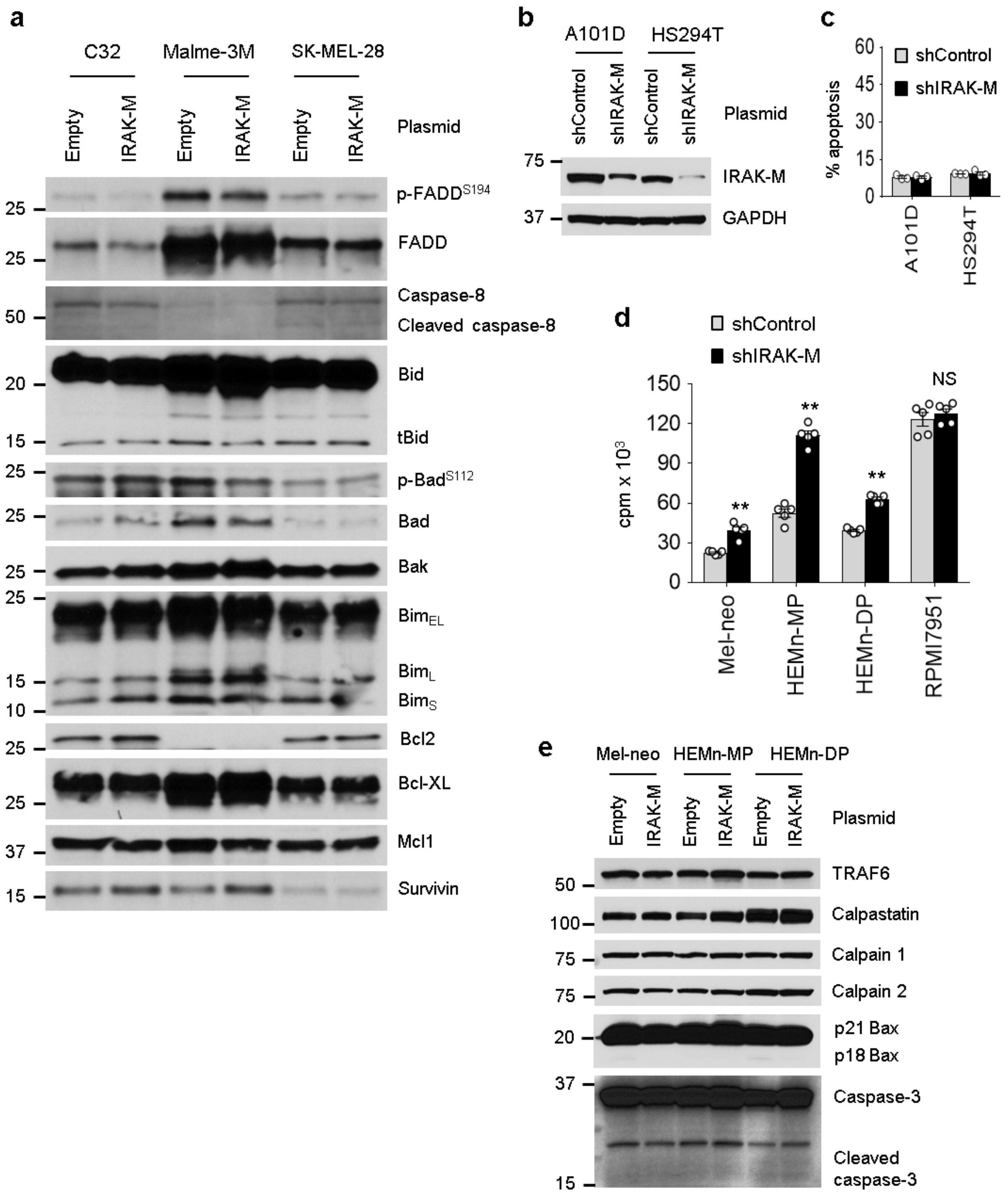

Supplementary Figure 5. Expression levels of apoptosis-related proteins in transfected melanocytes and melanoma cell lines. (a) Expression levels of proteins associated with apoptosis pathways were determined by Western blot in human melanoma cell lines transiently transfected with empty vector or IRAK-M construct for 24 hours. (b) and (c) A101D and HS294T cells were transfected with psiRNA-Control or psiRNA-hIRAK-M vector for 24 hours, followed by (b) Western blot and (c) flow cytometric analysis of apoptosis (n = 3 per group). Results are presented as mean ± SEM. Data shown are representative of two independent experiments. (d) Human epidermal melanocytes and melanoma cell line RPMI7951 were transfected with hIRAK-M kockdown vector, and cell proliferation assay was performed 24 hours later (n = 5 per group). 1×10^4 transfected cells were cultured in 200 µl culture medium per well in a 96-well round-bottom plate for 48 hours. Sixteen hours before harvesting, 0.5 µCi of ^{3}H-thymidine was added to each well prior to measuring thymidine uptake using a 1450 LSC & luminescence counter (PerkinElmer). Results are presented as mean ± SEM. **$p < 0.01$ by two-tailed Student's t-test. Data shown are representative of two independent experiments. (e) Expression levels of apoptosis-related proteins in melanocytes transfected with IRAK-M plasmid for 24 hours were evaluated by Western blot. Blots shown are representative of two independent experiments.

Supplementary Figure 6

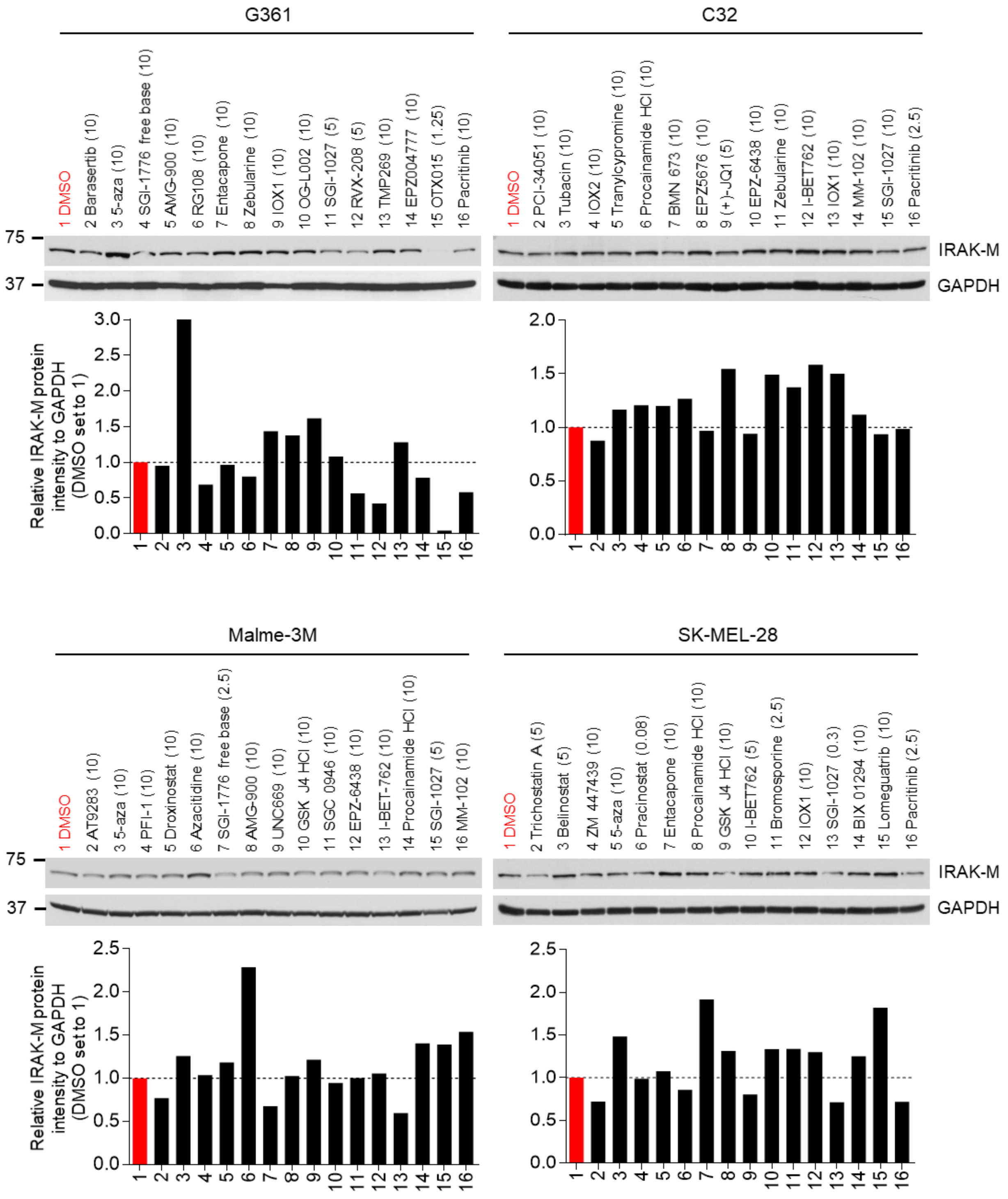

Supplementary Figure 6. Drug screening identifies compounds that induce IRAK-M expression in melanoma cell lines. Expression level of IRAK-M protein was determined by Western blot in human melanoma cells treated with the indicated inhibitors at the indicated concentrations (μM) for 72 hours. Lower panels show densitometric intensity-based ratios of IRAK-M protein to GAPDH protein. All values were normalized to DMSO value, which was set to 1.

Supplementary Figure 7

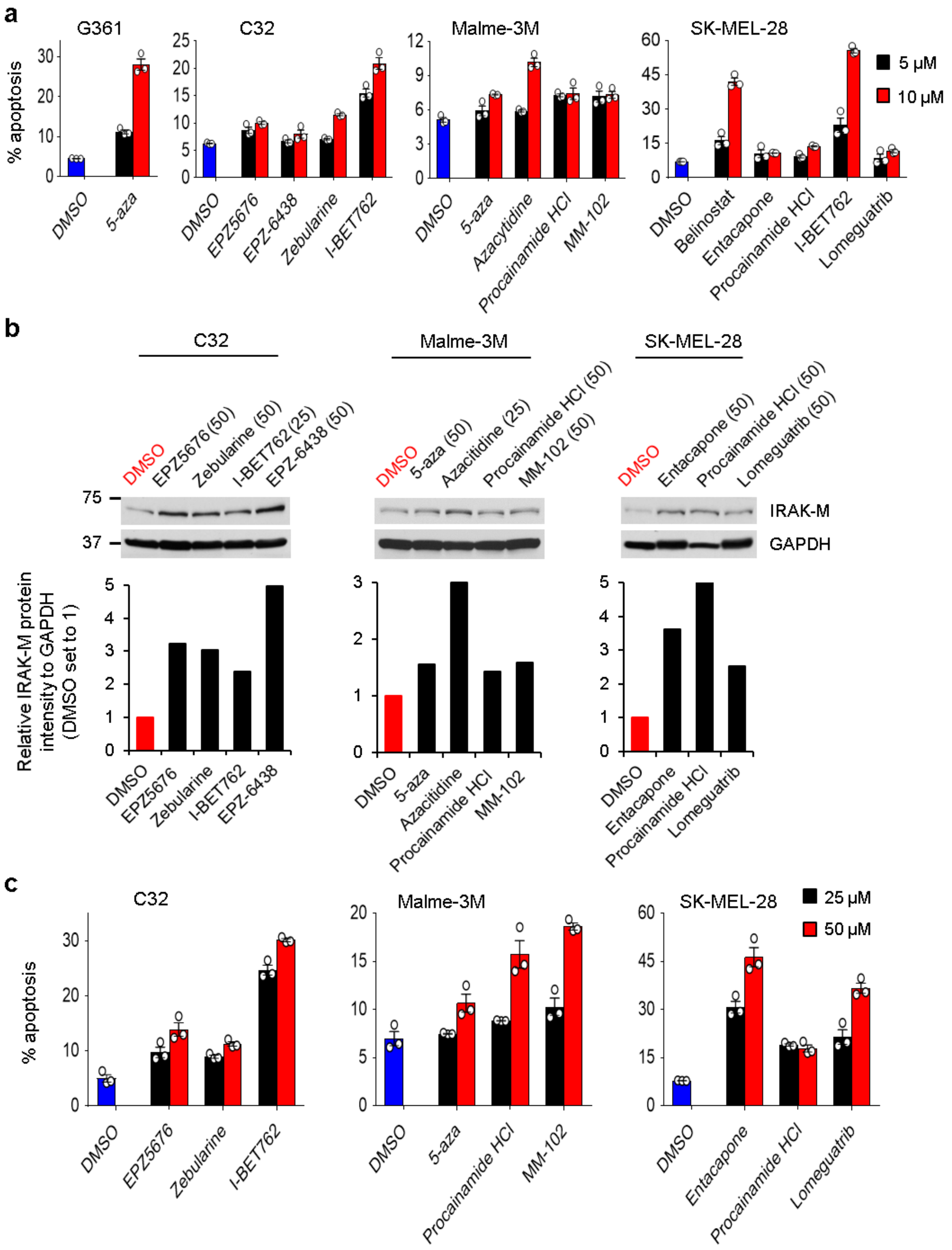

Supplementary Figure 7. Apoptosis of melanoma cells induced by epigenetics inhibitors. (a) 72 hours after four melanoma cell lines were treated with 5 or 10 µM indicated inhibitors, apoptosis was determined by staining cells with PI and Annexin V and analyzed by flow cytometry (n = 3 per group). Data are presented as mean ±SEM. (b) Expression level of IRAK-M protein was measured by Western blot 72 hours after melanoma cells were cultured with 25 or 50 µM indicated inhibitors. Lower panels show densitometric values for IRAK-M protein relative to GAPDH. All values were normalized to DMSO value, which was set to 1. (c) Three melanoma cell lines were treated with 25 or 50 µM indicated inhibitors. 72 hours later, inhibitor-induced apoptosis was evaluated by staining cells with PI and Annexin V, followed by flow cytometry (n = 3 per group). Data are shown as mean ±SEM.

Supplementary Figure 8

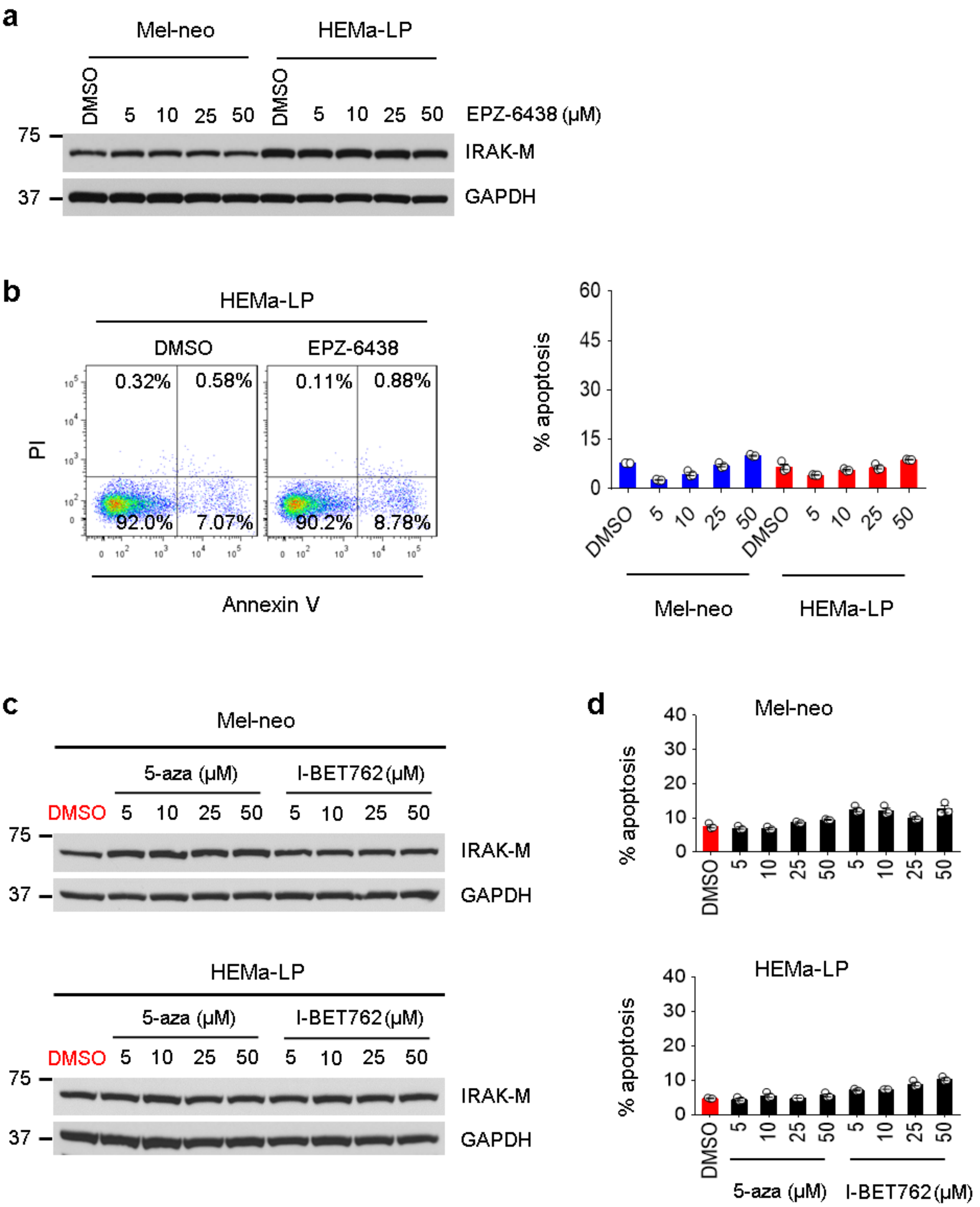

Supplementary Figure 8. IRAK-M expression and apoptosis are not induced by epigenetics inhibitors in human melanocytes. (a) and (b) Mel-neo and HEMa-LP melanocytes were treated with EPZ-6438 at the indicated concentrations (μM) for 72 hours. Expression level of IRAK-M protein was then evaluated by Western blot (a), and apoptosis induced by inhibitors was detected by flow cytometry (PI and Annexin V staining) (n = 3 per group) (b). Representative FACS analysis of apoptosis of HEMa-LP melanocytes is shown in the left panel. Data are shown as mean ±SEM. (c) IRAK-M expression and (d) apoptosis (n = 3 per group) were determined by Western blot or flow cytometry 72 hours after human melanocytes were treated with 5-aza or I-BET762. All data are presented as mean ±SEM.

Supplementary Figure 9

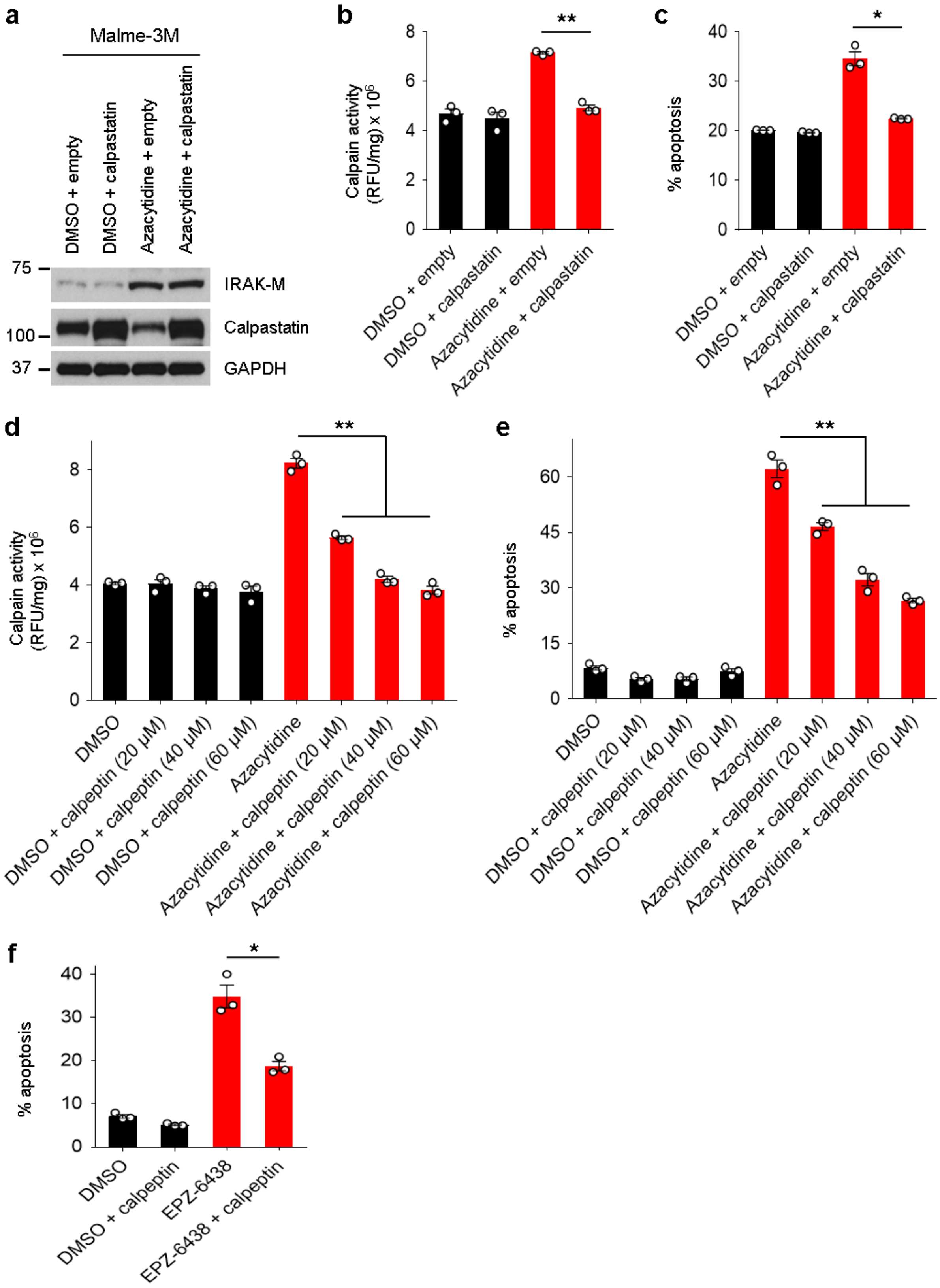

Supplementary Figure 9. Azacytidine induced apoptosis in Malme-3M cells is also mediated through calpastatin and calpain. (a-c) 24 hours after Malme-3M cells were transfected with empty vector or calpastatin construct, then cells were challenged with DMSO or 15 μM azacytidine for 48 hours. (a) Western blot was performed to detect expression levels of IRAK-M and calpastatin. (b) The fluorescence-based calpain activity assay was used to evaluate calpain activity in Malme-3M cells (n = 3 per group). And (c) apoptosis was detected by staining cells with PI and Annexin V and analyzed by flow cytometry (n = 3 per group). (d) and (e) Malme-3M cells were cultured in the presence of DMSO or calpeptin at the indicated concentrations for 24 hours, followed by treatment with 50 μM azacytidine for 48 hours. (d) Calpain activity assay (n = 3 per group) and (e) apoptosis analysis by flow cytometry (n = 3 per group) were performed. (f) C32 cells were cultured with or without 20 μM calpeptin for 24 hours, followed by treatment with 50 μM EPZ-6438 for 48 hours. Apoptosis was determined by flow cytometry (n = 3 per group). All data shown are representative of at least two independent experiments. All results are presented as mean ± SEM. In (b)–(f): $*p < 0.05$, $**p < 0.01$, two-tailed Student's t-test.

Supplementary Figure 10. Full Western blot images

Figure 1C

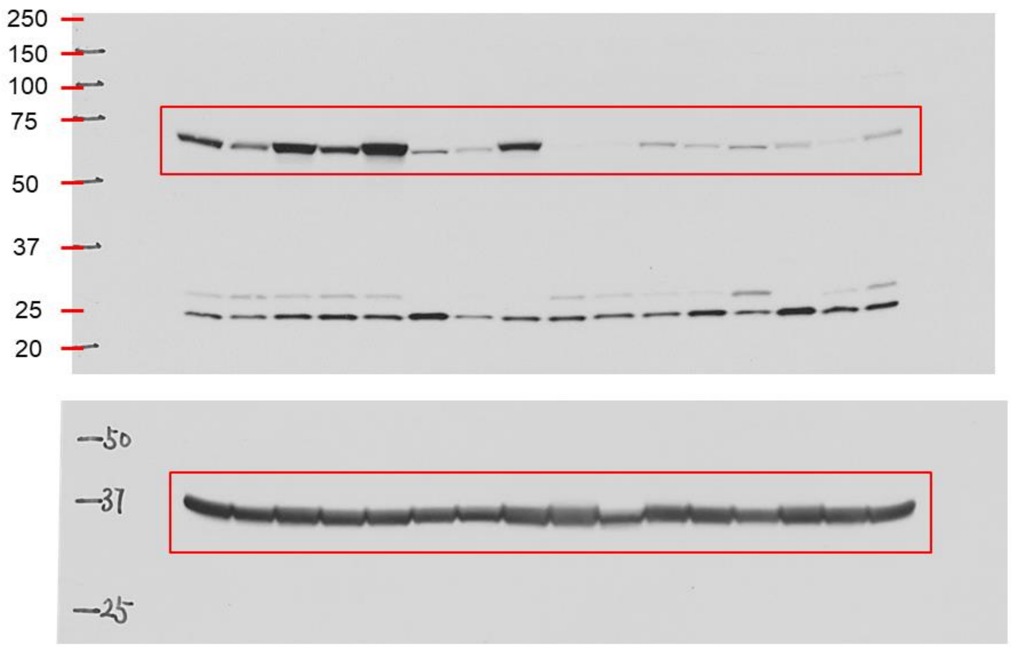

Figure 2A

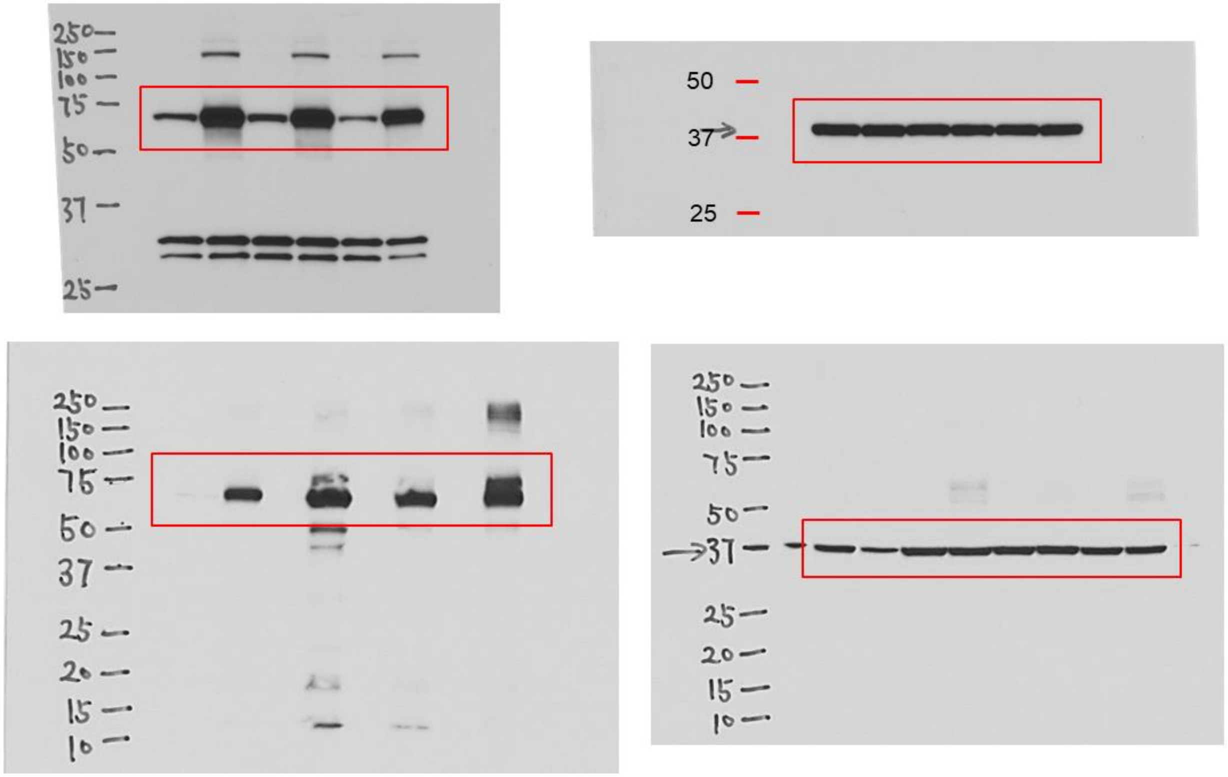

Figure 2C

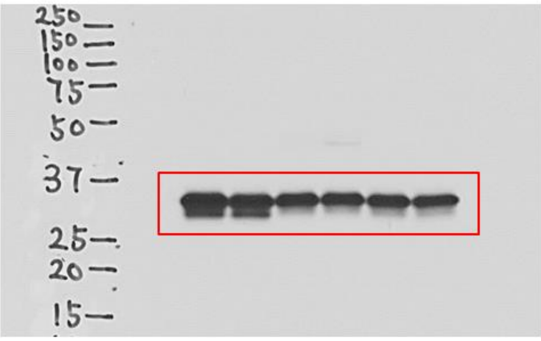

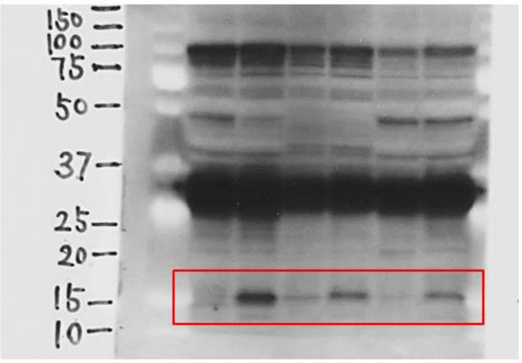

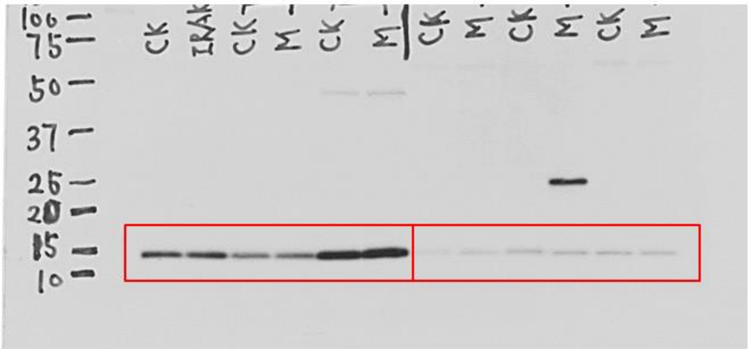

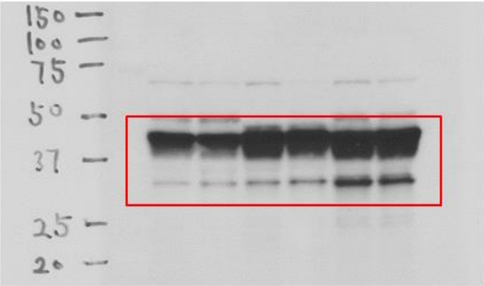

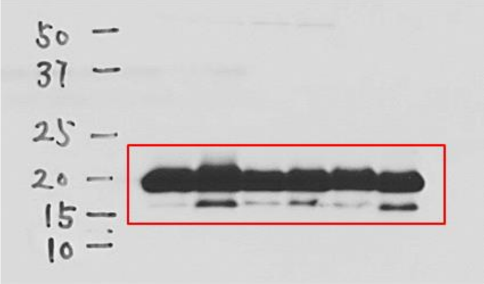

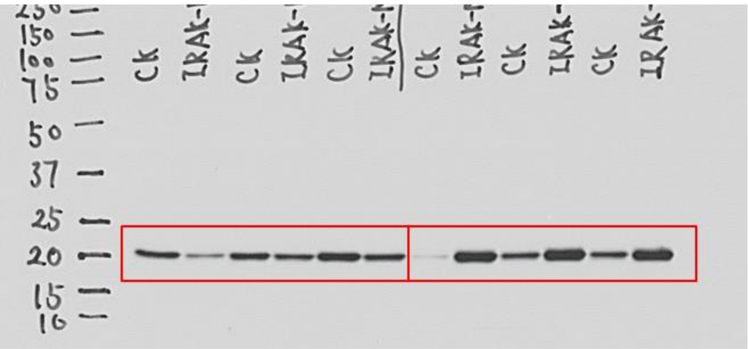

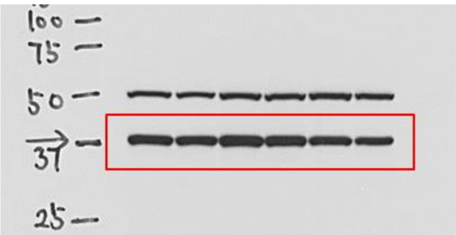

Figure 2E

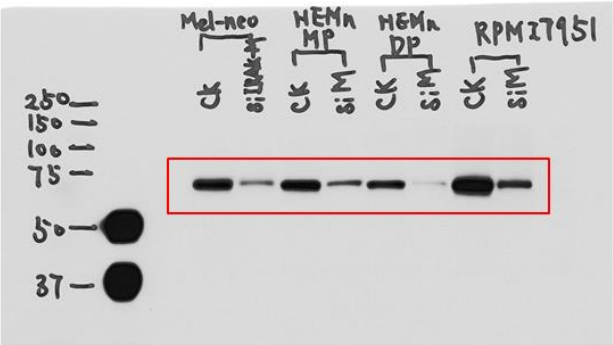

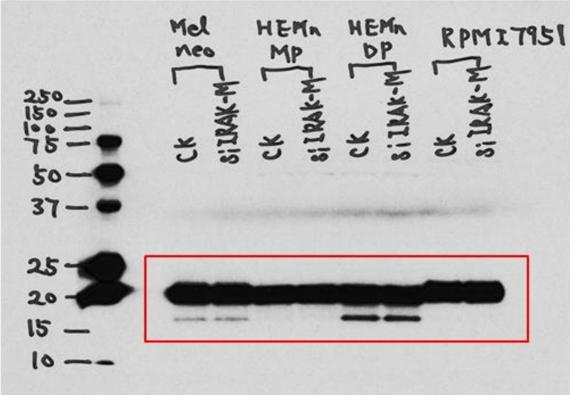

Figure 2E

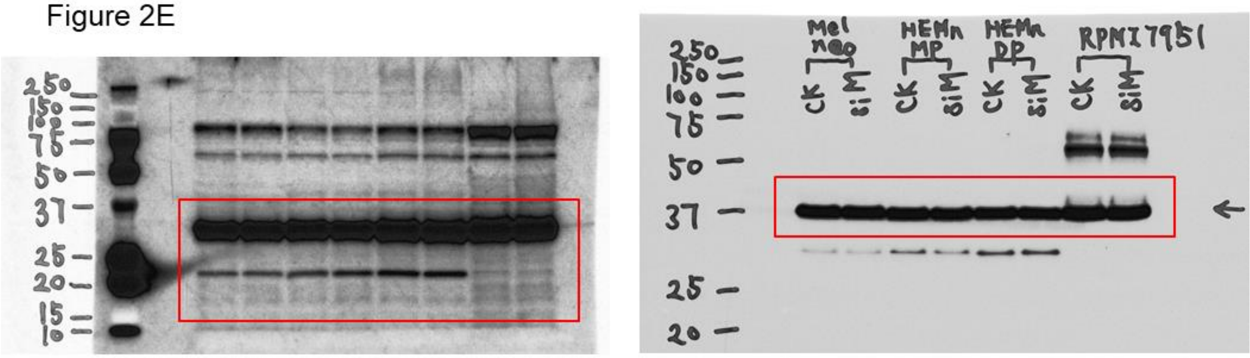

Figure 3A

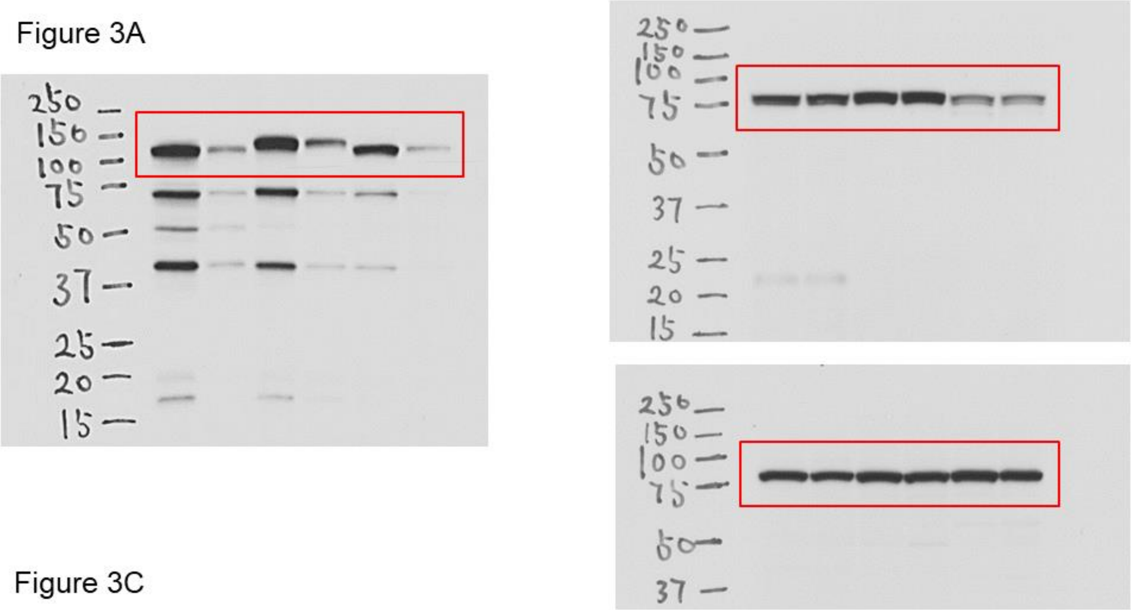

Figure 3C

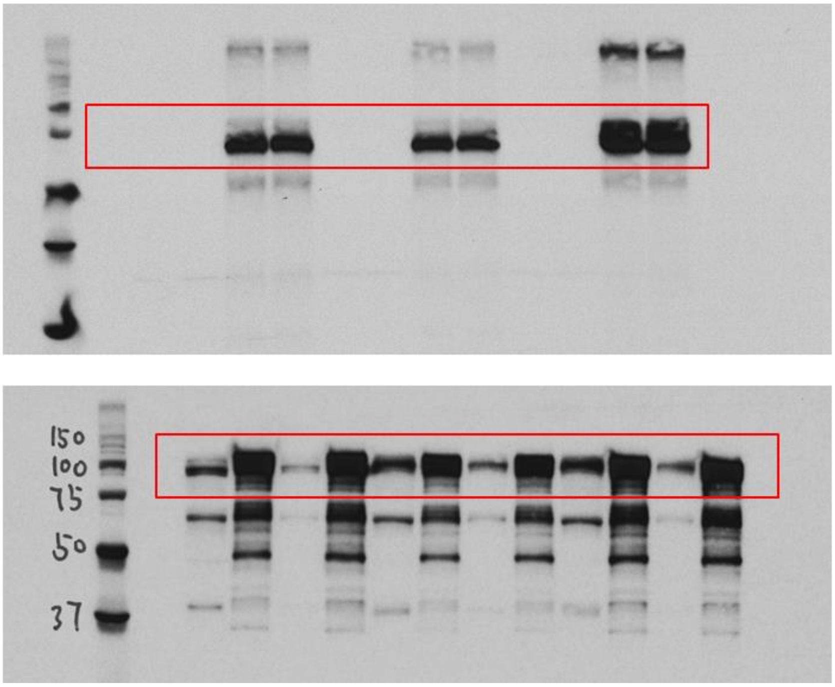

Figure 3C

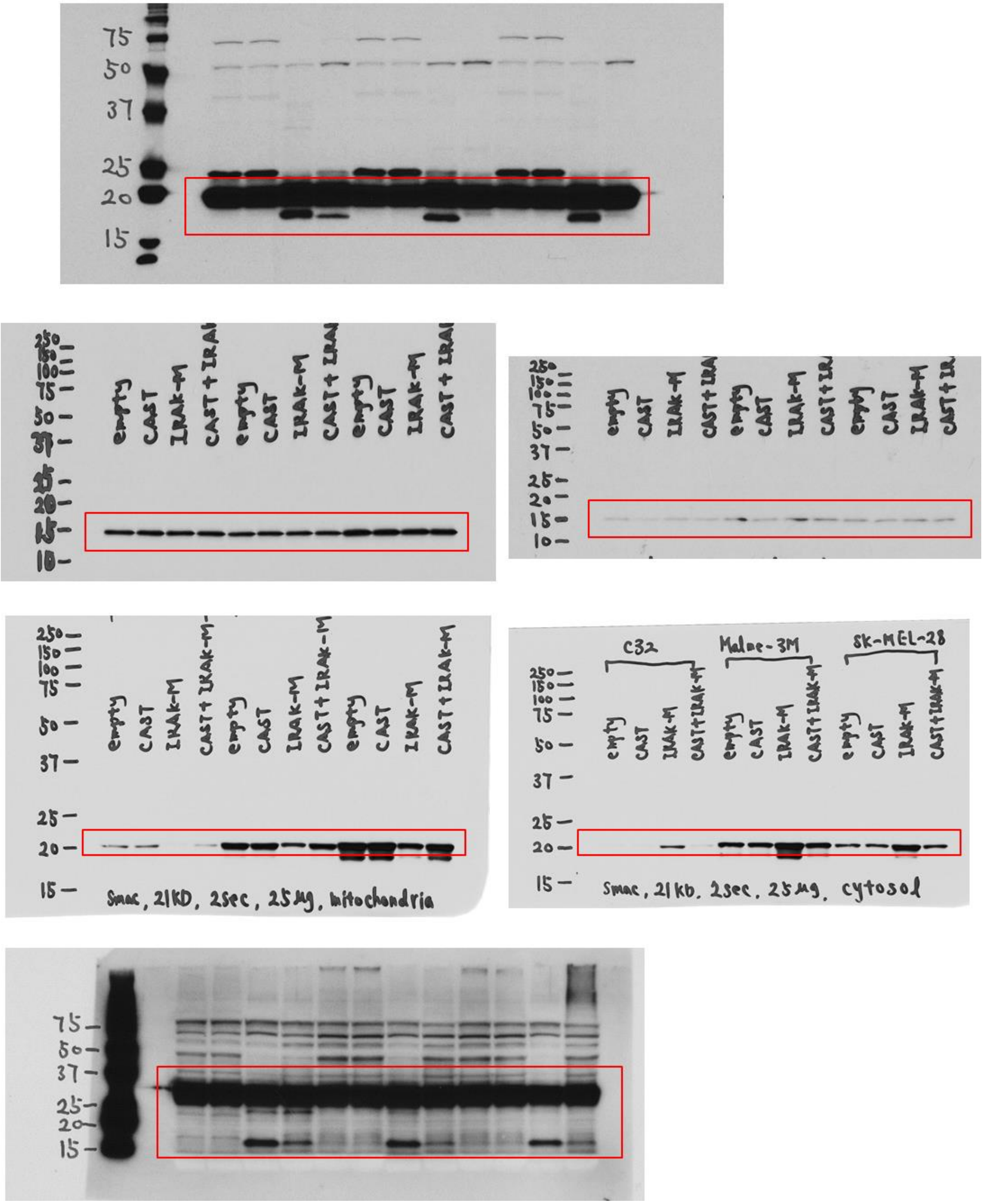
75
50
37
25
20
15
empty
CAST
IRAK-M
CAST + IRAK-M
C32
Malme-3M
SK-MEL-28
250
150
100
10
Smac, 21KD, 2sec, 25μg, mitochondria
Smac, 21kD, 2sec, 25μg, cytosol

Figure 3C

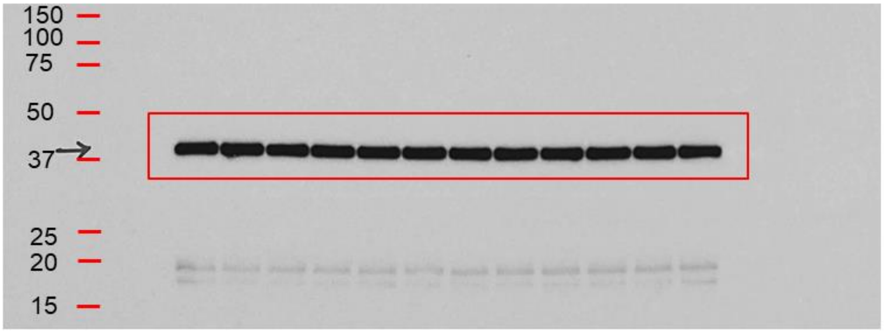

Figure 3F

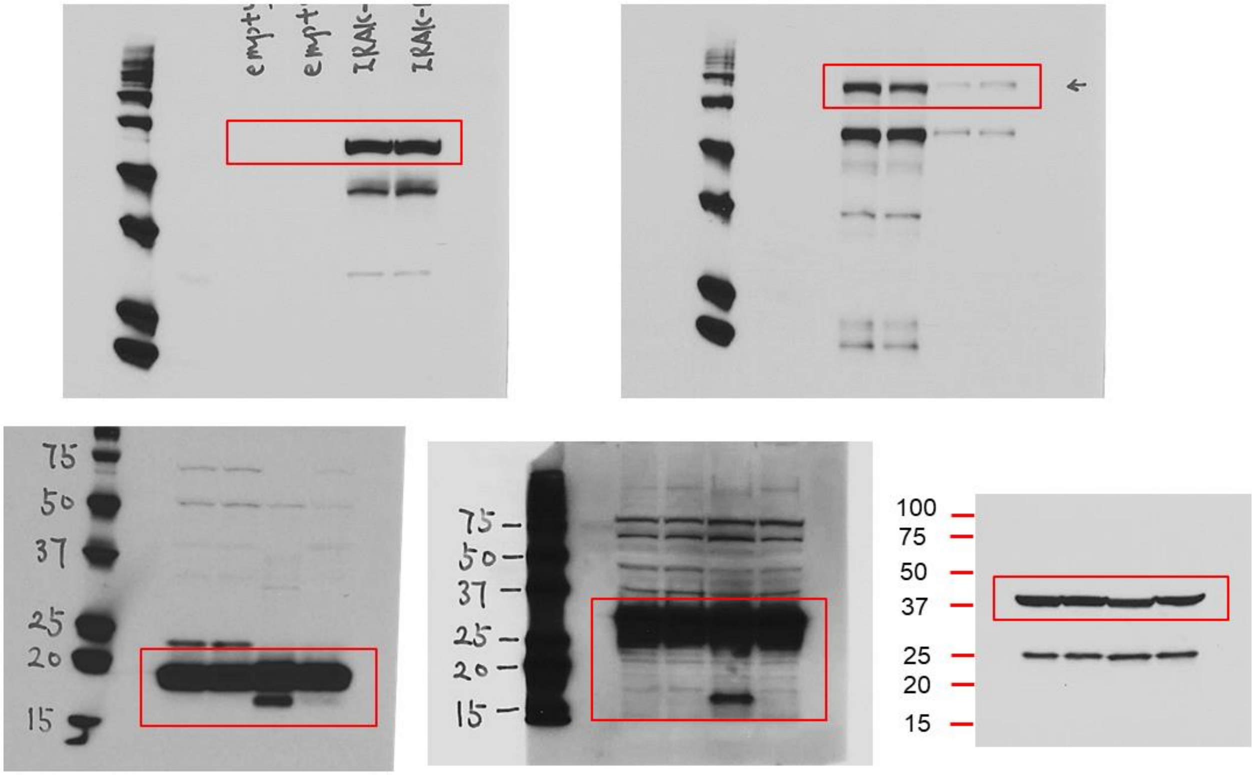

Figure 4A

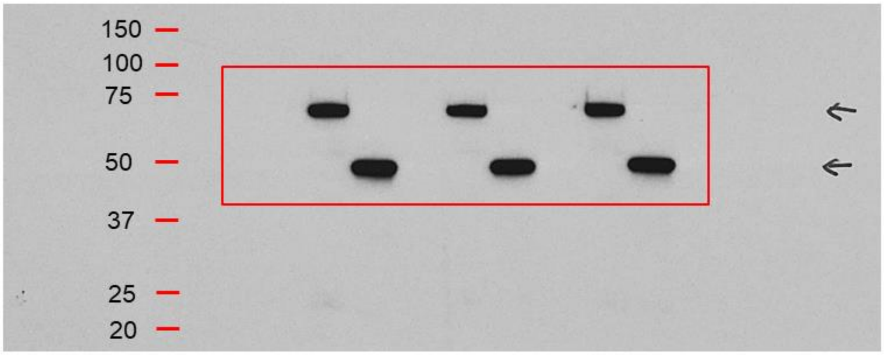

Figure 4A

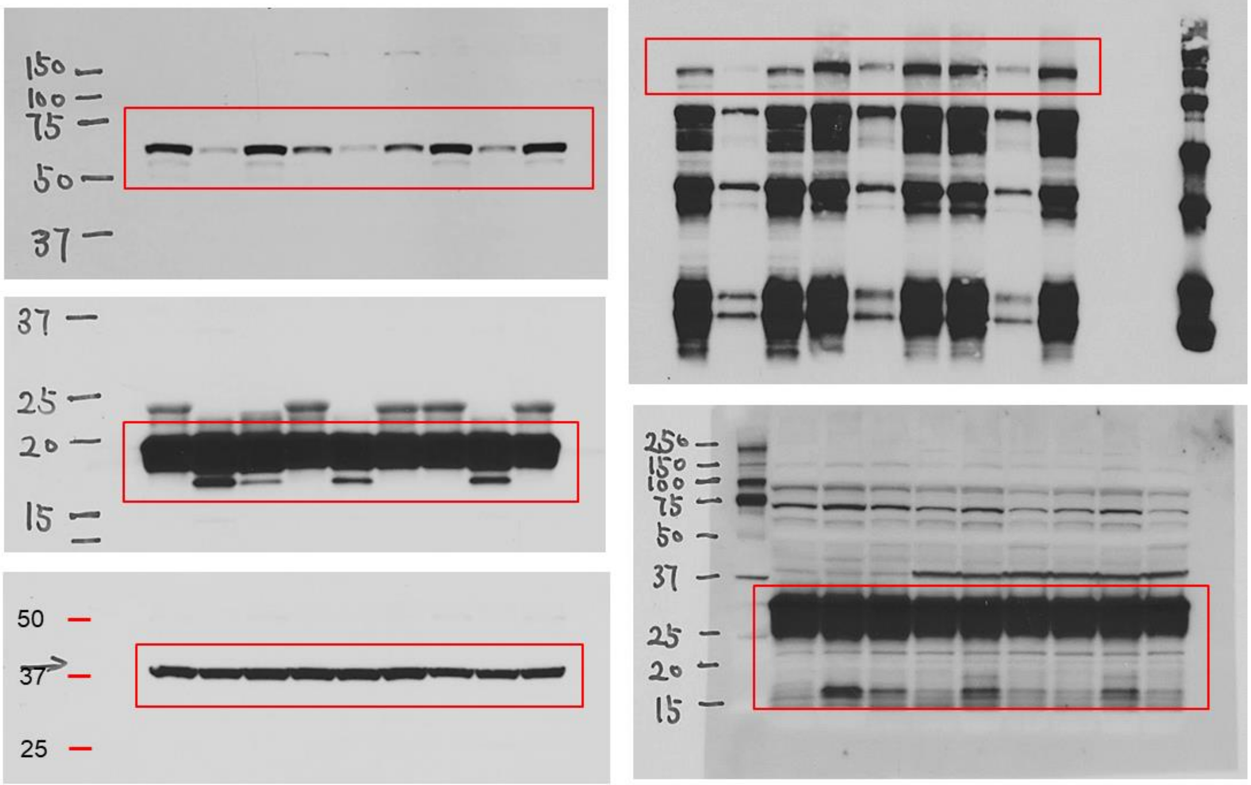

Figure 4D

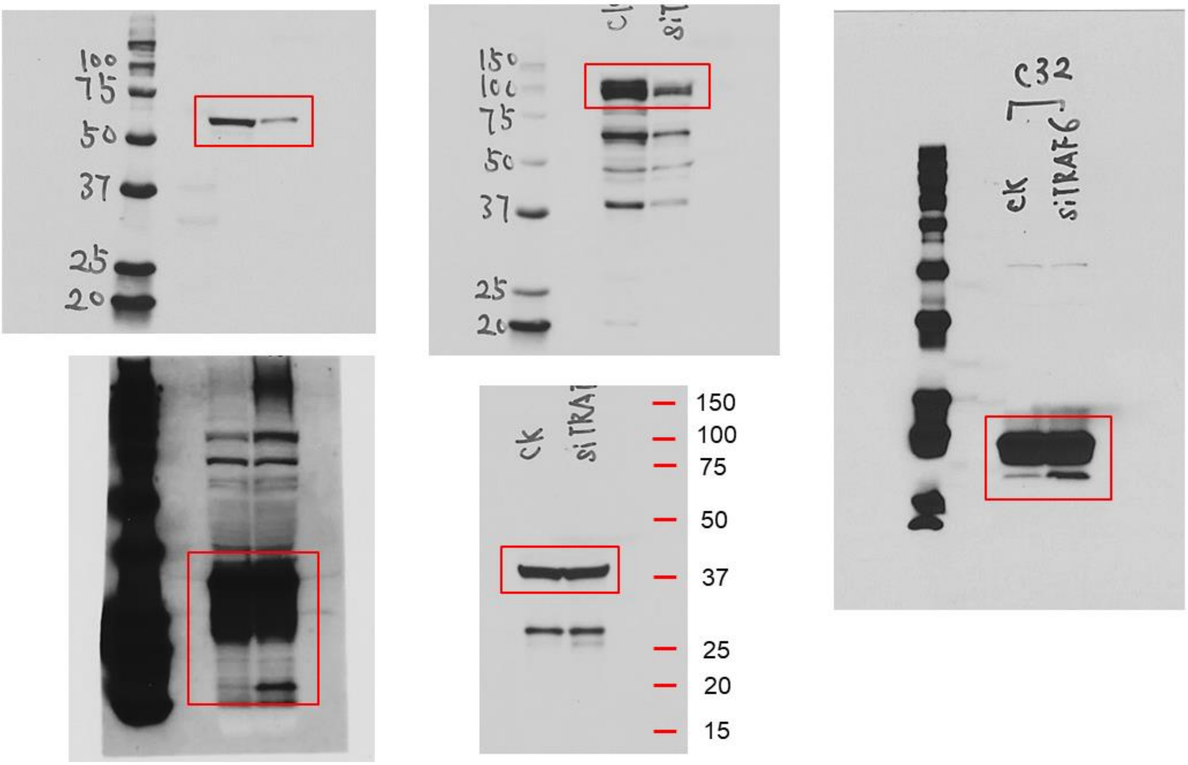

Figure 4G

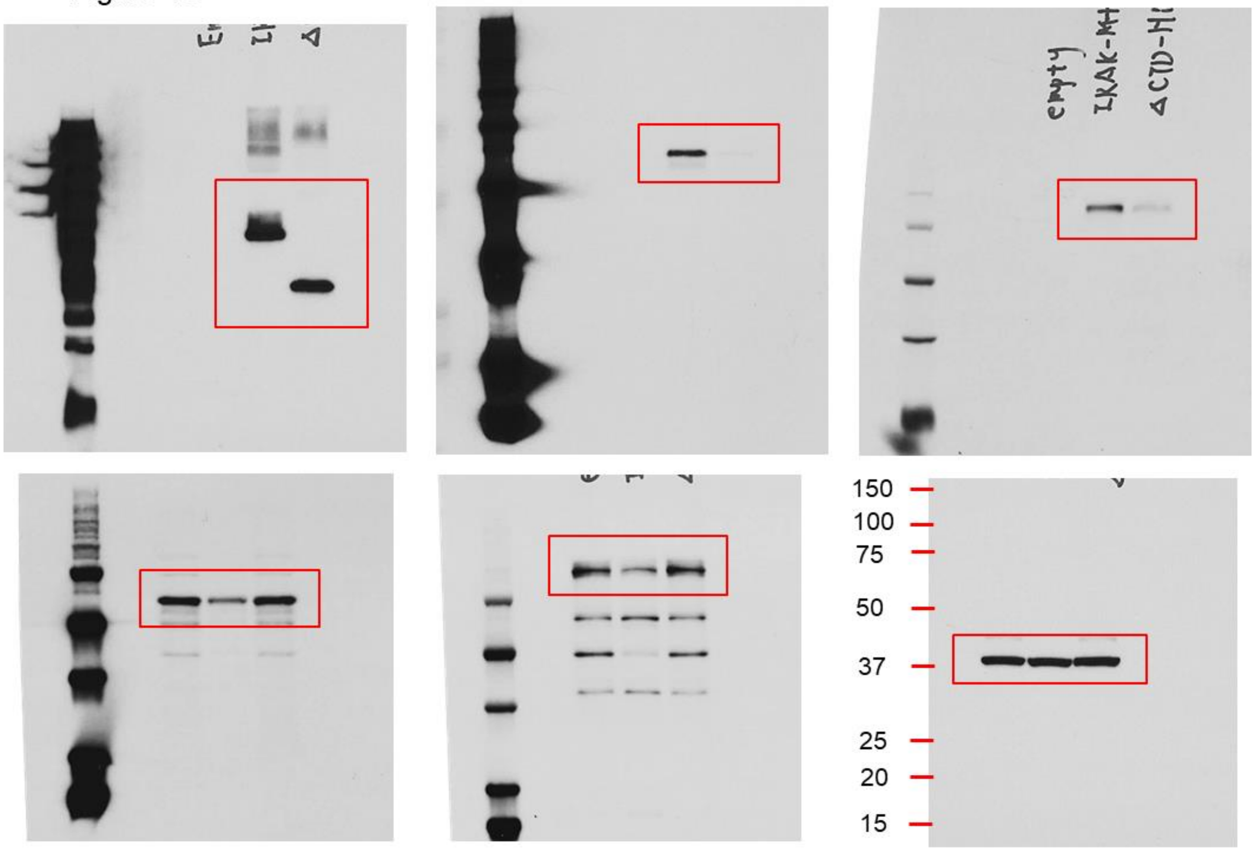

Figure 4H

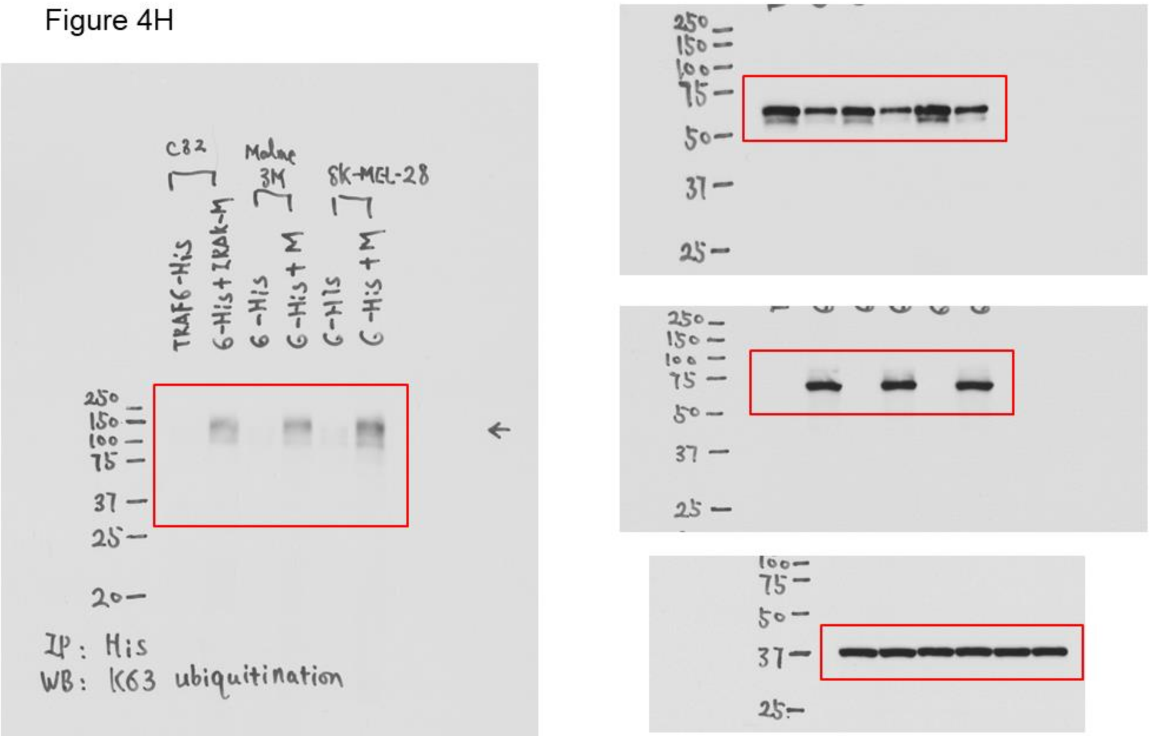

Figure 4I

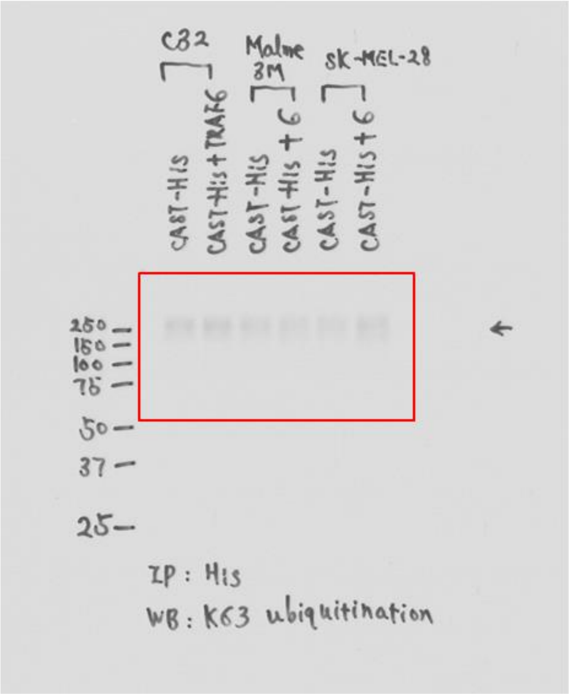

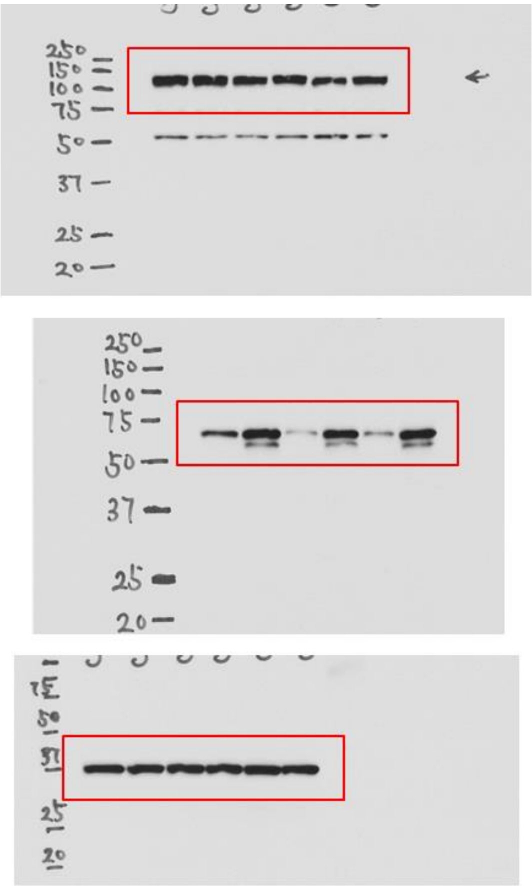

Figure 4J

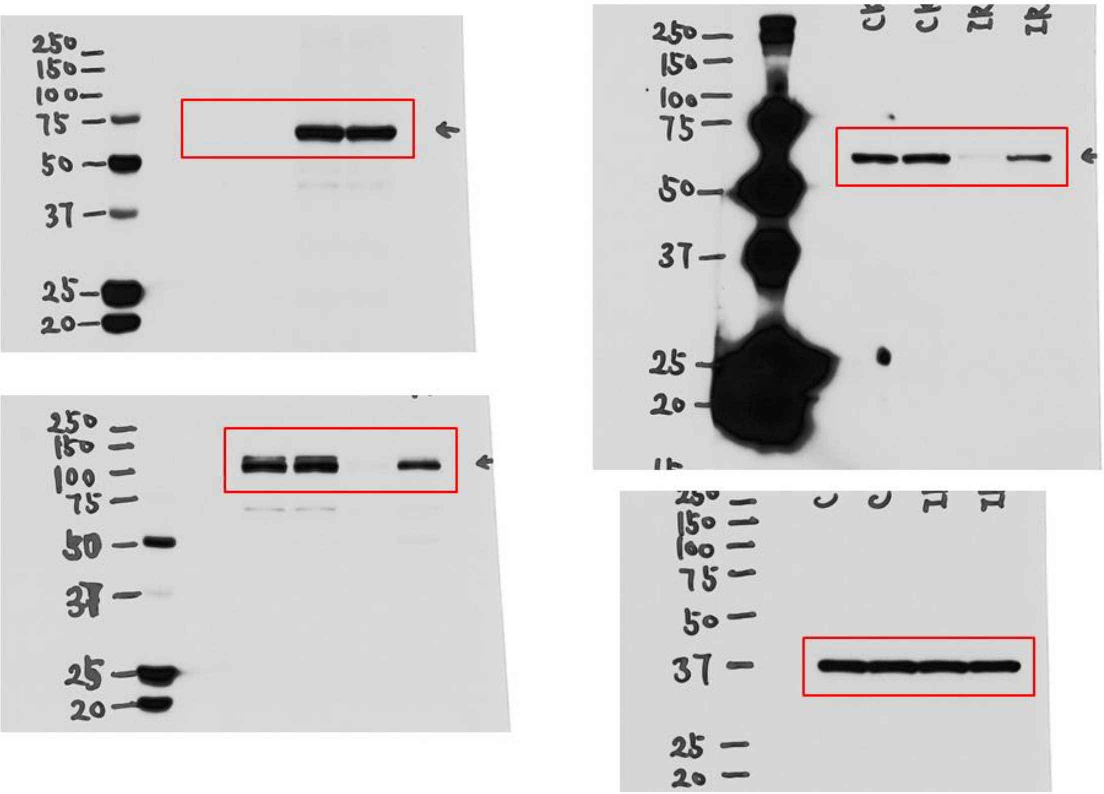

Figure 5B

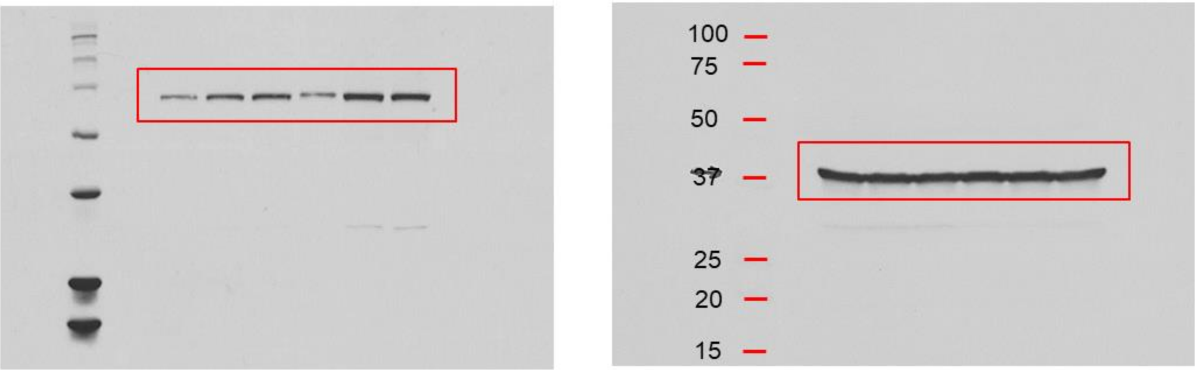

Figure 5E

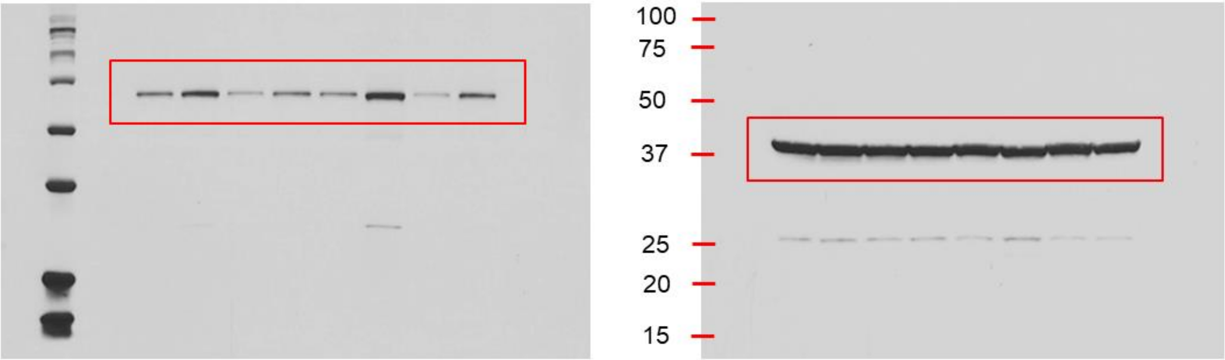

Figure 6B

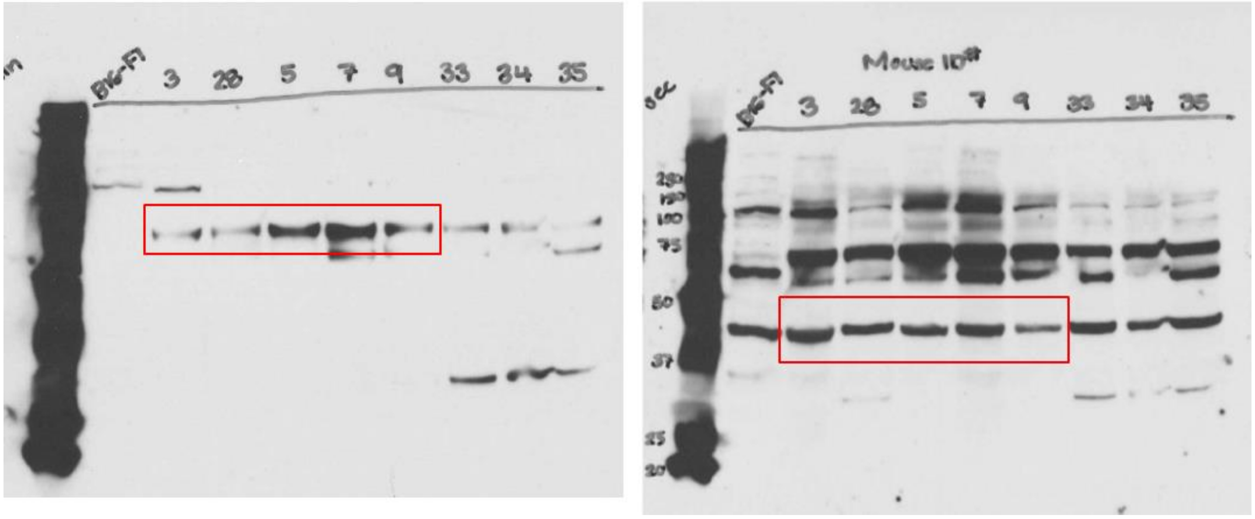

Supplementary Figure S1B

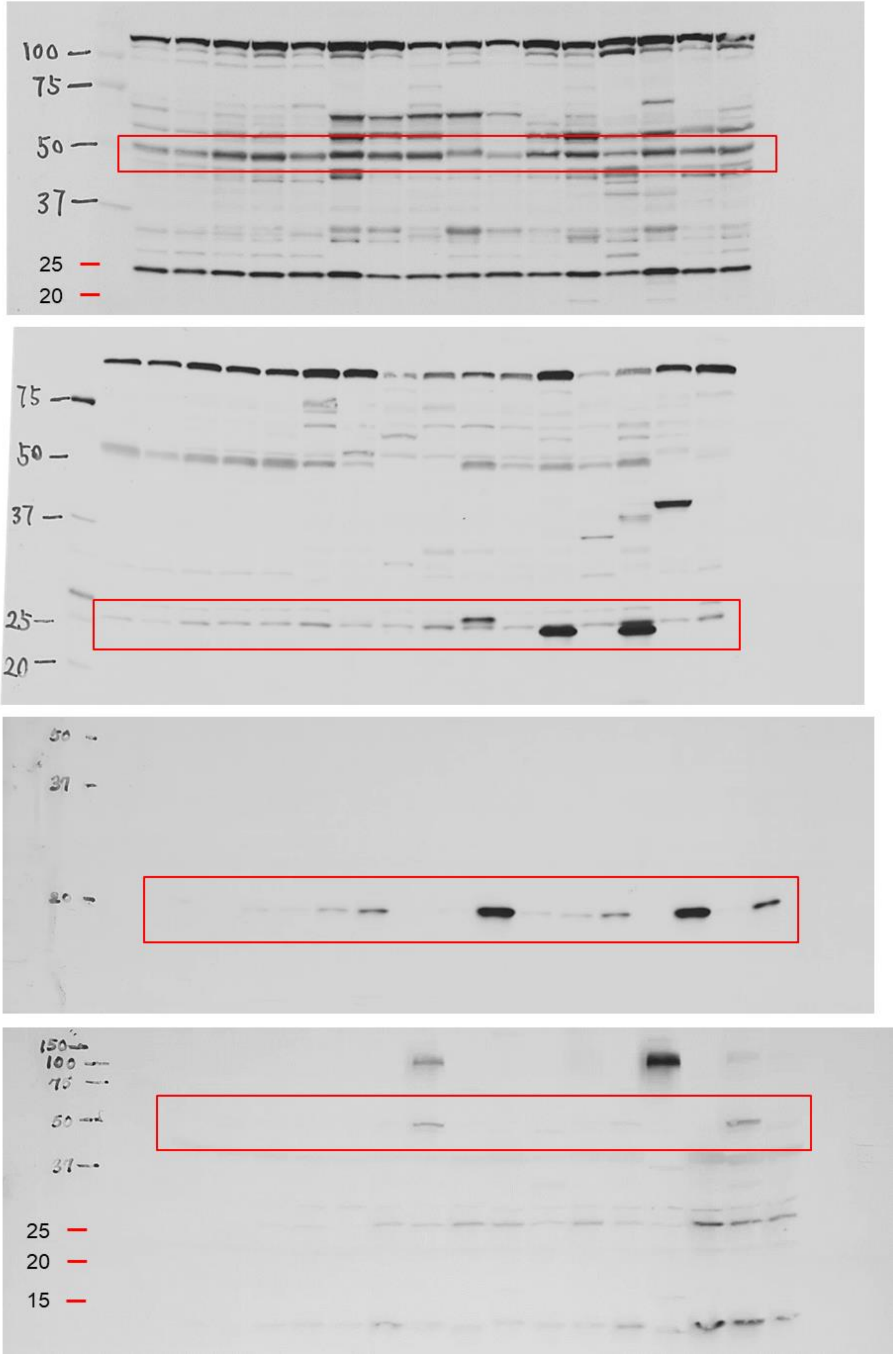

Supplementary Figure S1B

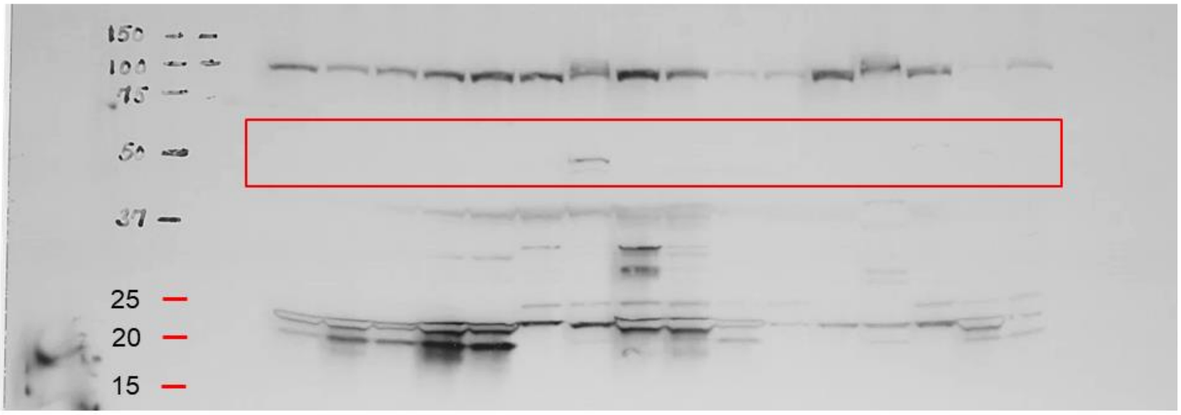

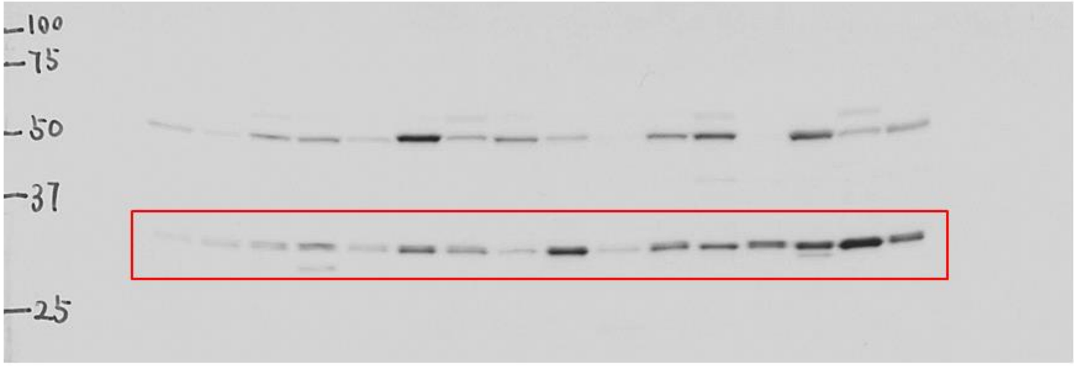

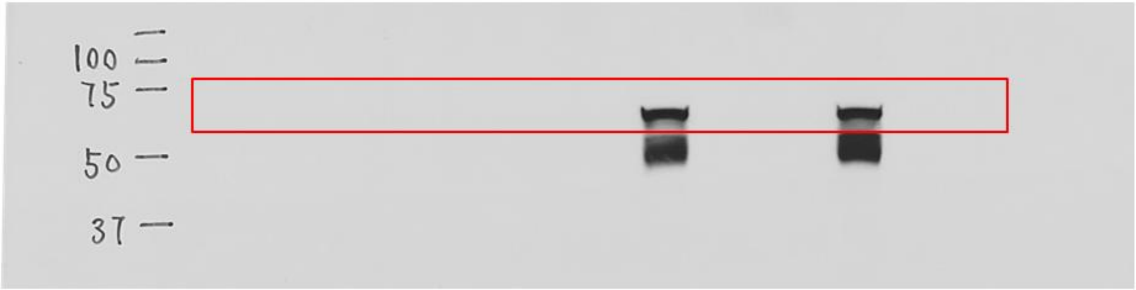

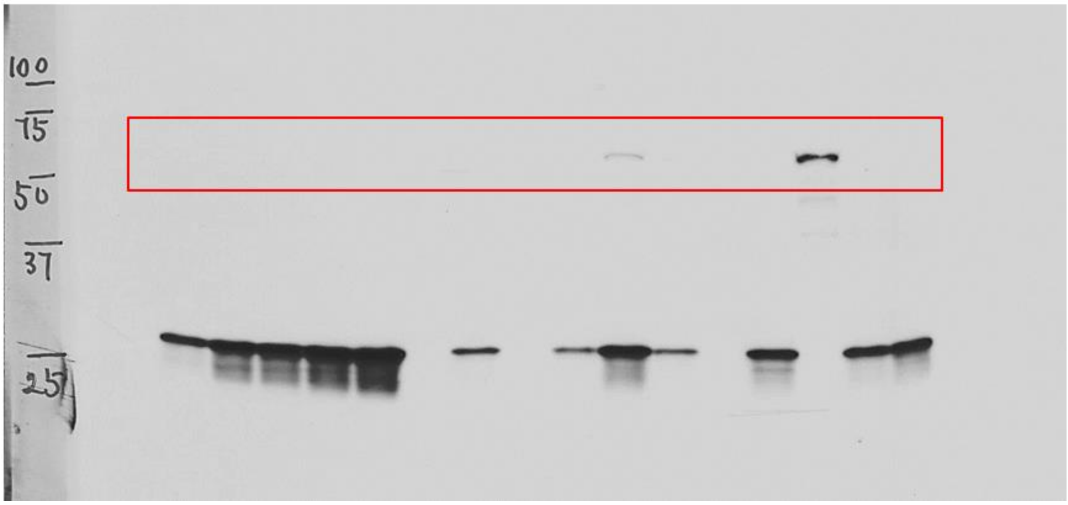

Supplementary Figure S1B

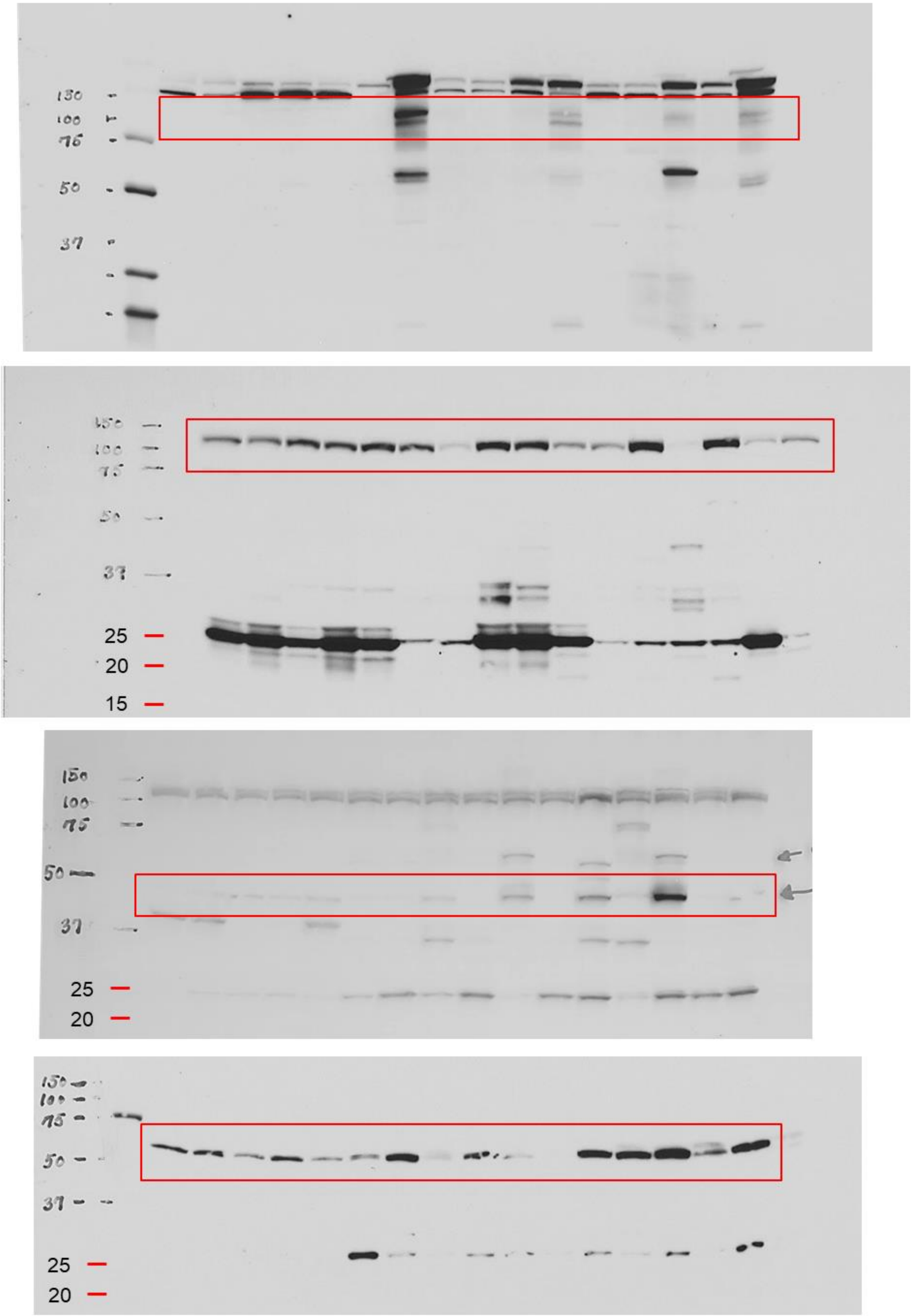

Supplementary Figure S1B

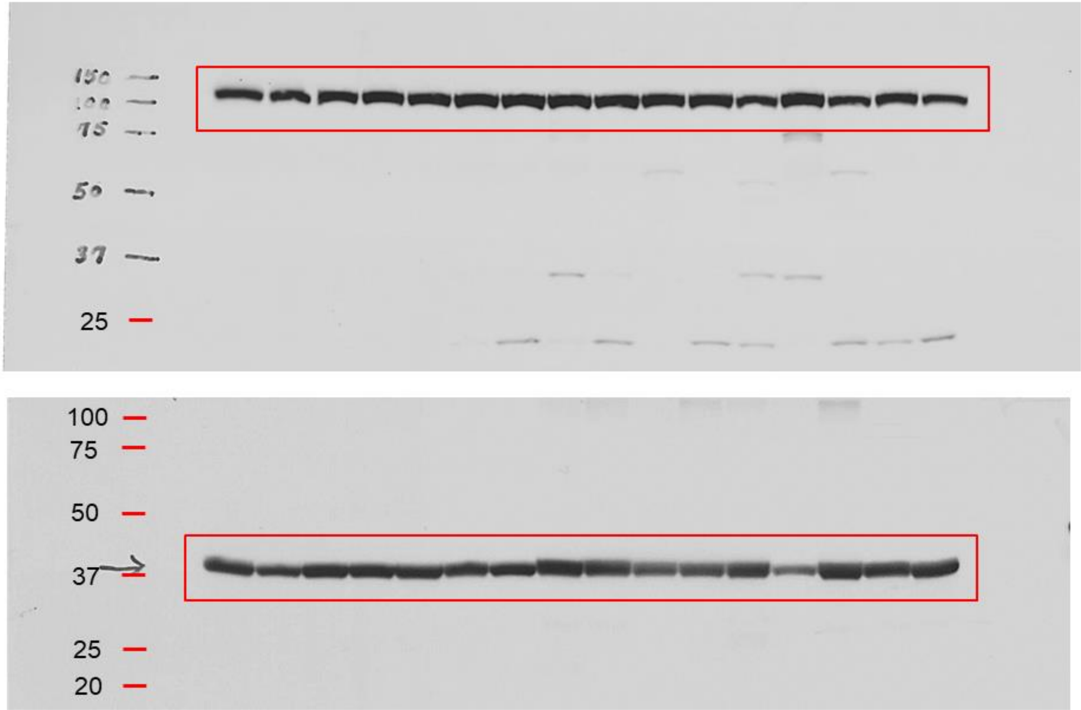

Supplementary Figure S5A

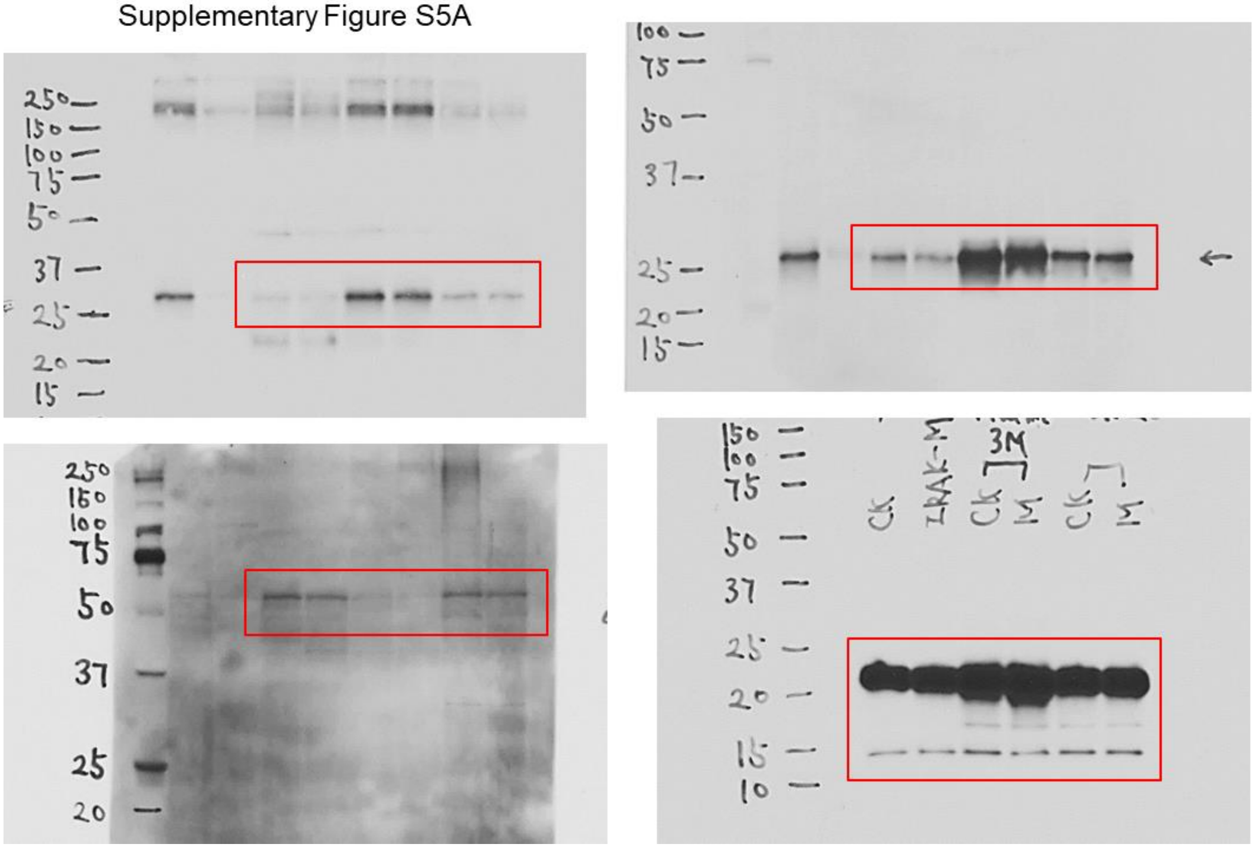

Supplementary Figure S5A

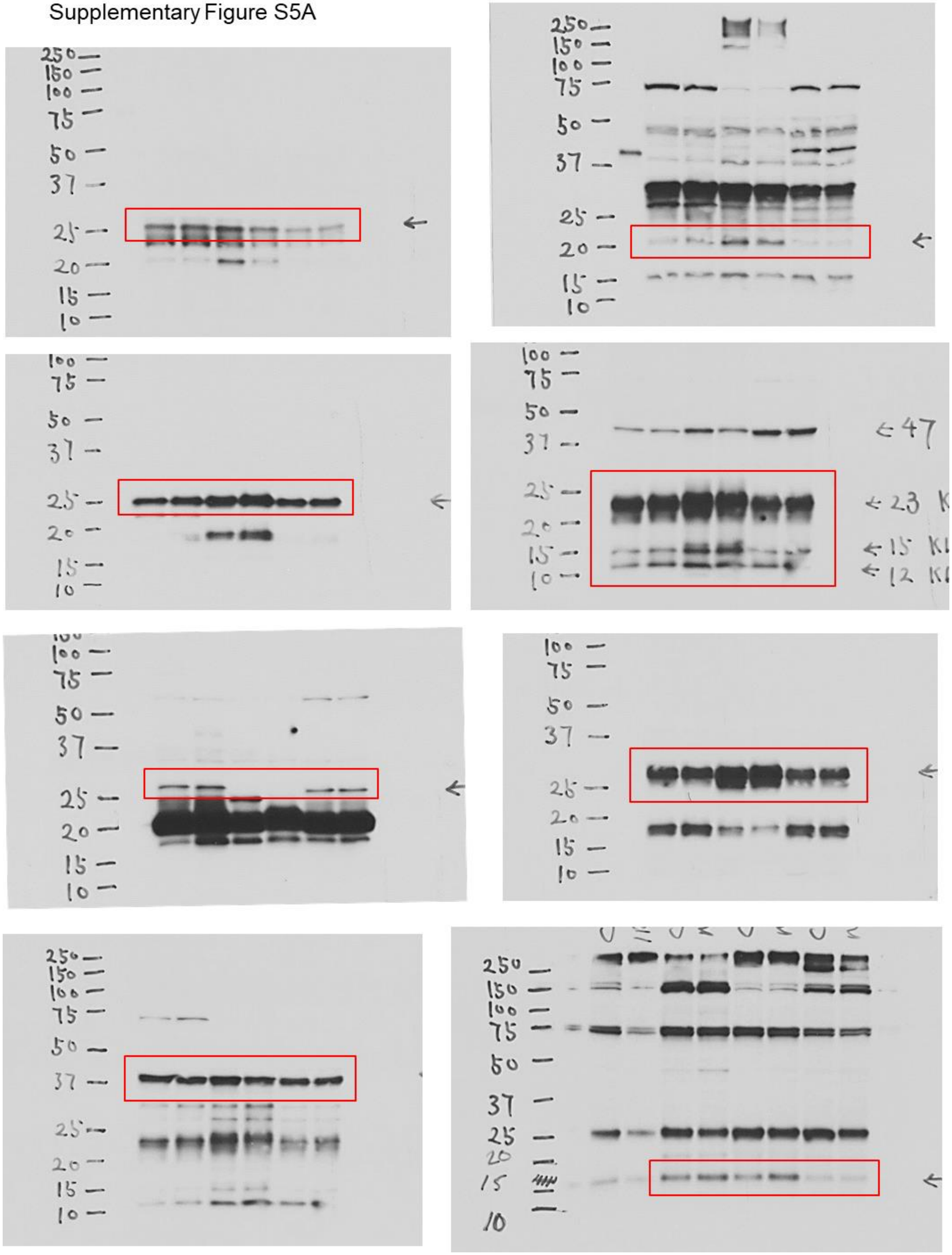

Supplementary Figure S5B

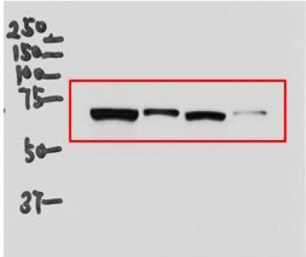

Supplementary Figure S5E

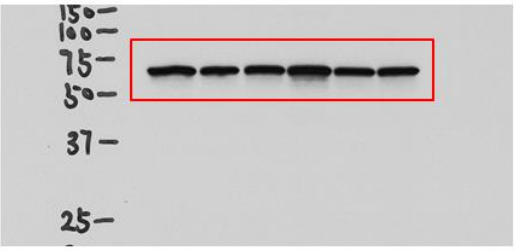

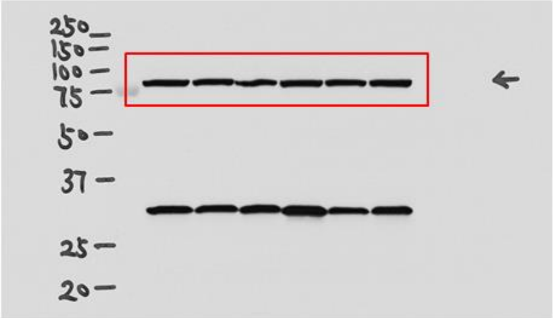

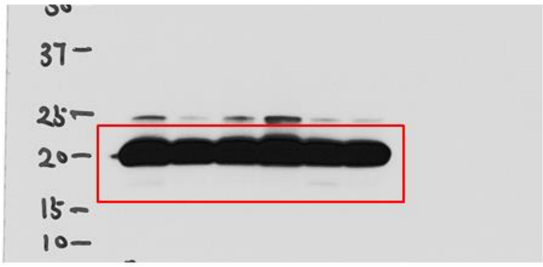

Supplementary Figure S5F

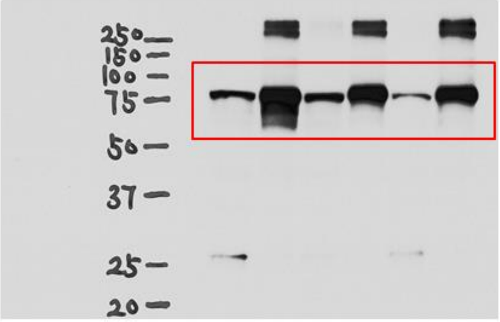

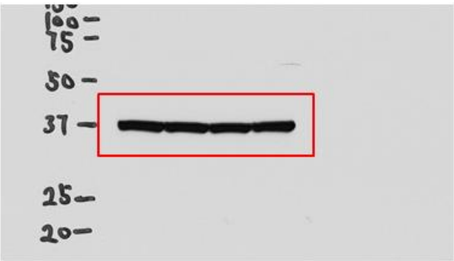

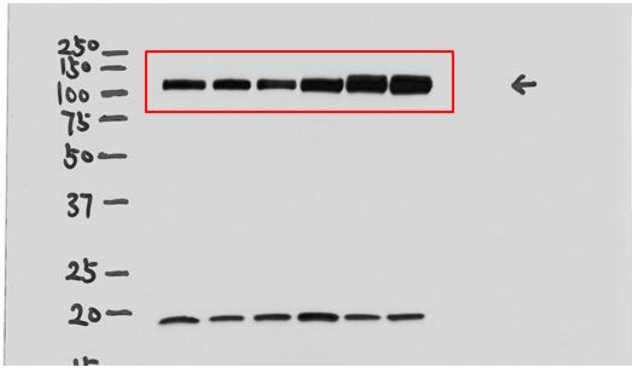

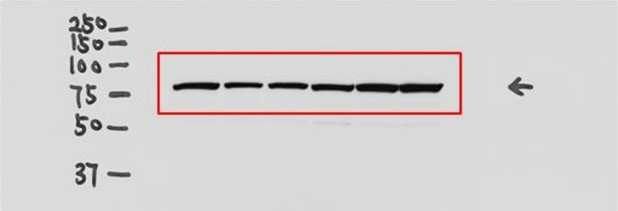

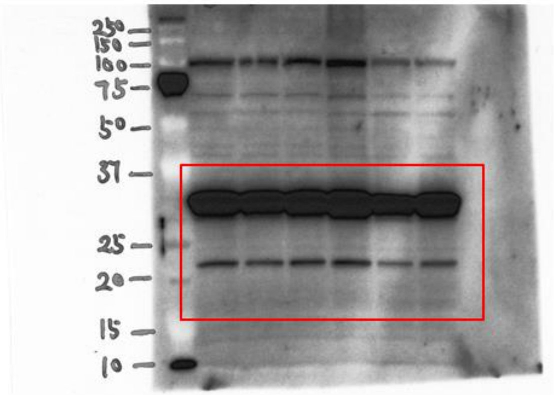

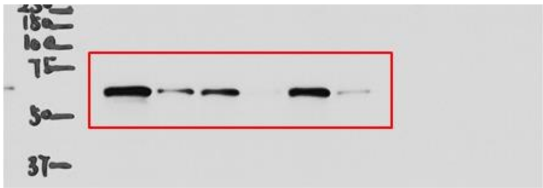

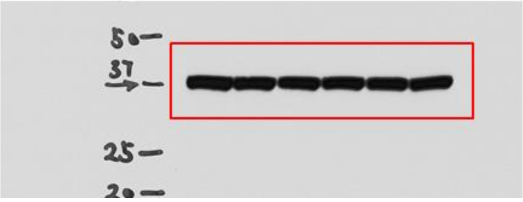

Supplementary Figure S6

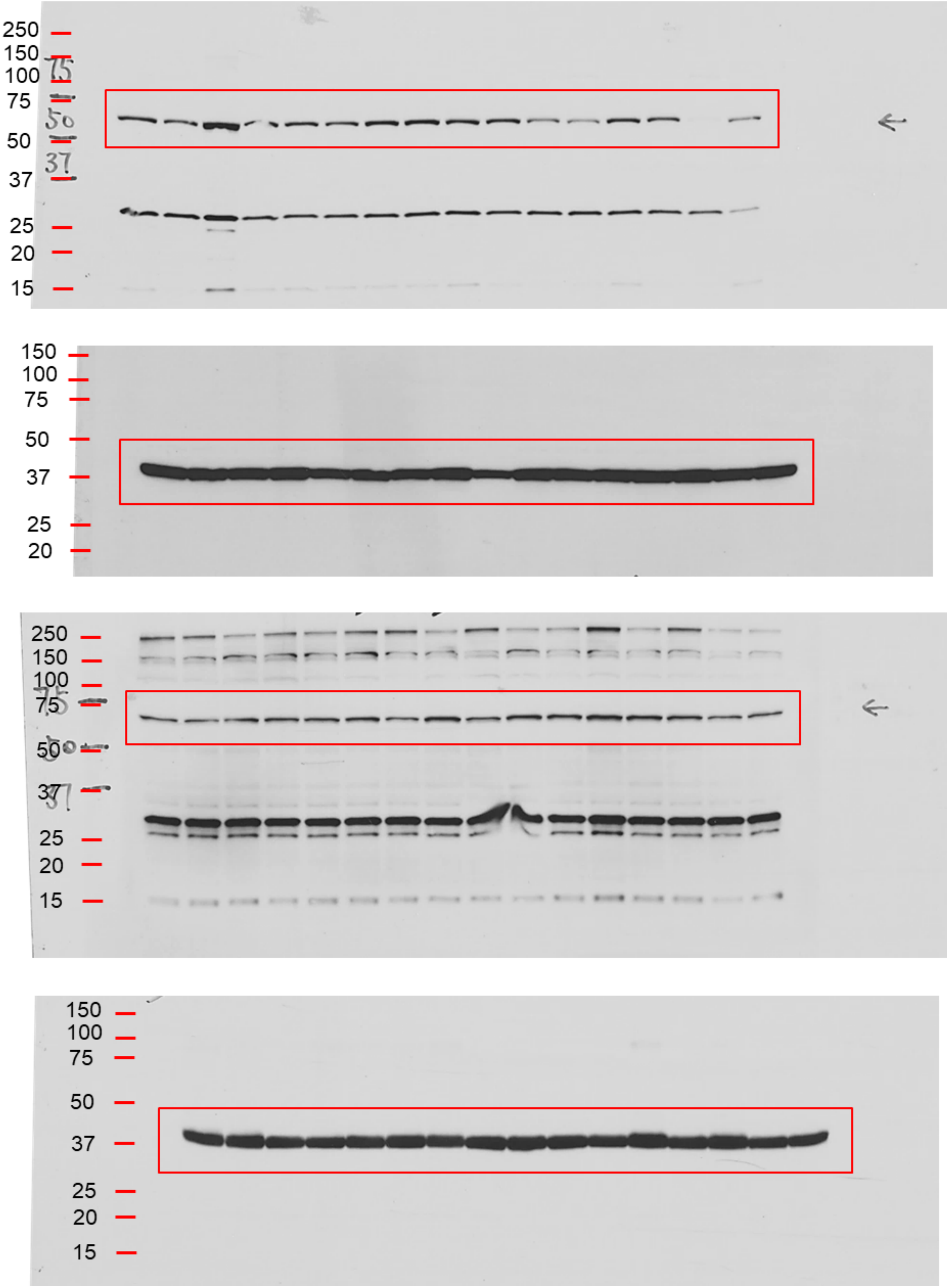

Supplementary Figure S6

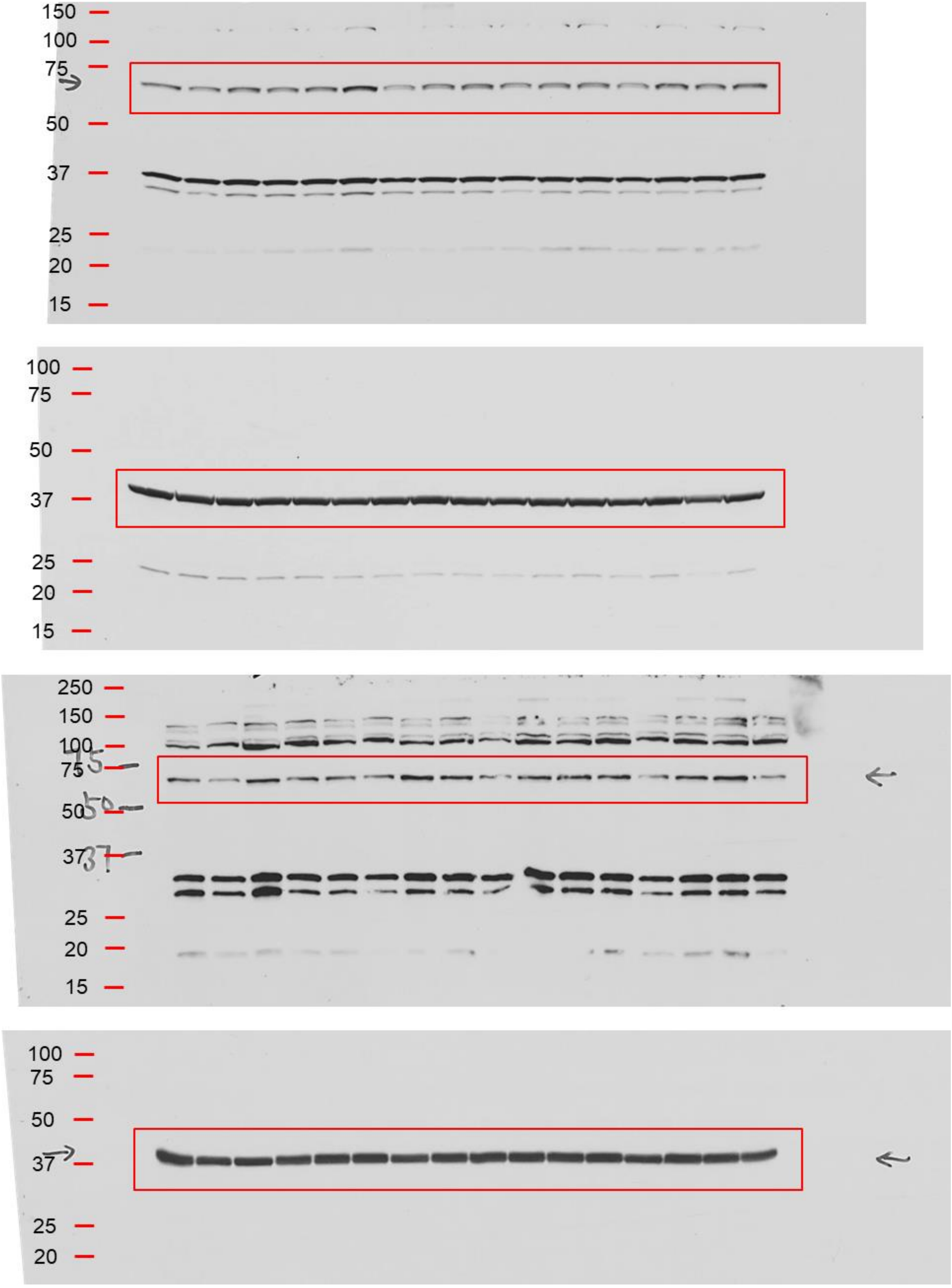

Supplementary Figure S7B

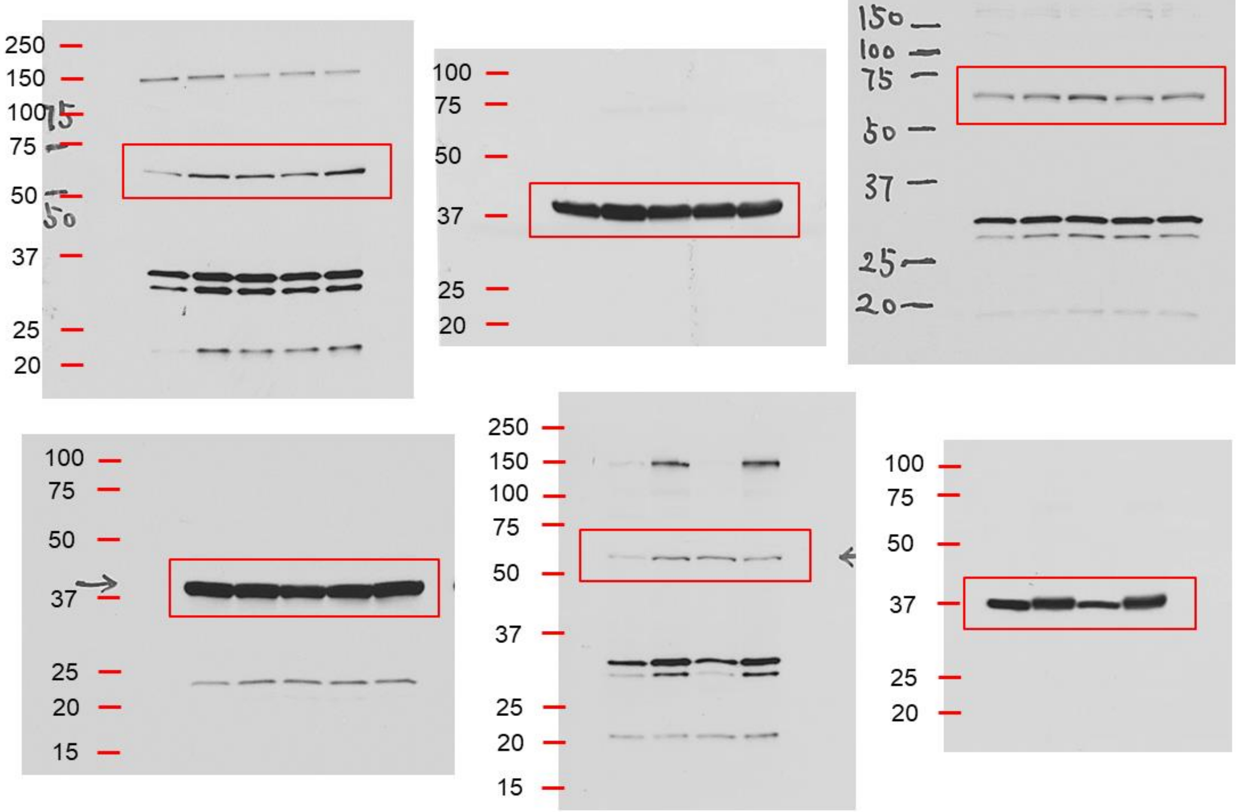

Supplementary Figure S8A

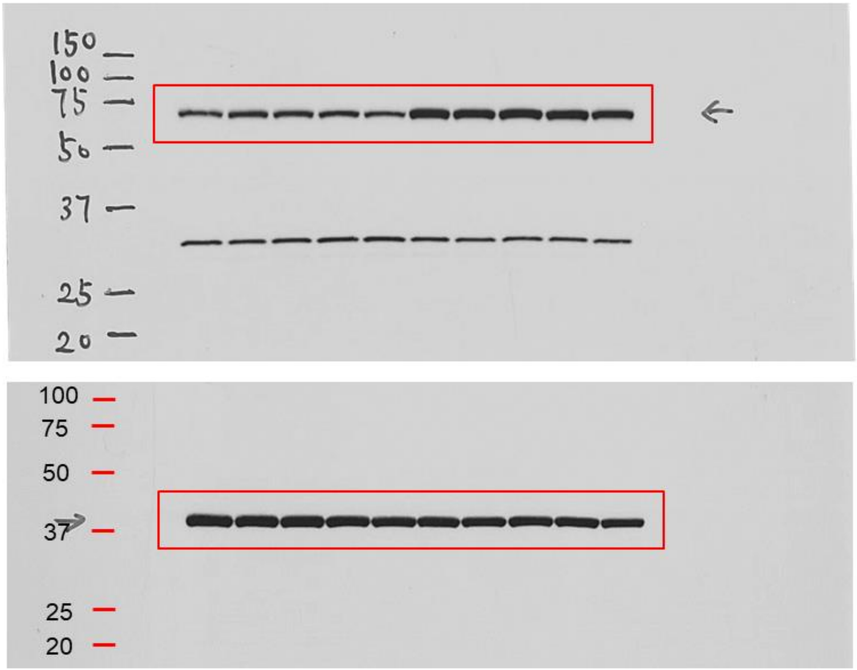

Supplementary Figure S8C

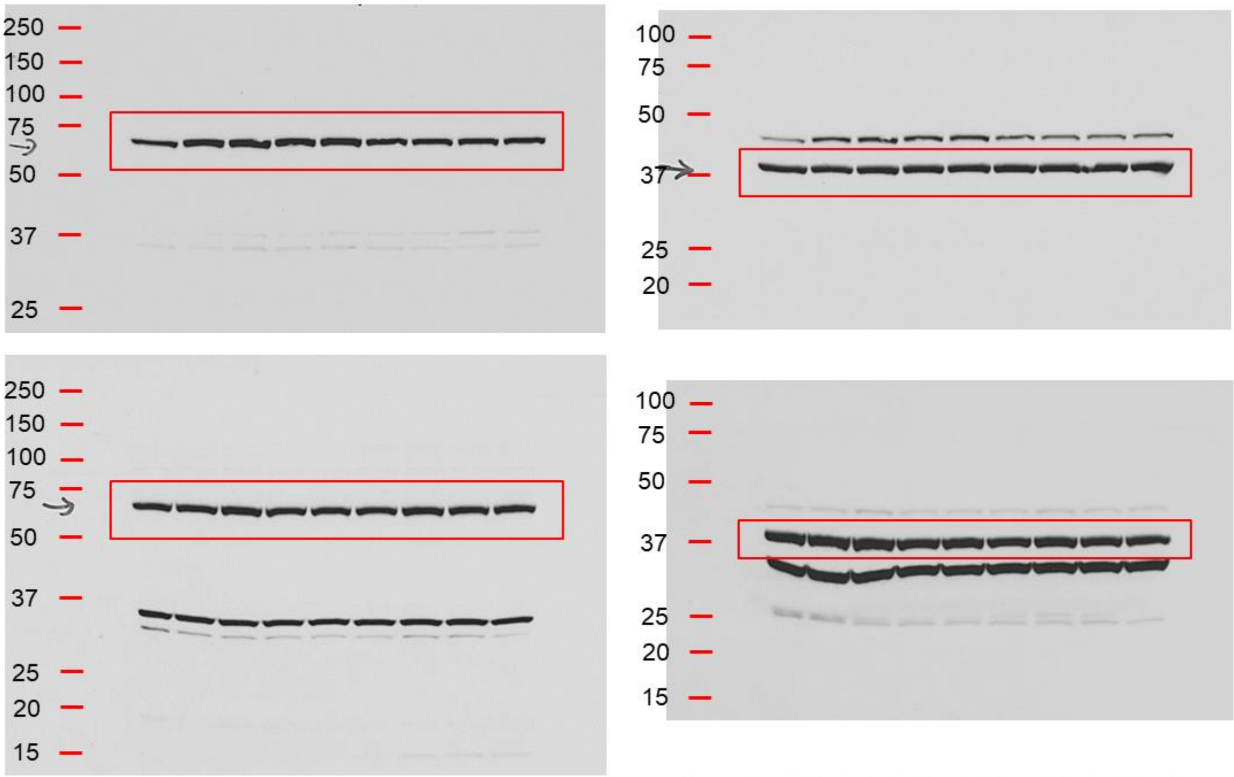

Supplementary Figure S9A

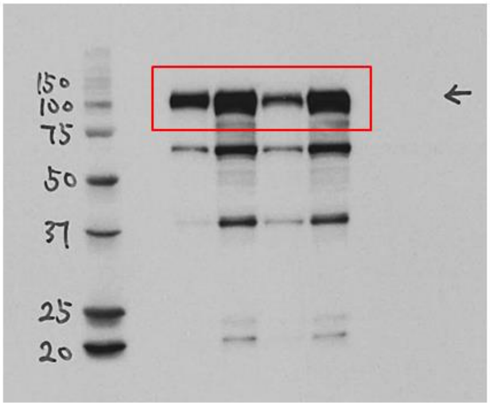

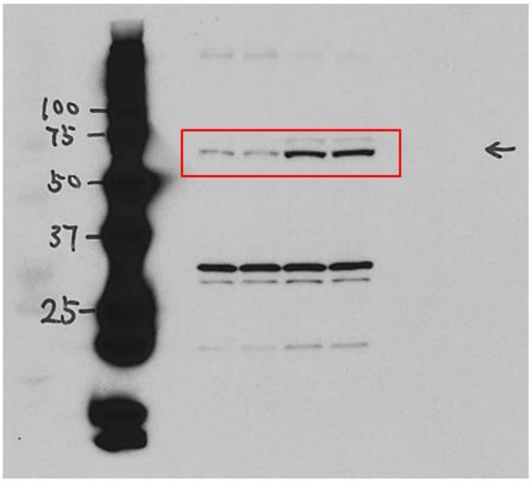

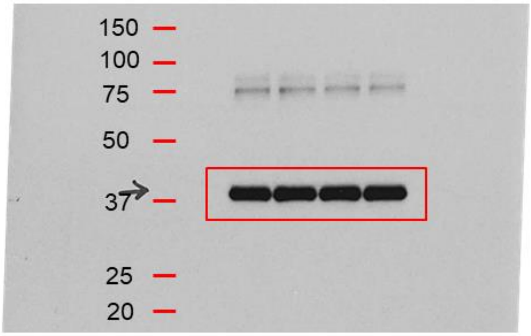

Supplementary Table 1

Cell line	Known variation	Novel variation
A375	No	No
G361	A->G (SNP, -1675); G->A (SNP, -1655); GGAA->---- (INDEL, -1571 to -1568); C->T (SNP, -1452); G->A (SNP, -1422)	No
RPMI7951	No	No
C32	A->G (SNP, -1675); C->T (SNP, -1452); G->A (SNP, -1422)	No
Malme-3	C->G/C (SNP, -2160); A->G/A (SNP, -1675); C->T/C (SNP, -1452); G->A/G (SNP, -1422)	No
Malme-3M	C->G/C (SNP, -2160); A->G/A (SNP, -1675); C->T/C (SNP, -1452); G->A/G (SNP, -1422)	No
624mel	No	No
C8161	C->C/G (SNP, -2160); G->A/G (SNP, -1655); C->T/C (SNP, -1452); G->A/G (SNP, -1422)	A->T/A (-1550)
SK-MEL-2	No	No
SK-MEL-24	C->G (SNP, -2160); A->G (SNP, -1675); C->T (SNP, -1452); G->A (SNP, -1422)	No
SK-MEL-28	C->G (SNP, -2160); A->G (SNP, -1675); C->T (SNP, -1452); G->A (SNP, -1422)	No
SK-MEL-30	C->G (SNP, -2160); A->G (SNP, -1675); G->A/G (SNP, -1655); C->T (SNP, -1452); G->A (SNP, -1422)	A->T/A (-1550)

Supplementary Table 1. Known and novel variations in the promoter region of the IRAK-M gene in human melanoma cell lines. The 2.4 kb 5' flanking region of the human IRAK-M gene was sequenced in eleven melanoma cell lines and one normal skin fibroblast cell line, Malme-3. Sequencing primers are listed in Supplementary Table 2. One novel heterozygous mutation was identified in C8161 and SK-MEL-30 cells after all variations were compared using the dbSNP database of NCBI. The number in parentheses is the location of the variation.

Supplementary Table 2

IlmnID	UCSC RefGene Group	Genomic position	RPMI-7951	C32	Malme-3M	SK-MEL-28	C32 + EPZ	Malme-3M + Aza
cg11599239	TSS1500	66581539	0.893	0.702	0.185	0.77	0.753	0.122
cg15252445	TSS1500	66581866	0.674	0.642	0.117	0.648	0.681	0.108
cg08090769	TSS1500	66582568	0.524	0.535	0.673	0.595	0.573	0.737
cg01183510	TSS1500	66582582	0.789	0.792	0.807	0.822	0.825	0.813
cg18177616	TSS1500	66582737	0.25	0.675	0.318	0.687	0.73	0.229
cg20395892	TS200	66582780	0.218	0.892	0.538	0.888	0.914	0.542
cg11487663	TS200	66582796	0.169	0.875	0.538	0.843	0.905	0.541
cg26279550	TS200	66582805	0.039	0.912	0.35	0.836	0.909	0.274
cg09071340	1stExon	66583002	0.006	0.934	0.056	0.278	0.904	0.026
cg10384554	1stExon	66583015	0.027	0.97	0.091	0.585	0.978	0.045
cg01263292	1stExon	66583029	0.013	0.975	0.014	0.464	0.976	0.01
cg10389229	1stExon	66583203	0.027	0.971	0.097	0.625	0.97	0
cg13807985	Body	66583255	0.081	0.693	0.084	0.547	0.729	0.068
cg07914866	Body	66583431	0.183	0.862	0.291	0.871	0.902	0.295
cg25371434	Body	66584501	0.127	0.853	0.177	0.839	0.899	0.169
cg10900075	Body	66587084	0.875	0.851	0.85	0.871	0.907	0.875
cg17181119	Body	66591119	0.463	0.825	0.169	0.795	0.842	0.128
cg12330267	Body	66606929	0.902	0.883	0.242	0.858	0.915	0.203
cg03812561	Body	66626239	0.805	0.636	0.139	0.741	0.695	0.127
cg08660562	Body	66627900	0.385	0.917	0.445	0.928	0.92	0.407
cg05996573	Body	66627943	0.49	0.839	0.436	0.851	0.854	0.427
cg01194336	Body	66627997	0.538	0.883	0.536	0.808	0.919	0.449
cg06294416	Body	66628232	0.312	0.864	0.429	0.8	0.901	0.31
cg19995654	Body	66628777	0.098	0.905	0.115	0.145	0.919	0.119
cg20162652	Body	66629018	0.123	0.686	0.145	0.138	0.691	0.18
cg19894085	Body	66633274	0.689	0.824	0.128	0.637	0.851	0.126
cg12866960	Body	66635398	0.918	0.918	0.761	0.834	0.913	0.752
cg03564661	3'UTR	66642180	0.878	0.875	0.167	0.806	0.894	0.188

Supplementary Table 2. DNA methylation profiles of IRAK-M gene in RPMI7951, C32, Malme-3M, and SK-MEL-28 cell lines. DNA methylation levels of IRAK-M gene in untreated or drug-treated melanoma cells were analyzed by an Infinium MethylationEPIC BeadChip array. C32 and Malme-3M cells were treated with 50 µM EPZ-6438 (EPZ) and azacytidine (Aza) respectively for 72 hours. Methylation levels are presented as β values. TSS, transcription start site.

Supplementary Table 3. Primers used in this study.

**qPCR**	
Primer name	Sequence (5’ > 3’)
IRAK-M forward	GTGTCCTTCTCCTCTATTCCTG
IRAK-M reverse	TTTGTCCAAGCTCAGAAACATA
GAPDH forward	CTCTCCAGAACATCATCCCT
GAPDH reverse	GTCATCATATTTGGCAGGTTT
**Promoter sequencing**	
Primer name	Sequence (5’ > 3’)
IRAK-M pro1 forward	GGGTTGTGAGTGATTATCTTCT
IRAK-M pro1 reverse	GTCCAGAACAGCGCAGAG
IRAK-M pro2 forward	ATATCGAAATTCTTGCCCACA
IRAK-M pro2 reverse	AGTCAGGTTCTCACTCTGTC
IRAK-M pro3 forward	AAACGAGGAGAAGGAATTGG
IRAK-M pro3 reverse	CTTAGTGACTGGAAGAGTGG
**His-tag**	
Primer name	Sequence (5’ > 3’)
IRAK-M-His forward	TCGTCGACAGGAGGGCCACCATGGCG
IRAK-M-His reverse	ATGCGGCCGCTTAGTGATGGTGGTGATGATGTTCTTTTT TGTACTGTTC
IRAK-M-ΔCTD-His reverse	CTGCGGCCGCTTAGTGATGGTGGTGATGATGTTCAAGA GTATTTAAAAC
TRAF6-His forward	TCGTCGACAGGAGGGCCACCATGAGTCTGCTAAACTGT GAA
TRAF6-His reverse	CTGCGGCCGCCTAGTGATGGTGGTGATGATGTACCCCT GCATCAGTACT
CAST-His forward	TCGTCGACGCCATGTCCCAGCCCGGCCAGAAG
CAST-His reverse	CTGCGGCCGCTTAGTGATGGTGGTGATGATGGTCATCTT TTGGCTTGGA
