# Supplementary material for: Induction of IRAK-M in melanoma induces caspase-3 dependent apoptosis by reducing TRAF6 and calpastatin levels
Source: Commun Biol. 2020 Jun 12;3:306. doi: 10.1038/s42003-020-1033-y (PMC7293221; doi:10.1038/s42003-020-1033-y)
Supplement: Supplementary file 2 — Description of Additional Supplementary Files [file 42003_2020_1033_MOESM2_ESM.pdf]

Description of Additional Supplementary Files

File name: Supplementary Data

Description: Supplementary data file contains the source data underlying each graph presented in the article.

Data for Figure 1a

Data for Figure 1b

Data for Figure 1d

Data for Figure 1e

Data for Figure 1f

Data for Figure 1g

Data for Figure 2b

Data for Figure 2d

Data for Figure 2e

Data for Figure 3b

Data for Figure 3d

Data for Figure 3e

Data for Figure 3g

Data for Figure 3h

Data for Figure 4b

Data for Figure 4c

Data for Figure 4e

Data for Figure 4f

Data for Figure 5a

Data for Figure 5c

Data for Figure 5d

Data for Figure 5f

Data for Figure 6a
