# Supplementary material for: Induction of IRAK-M in melanoma induces caspase-3 dependent apoptosis by reducing TRAF6 and calpastatin levels
Source: Commun Biol. 2020 Jun 12;3:306. doi: 10.1038/s42003-020-1033-y (PMC7293221; doi:10.1038/s42003-020-1033-y)
Supplement: Supplementary file 4 — Reporting Summary [file 42003_2020_1033_MOESM4_ESM.pdf]

nature research

Corresponding author(s): Eduardo Davila

Last updated by author(s): Apr 27, 2020

Reporting Summary

Nature Research wishes to improve the reproducibility of the work that we publish. This form provides structure for consistency and transparency in reporting. For further information on Nature Research policies, see Authors & Referees and the Editorial Policy Checklist.

Statistics

For all statistical analyses, confirm that the following items are present in the figure legend, table legend, main text, or Methods section.

n/a	Confirmed	
☐	☒	The exact sample size (*n*) for each experimental group/condition, given as a discrete number and unit of measurement
☐	☒	A statement on whether measurements were taken from distinct samples or whether the same sample was measured repeatedly
☐	☒	The statistical test(s) used AND whether they are one- or two-sided<br>*Only common tests should be described solely by name; describe more complex techniques in the Methods section.*
☒	☐	A description of all covariates tested
☐	☒	A description of any assumptions or corrections, such as tests of normality and adjustment for multiple comparisons
☐	☒	A full description of the statistical parameters including central tendency (e.g. means) or other basic estimates (e.g. regression coefficient) AND variation (e.g. standard deviation) or associated estimates of uncertainty (e.g. confidence intervals)
☐	☒	For null hypothesis testing, the test statistic (e.g. *F*, *t*, *r*) with confidence intervals, effect sizes, degrees of freedom and *P* value noted<br>*Give P values as exact values whenever suitable.*
☒	☐	For Bayesian analysis, information on the choice of priors and Markov chain Monte Carlo settings
☒	☐	For hierarchical and complex designs, identification of the appropriate level for tests and full reporting of outcomes
☐	☒	Estimates of effect sizes (e.g. Cohen's *d*, Pearson's *r*), indicating how they were calculated

Our web collection on statistics for biologists contains articles on many of the points above.

Software and code

Policy information about availability of computer code

Data collection	Microsoft Excel 2010, GraphPad Prism 7, ImageJ
Data analysis	Log-rank (Mantel-Cox) test, unpaired two-tailed Student’s t-test, Pearson’s correlation test, two-way ANOVA

For manuscripts utilizing custom algorithms or software that are central to the research but not yet described in published literature, software must be made available to editors/reviewers. We strongly encourage code deposition in a community repository (e.g. GitHub). See the Nature Research guidelines for submitting code & software for further information.

Data

Policy information about availability of data

All manuscripts must include a data availability statement. This statement should provide the following information, where applicable:

- Accession codes, unique identifiers, or web links for publicly available datasets
- A list of figures that have associated raw data
- A description of any restrictions on data availability

mRNA expression and DNA methylation profiles data for melanoma cell lines presented in Fig. 1a and Supplementary Figs. 1a, 3c are available at the Cancer Cell Line Encyclopedia (CCLE) (https://portals.broadinstitute.org/ccle). Melanoma patient survival data in Fig. 1b are from The Cancer Genome Atlas (TCGA) (https://portal.gdc.cancer.gov/), RNA-seq gene expression, DNA methylation, and genetic alterations data for melanoma patient samples in Fig. 1g and Supplementary Fig. 3a from http://www.cbioportal.org. IRAK-M microarray gene expression profiles data for cancer patient samples in Figs. 1d, 1f and Supplementary Figs. 2c, 3b are available at NCBI’s Gene Expression Omnibus (GEO, https://www.ncbi.nlm.nih.gov/geo/). DNA methylation levels of IRAK-M gene in Supplementary Fig. 4 and Supplementary Table 2 is available in the GEO database (accession number GSE143614).

Field-specific reporting

Please select the one below that is the best fit for your research. If you are not sure, read the appropriate sections before making your selection.

☒ Life sciences ☐ Behavioural & social sciences ☐ Ecological, evolutionary & environmental sciences

For a reference copy of the document with all sections, see nature.com/documents/nr-reporting-summary-flat.pdf

Life sciences study design

All studies must disclose on these points even when the disclosure is negative.

Sample size	All in vitro tests were performed in a minimum of triplicate. There were at least 5 mice per group in independent mouse experiments.
Data exclusions	No data were excluded.
Replication	Most in vitro experiments were repeated three times as stated in the figure legends. In vivo antitumor experiments were carried out twice.
Randomization	Cells and mice were randomly assigned to experimental groups.
Blinding	The investigators were not blinded to group allocation during experiments and data analysis.

Reporting for specific materials, systems and methods

We require information from authors about some types of materials, experimental systems and methods used in many studies. Here, indicate whether each material, system or method listed is relevant to your study. If you are not sure if a list item applies to your research, read the appropriate section before selecting a response.

Materials & experimental systems

n/a	Involved in the study	
☐	☒	Antibodies
☐	☒	Eukaryotic cell lines
☒	☐	Palaeontology
☐	☒	Animals and other organisms
☒	☐	Human research participants
☒	☐	Clinical data

Methods

n/a	Involved in the study	
☒	☐	ChIP-seq
☐	☒	Flow cytometry
☒	☐	MRI-based neuroimaging

Antibodies

Antibodies used	IRAK-M (#4369), Bax (#2772), Smac/Diablo (#15108; clone D5S3R), cytochrome c (#4272), caspase-3 (#9668; clone 3G2), caspase-8 (#4790; clone D35G2), caspase-9 (#9508; clone C9), TRAF6 (#8028; clone D21G3), calpastatin (#4146), calpain 1 (#2556), calpain 2 (#2539), p-FADD (#2781), FADD (#2782), Bid (#2002), p-Bad (#9296; clone 7E11), Bad (#9292), Bak (#12105; clone D4E4), Bim (#2933; clone C34C5), Bcl2 (#4223; clone D55G8), Bcl-XL (#2764; clone 54H6), Mcl1 (#4572), survivin (#2808; clone 71G4B7), SOCS1 (#3950; clone A156), Tollip (#4748), p-SHP-1 (#8849; clone D11G5), SHP-1 (#3759; clone C14H6), A20 (#4625), CYLD (#8462; clone D1A10), TANK (#2141), USP4 (#2651), His-Tag (#2365), His-Tag (#4079; clone 27E8), K63-linkage polyubiquitin (#5621; clone D7A11), and GAPDH (#2118; clone 14C10) antibodies were from Cell Signaling Technology. SIGIRR (#ab233146), ST2 (#ab25877), p-PTP1B (#ab88472), PTP1B (#ab88481), and TRIM38 (#ab69977) antibodies from Abcam.
Validation	All commercially available antibodies were validated by manufacturers.

Eukaryotic cell lines

Policy information about cell lines

Cell line source(s)	A375, G361, RPMI7951, C32, Malme-3M, SK-MEL-2, SK-MEL-24, SK-MEL-28, A101D, and HS294T cell lines were purchased from ATCC. 624 Mel and C8161 cell lines were from Dr. Suzanne Ostrand-Rosenberg (University of Maryland, Baltimore County) and SK-MEL-30 from Dr. Thomas Hornyak (University of Maryland, Baltimore). Mel-neo and Mel-adu cells were from ATCC, HEMn-MP, HEMn-DP and HEMa-LP cells from Cascade Biologics.
Authentication	By microscopic morphology
Mycoplasma contamination	Not tested for mycoplasma contamination.

Commonly misidentified lines (See ICLAC register)	SK-MEL-28 cell line. SK-MEL-28 was one of tested human melanoma cell lines in which IRAK-M was lowest expressed. Also, restoring IRAK-M expression induced apoptosis in SK-MEL-28 cells.

Animals and other organisms

Policy information about studies involving animals; ARRIVE guidelines recommended for reporting animal research

Laboratory animals	NOD-SCID mice, female, 4-6 weeks of age.
Wild animals	not used
Field-collected samples	not used
Ethics oversight	All mouse experiments were conducted in strict accordance with protocols approved by the IACUC of the University of Maryland Baltimore School of Medicine.

Note that full information on the approval of the study protocol must also be provided in the manuscript.

Flow Cytometry

Plots

Confirm that:

☒ The axis labels state the marker and fluorochrome used (e.g. CD4-FITC).

☒ The axis scales are clearly visible. Include numbers along axes only for bottom left plot of group (a 'group' is an analysis of identical markers).

☒ All plots are contour plots with outliers or pseudocolor plots.

☒ A numerical value for number of cells or percentage (with statistics) is provided.

Methodology

Sample preparation	Melanoma cells were collected and washed with 1xPBS once at 1,200 rpm for 5 minutes. Cells were resuspended with 100 µL of 1×Annexin V binding buffer and stained with 5 µL of APC-Annexin V and 0.5 µL of PI for 15 minutes at 4 ºC. Then, cells were washed with 1×Annexin V binding buffer twice at 1,200 rpm for 5 minutes, resuspended with 400 µL binding buffer, and analyzed by flow cytometry.
Instrument	BD LSR II
Software	FlowJo
Cell population abundance	10,000 events per sample were recorded.
Gating strategy	Gating strategy is shown in Supplementary Figure 8b. Live cells are in Annexin V-/PI- quadrant, early apoptotic cells in Annexin V +/PI- quadrant, late apoptotic cells in Annexin V+/PI+ quadrant, and necrotic cells in Annexin V-/PI+ quadrant.

☒ Tick this box to confirm that a figure exemplifying the gating strategy is provided in the Supplementary Information.
